# Fibroblasts as Turned Agents in Cancer Progression

**DOI:** 10.3390/cancers15072014

**Published:** 2023-03-28

**Authors:** Robert Wieder

**Affiliations:** Rutgers New Jersey Medical School and the Cancer Institute of New Jersey, Newark, NJ 07103, USA; wiederro@njms.rutgers.edu; Tel.: +1-973-972-4871

**Keywords:** transformation, tumor stroma, microenvironment, heterogeneity, cancer-associated fibroblasts, therapeutic targets

## Abstract

**Simple Summary:**

Normal epithelial cells in our organs are surrounded by stroma, which is an ecosystem made up of a variety of cells and proteins that support their intended functions. When normal epithelial cells go through a series of genetic steps that transform them into cancer cells, the cancer cells, in turn, change the character of the stroma. The stroma co-evolves with the cancer to become an autonomous tumor organ with its own ecosystem. Initially, stromal cells try to maintain normal epithelial cell function and suppress malignant changes. However, during their co-evolution, the cancer cells recruit supporting cells to actively promote their growth, invasion, metastasis, and treatment resistance. Here, I outline how cancer cells change the character of the most abundant cells in the stroma, called fibroblasts, and how the altered fibroblasts, in turn, make cancers more aggressive. I also outline efforts to use these changed fibroblasts as new targets for cancer treatment.

**Abstract:**

Differentiated epithelial cells reside in the homeostatic microenvironment of the native organ stroma. The stroma supports their normal function, their G_0_ differentiated state, and their expansion/contraction through the various stages of the life cycle and physiologic functions of the host. When malignant transformation begins, the microenvironment tries to suppress and eliminate the transformed cells, while cancer cells, in turn, try to resist these suppressive efforts. The tumor microenvironment encompasses a large variety of cell types recruited by the tumor to perform different functions, among which fibroblasts are the most abundant. The dynamics of the mutual relationship change as the sides undertake an epic battle for control of the other. In the process, the cancer “wounds” the microenvironment through a variety of mechanisms and attracts distant mesenchymal stem cells to change their function from one attempting to suppress the cancer, to one that supports its growth, survival, and metastasis. Analogous reciprocal interactions occur as well between disseminated cancer cells and the metastatic microenvironment, where the microenvironment attempts to eliminate cancer cells or suppress their proliferation. However, the altered microenvironmental cells acquire novel characteristics that support malignant progression. Investigations have attempted to use these traits as targets of novel therapeutic approaches.

## 1. Introduction

Malignant transformation of differentiated cells begins with the acquisition of genomic instability [1] that leads to eventual mutations in one or two tumor suppressor genes, which enable cells to pass on acquired mutations with each division [2]. Cells acquire a malignant phenotype generally after they develop sustained mutations in two to eight genes, out of a total of about 140 genes able to drive tumorigenesis [3]. These genes fall into twelve signaling pathways, categorized into three broad functional groups: regulation of cell fate, cell survival, and maintenance of the genome [3]. The mutations required to generate fully malignant cells endow them with tumor-initiating or cancer stem cell properties, with characteristics that permit epithelial cells to discard symmetry, decrease adhesion, divide indefinitely, avoid senescence, resist growth suppression signaling, and resist immune destruction [4,5]. Tumor-initiating cells are generated through a spectrum of gene expression steps of the epithelial–mesenchymal transition program, where the transformed cells dysregulate cell death mechanisms, adopt metabolic capabilities that enable bioenergetic survival at reduced oxygen tension and pH, and alter gene expression programs that enable motility, invasion, and angiogenesis, among others [6,7,8].

Once cancer cells become malignant, they continue to mutate due to their genomic instability and proceed to generate tumors with exceptional heterogeneity [9]. Tumors eventually subtend hundreds to thousands of clones that have accumulated and continue to accumulate mutations that contribute to therapy resistance [9]. However, while cancers become highly heterogeneous, the original driver mutations remain the ones responsible for the progression of the primary tumor and metastatic lesions [10].

Malignant transformation of differentiated epithelial cells occurs in parallel with that of the microenvironment. The microenvironment of a differentiated organ consists of a variety of cells that together support the normal physiologic functions of epithelial cells [11]. The stromal cells consist of primarily fibroblasts but also include myoepithelial cells, endothelial cells, macrophages, T-cells, neutrophils, and mast cells [12]. The microenvironment also includes non-cellular elements, such as collagens, laminins, and fibronectin, as well as a spectrum of soluble cytokines, growth factors, and hormones produced locally or externally to the organ [13]. The elements of the normal microenvironment collectively suppress the progression, malignant behavior, and survival of transformed epithelial cells as they transition to invasive cancer [14].

In turn, malignant cells modify normal fibroblasts (NFs), endothelial cells, macrophages, and immune cells that reside in the microenvironment, and recruit and modify additional cells from outside the tumor to promote inflammation, immune suppression, treatment resistance, angiogenesis, invasion, metastasis [15,16,17] and the immune microenvironment, which engenders exceptional plasticity in their mission to support cancer progression [18,19,20,21]. Co-evolution of the cancer with its supporting cellular structures involves coordinate modification of supporting cells by the cancer, by reciprocal effects of modified supporting cells on each other and in turn, their communal effects on cancer cells [22]. Non-orchestrated, multiple, diverse effects applied by transformed epithelial cells on stromal cells are fostered by the permissive bioenergetic and physical characteristics of solid tumors [23].

The newly acquired characteristics and heterogeneity of stromal cells pose obstacles to multimodal therapy, including immune therapy [24]. However, their distinct characteristics also provide newly-appreciated opportunities to expand the targets of cancer therapeutics [25,26]. Here, I review the literature on characteristics of normal tissue primary and metastatic microenvironments and their suppressive effects on transformed cells, with a primary focus on NFs. I also review how cancer modifies these microenvironments and their resident cells, also with a focus on fibroblasts, while fully recognizing that all of the elements of the microenvironment are affected by the cancer cells. I discuss how modified fibroblasts become agents of cancer cells to further promote their malignant potential. Finally, I discuss approaches that have been considered in targeting the tumor microenvironment for therapy. In recognition of this reality, cancer clinical trials have started to target stromal elements, and some have been completed, yielding mixed results [27,28].

## 2. Normal Tissue Fibroblasts

### 2.1. Origin of Adult Organ Fibroblasts

Fibroblasts, the primary stromal cells of mesenchymal embryonic origin in normal adult differentiated organs, have been investigated extensively [29]. They are present in all tissues and generate the structural features of organs by producing and remodeling the extracellular matrix (ECMs) [30]. They serve as heterogeneous, peripatetic, lineage origin-specific, and venue-specific adaptable tools that are used and modified in order to support the mission and needs of the epithelial cell defining the adult differentiated organ (comprehensively reviewed by LeBleu et al. (2020) [29]. They regulate development, morphogenesis, homeostasis, tissue repair, maintenance of stem cells, and the function of immune cells, endothelial cells, and other matrix-resident cells, with specific functional roles based on origin, location, and gene expression patterns [12,31,32]. In cancer, they initially try to suppress and contain the tumor, and once re-tasked by the cancer, they perform supportive roles in malignant progression [13].

Originally, fibroblasts develop in the primary mesenchyme during gastrulation [29]. As the mesenchyme differentiates into the three embryonic layers, more mature fibroblasts develop during the development of the true mesenchyme from the mesoderm, along with endothelial cells, adipocytes, and pericytes, all cells capable of initiating mesenchymal programs in response to signaling cues [29]. These fibroblasts are quiescent, respond to stimulation, and have the ability to regenerate and maintain the ECM and the structural stability of their tissue [29]. As an example of the most frequently investigated tissue site, mature dermal fibroblasts are quiescent but metabolically active, producing ECM proteins collagen I, fibronectin, elastin, periostin, and tenascin-C [29,33]. Upon injury, they resume proliferation in response to signals such as prostaglandins and cytokines, including transforming growth factor (TGF)β and platelet-derived growth factor (PDGF) [29,33]. They also retain the ability to dedifferentiate and form pluripotential stem cells [34,35]. Collectively, these abilities, their gene expression profiles, and interactions with other cells in the stroma are organ-, origin- and age-specific and remain subjects of continued investigations [36,37,38,39].

Other supporting cells of the normal differentiated organ extracellular matrix derive from mesenchymal stem cells (MSCs), which are differentiated tissue-specific multilineage progenitors that sustain cell renewal of their tissue for the duration of its life [40,41], with three minimum criteria defined by the International Society of Cellular Therapy (ISCT) [42]. The criteria specify that cells (1) must be plastic-adherent in standard culture conditions, (2) must express cluster of differentiation (CD)73, CD90, and CD105, must not express CD11b or CD14, CD19 or CD79α, CD34, CD45, and HLA-DR, and (3) must have the capacity to differentiate to osteoblasts, adipocytes, and chondroblasts in vitro [42]. MSCs were originally identified in the bone marrow (BM MSCs) [41,43], but since then, studies have demonstrated that a subset of mesenchymal cells remains in adult tissue [40]. These MSCs have been found in perivascular sites of almost all adult tissues, including adipose tissue, liver, kidney, lung, brain, breast, ovaries, placental tissue, menstrual and umbilical cord blood, testes, liver, lung, pancreas, spleen, dental pulp, dermis, and others [43,44,45]. Indeed, reports have demonstrated that adult organ MSCs have similar pluripotential capacities to BM MSCs and are able to differentiate into bone, cartilage, adipose tissue, tendon, muscle fibroblasts and myofibroblasts, neurons, and cardiomyocytes [40,44,45]. They serve the differentiated tissue through indispensable roles in self-renewal, repair and maintenance of normal function, immune modulation, and wound healing, and as a constant source for fibroblasts and myofibroblasts (comprehensively reviewed by Frisbie et al. (2022) [46]. Population and single-cell mRNA sequencing have demonstrated that both inter- and intra-tissue heterogeneity, gene expression, signaling pathway activation, and differentiation potentials of MSCs are exceptionally diverse, and serve the needs of the differentiated organs [12,47,48]. Single-cell transcriptomic sequencing of bone marrow and umbilical cord Wharton’s jelly MSCs revealed five distinct subpopulations that differentiate from stem-like active proliferative cells (APCs) [49]. These subpopulations express the perivascular mesodermal progenitor markers chondroitin sulfate proteoglycan 4 (CSPG4)/melanoma cell adhesion molecule-MUC18 (MCAM)/nestin (NES) of multipotent progenitor cells [49]. Subsequently, they branch off into either adipogenesis or osteochondrogenesis and differentiate into unipotent prechondrocytes specifically expressing immunomodulatory genes able to suppress activated CD3^+^ T cell proliferation [49].

Myofibroblasts are specialized contractile fusiform fibroblasts considered to be the activated forms of fibroblasts [50]. Myofibroblasts secrete and organize the extracellular matrix in normal tissue, which includes laminins, fibronectin, and collagens I, III, and IV, and generate tissue fibrosis in abnormal circumstances [32,51,52]. They are identifiable by the expression of vimentin and of α-smooth muscle actin (αSMA) on their stress fibers, which induce contraction in wound healing in response to tissue traction stress and to TGF-β, interleukin (IL)-6, PDGF, and fibroblast growth factor (FGF)-2 secretion by platelets and macrophages [32,50,51,52]. Although myofibroblasts can originate from local MSCs or BM-derived MSCs, they generally originate from connective tissue fibroblasts [51].

MSCs and normal tissue fibroblasts are very similar in molecular markers, pluripotency, proliferative capacities, and transcriptomes [53,54]. Fibroblasts continue to have de-differentiation potential in vitro and can be induced to form pluripotent stem cells (iPSCs) [35]. Normal tissue fibroblasts in the extracellular matrix also maintain some capacity to de-differentiate and to be reprogrammed into iPSCs [34]. Fibroblasts, similar to MSCs, can differentiate into adipocytes, chondrocytes, and osteoblasts [55]. In the context of these similarities, MSCs deposit extracellular matrices of collagen type I and fibronectin and express fibroblast-specific protein (FSP)1 in patterns similar to fibroblasts [55]. With respect to surface markers, cultured normal foreskin and embryonic lung fibroblasts and cultured primary BM MSCs express the same cell surface population-averaged surface markers [55]. These are the MSC markers CD73, CD90, and CD105, which are part of the ISCT MSC consensus criteria, and MSC markers CD29, and CD44, which are not part of the consensus criteria [55]. All the cells are also negative for CD14, CD19, CD31, CD34, CD45, and HLA-DR [55], and negative or equivocal for CD9, CD10, CD106, CD146, CD271, SSEA4, and Stro-1 [53]. Phenotypic markers of fibroblasts also include PDGFRα/β, podoplanin, caveolin (CAV)1, discoidin domain receptor 2 (DDR2), fibroblast activation protein (FAP), fibroblast-specific protein 1/*S100* calcium-binding protein A1 (FSP1/S100A), Thyl.l and Thyl.2 (in mice) (THYI/CD90), and αSMA [13].

### 2.2. Heterogeneity

Fibroblasts in differentiated organs and anatomic sites have distinct site-specific gene expression characteristics associated with the major embryonic axis HOX gene expression patterns anterior-posterior, proximal-distal, and dermal-nondermal [56] and fibroblasts from different sites have different gene expression profiles [32,56,57,58,59]. The data are consistent with the hypothesis that fibroblasts originate from developmentally regulated MSCs, although they do not exclude the possibility that they could originate from axial epithelial or endothelial cells [29].

Investigations have demonstrated that site-specific variations in adult differentiated fibroblast gene expression programs are systematically related to their positional identities relative to major anatomic axes and maintain key features of embryonic HOX gene expression patterns that direct topographic differentiation and memory [56,57]. Large-scale differences in the population-averaged gene expression programs in cultured fibroblasts from different adult organs are related to three anatomic divisions: anterior-posterior (rostral–caudal), proximal–distal, and dermal versus nondermal [56]. These investigations conclude that fibroblasts at different locations in the body should be considered distinct differentiated cell types. This is evidenced by differentially expressed genes, including those involved in extracellular matrix synthesis, lipid metabolism, and cell signaling pathways that control proliferation, cell migration, and fate determination [57], pattern formation, cell–cell signaling and matrix remodeling, and positional identifier features of the embryonic pattern of HOX [56].

Proteomic profiles of primary human fibroblasts from human skin, lung, and bone marrow have characteristic tissue-specific differences [60]. In vitro treatment with IL-1β also results in tissue-specific responses in inflammatory proteins but uniform responses in IL-6 and IL-8 [60]. Population-averaged gene expression patterns were determined in cultured primary fibroblasts from various human organs, vocal fold, trachea, lung, abdomen, scalp, upper gingiva, and soft palate, with 6216 genes differentially expressed across all anatomic sites. Hierarchical cluster analysis revealed donor-, gender-, and age-independent global fibroblast phenotype heterogeneity based on anatomic site origin [59]. The clustering incorporated several functional themes related to transcription factors for signaling pathways regulating the pluripotency of stem cells and extracellular matrix components such as cell signaling, migration, proliferation, and differentiation potential [59].

Population-averaged transcriptional profiling of cultured fibroblasts from various anatomical sites of 13 organs revealed organ- and anatomical site-hierarchical clustering of transcripts [61]. Differential clustering was observed between gastrointestinal and non-gastrointestinal fibroblasts, submucosal layers, and subperitoneal layers within organs. The signature genes that discriminated gastrointestinal from non-gastrointestinal fibroblasts, sub-mucosal fibroblasts from sub-peritoneal fibroblasts, and fibroblasts of one organ from another organ consisted of genes associated with transcriptional regulation, signaling ligands, and extracellular matrix remodeling. Gastrointestinal fibroblasts differed from other organs’ fibroblasts by transcriptional regulation, signaling ligands, and extracellular matrix remodeling-related genes. Anatomical site clusters have different gene expression patterns, specific physiological functions, and homeostatic maintenance, and create functional diversity [61].

Intra-organ differences in population-averaged gene expression profiles were demonstrated between fibroblasts from oral lining and masticatory mucosae. Lining mucosa fibroblasts exhibited significantly higher expression of the principal structural collagens, cranial neural crest markers, and homeobox genes associated with positional memory, while masticatory mucosa fibroblasts showed greater expression of genes related to transforming growth factor-β signaling, likely associated with fibrosis, EP2 prostaglandin E2 receptor and Toll-like receptor 1 and had higher proliferation rates [58]. In the skin, fibroblast diversity embodies subpopulations with distinct functions, reflected by their lineages, and responds to wingless-related integration site (Wnt) signaling from the epidermis through direct and indirect signaling through ECM deposition and secreted factors that affect other fibroblasts in different dermal locations [62].

Investigations resoundingly concluded that the study of organ-specific and position-specific fibroblasts requires single-cell approaches. High coverage single-cell genome sequencing of normal skin fibroblasts from normal donor biopsies obtained after minimal propagation in culture demonstrated that every cell contains at least one chromosomal rearrangement and 600–13,000 base substitutions with epigenomic features resembling many cancers [63]. The study demonstrates that UV-induced and endogenous DNA damage from different parts of the body with differences in sun exposure have comparable impacts on somatic mutation loads in skin fibroblasts [63].

Single-cell transcriptomic sequencing reveals striking inter- and intra-organ heterogeneity among fibroblasts between and within different organs, primarily reflecting differences in the expression of extracellular matrix components [64]. Fibroblast subtypes localize to discrete anatomical positions and serve different physiological and regulatory functions [64]. Tissues contain distinct fibroblast types that govern distinct aspects of tissue homeostasis and disease, which include autoimmune diseases, inflammation, fibrogenic diseases, and cancer and its response to immunotherapy [65,66,67,68].

In a steady-state atlas of 28 datasets of single-cell fibroblast RNA-seq data from over 120 thousand cells from 16 unperturbed mouse tissues, investigators identified ten gene expression clusters named for the dominant cluster-specific gene, with confirmed heterogeneity within the fibroblast lineages [69]. They ascribed functional identities to eight of the clusters, including fibroblastic reticular cells (FRCs) for the chemokine (C-C motif) ligand 19^+^ (*Ccl19*^+^) cluster, red pulp fibroblasts for the Coch gene (*Coch*^+^) cluster, mesenchymal stromal and osteolineage cells for the C-X-C motif chemokine ligand 12 (*Cxcl12*^+^) cluster, intestinal fibroblasts for the fibulin (*Fbln)1^+^* and bone morphogenic protein (*(Bmp)4*^+^) clusters, fibroblasts for the cartilage oligomeric matrix protein (*Comp*^+^) cluster, alveolar fibroblasts for the nephronectin gene (*Npnt*^+^) cluster and peribronchial fibroblasts for the hedgehog-interacting protein (*Hhip*^+^) cluster. Differential enrichment core signaling pathway genes, such as nuclear factor kappa B1 (NFκB) and tumor necrosis factor (TNF) in the *Ccl19*^+^ cluster and Wnt signaling in the *Fbln1*^+^ and bone morphogenic protein (*Bmp)4*^+^ clusters suggest different functions in the clusters [69].

With respect to the two remaining clusters, phytochrome interacting factor 3-like 6 (*Pi16*^+^) and the collagen α-1(XV) chain (*Col15a1*^+^), nearly all tissues contributed to them, implying that they were universal [69]. Their analyses suggested that the *Pi16^+^* cluster passes through the *Col15a1*^+^ and ends in the specialized clusters and that genes that define these two clusters are responsible for differentiating fibroblasts [69]. Of these two universal fibroblast types, *Col15a1^+^* universal fibroblasts secrete basement membrane proteins and the *Pi16^+^ fibroblasts* serve as a potential resource to develop into distinct specialized fibroblasts, and both have stem cell phenotype characteristics [69]. *Col15a1^+^* fibroblasts are lymphocyte antigen 6 complex locus C1^−^/stem cell antigen-1^+^ (LY6C^−^SCA1^+^) and universal *Pi16^+^* fibroblasts are LY6C^+^SCA1^+^ and both can be distinguished from specialized fibroblasts, which are LY6C^−^SCA1^−^ [69]. Indeed, PDGFRα^+^ fibroblasts can be sorted into these three groups across 11 tissues [69]. These findings suggest that fibroblasts with tissue-specific features may commonly arise from universal fibroblasts [70].

Promoter-level differential expression sequencing identified 88 coding genes with higher expression in lung fibroblasts relative to fibroblasts from other sites. Key transcription factors important for lung mesenchyme development, including the T-box transcription factors TBX2, TBX4, and TBX5, are enriched in this lung-specific signature and are associated with super-enhancers. The data demonstrate that TBX4 could broadly regulate fibroblast-related pathways and is able to partly contribute to super-enhancer-mediated transcriptional programs with highly specific expression in lung fibroblasts, and is required for cell proliferation and collagen gel contraction capacity [71].

### 2.3. Isolation of Normal Fibroblasts

Fibroblasts can be isolated from various tissues for analysis. Most investigations with normal fibroblasts are conducted with skin-derived fibroblasts and isolation and characterization are delineated in Dvorankova et al. (2019) [72]. Slightly modified techniques can be applied for the isolation of normal fibroblasts from other normal excised tissue [61,73]. Various biopsy techniques can be used, but the epithelium and adipose tissue must be trimmed away [61,72,73]. For example, to obtain submucosal gastrointestinal tissue, the gastrointestinal tissue should be dissected away from the muscular layer on the luminal side to generate the lamina propria and the mucosal layer, followed by scrubbing away of the lamina propria to leave submucosal tissue [61]. Similarly, other organs can be dissected with tweezers and scissors, such as the mesentery of gastrointestinal tissue in order to obtain subperitoneal tissue, as well as other organs [61]. Due to the heterogeneity of fibroblasts from different anatomical layers of differentiated organs, it is imperative that painstaking dissection of the organ tissue layers is conducted to isolate its resident fibroblasts.

Using a generally applicable process, the dissected tissue is washed with phosphate-buffered saline (PBS) three times, incubated in PBS with 0.05% Trypsin for 4 h at 37 °C, and the solid dissected tissue is removed. The trypsin solution can be subsequently centrifuged to obtain a tissue pellet. The tissue is then minced into 1–2 cubic mm pieces [61,72,73] and cultured at 37 °C with 5% CO_2_ in Dulbecco’s Modified Eagle Medium (DMEM), 10% heat-inactivated fetal bovine serum (FBS), 100 IU/mL penicillin and 100 μg/mL streptomycin, and 250 ng/mL amphotericin B for a short time if heavy yeast contamination is of concern, such as in tissue from oral cavity tumors [72], and changed every 2–3 days [73].

To proceed to the isolation of the fibroblasts, a two-step differential detachment-attachment process can be followed [72]. After a week in culture with tissue chunks, the chunks are removed and adherent fibroblasts are detached by short periods of trypsin treatment, which enriches significantly for detached fibroblasts and leaves the more adherent keratinocytes attached to the plate. The detached fibroblasts are then reincubated in tissue culture medium and attached to plates for 20–30 min. This ensures that the vast majority attach, but leaves the remaining keratinocytes in suspension, as they take longer to attach [72]. The attached fibroblasts are washed and incubated at 37 °C in a tissue culture medium. In addition to generating primary fibroblasts de novo, many fibroblast cell lines from multiple etiologies and organs of origin are available as well from commercial sources [61,74,75,76].

Early passage of NFs does not disrupt important features of the embryonic topographic and positional identity memory patterns of HOX gene expression along major anatomic axes [56,57]. Fibroblasts collected from different anatomical sites and cultured in vitro retain their genome-wide transcriptional patterns for at least 10 population doublings [57]. The fact that global topographic differentiation is maintained in isolation with in vitro passage suggests that epigenetic mechanisms are involved in modeling and maintaining the distinctly specialized local functions and specific anatomic positions established during embryogenesis [59]. However, a ten-day co-culture of tumor cells in contact with NFs induces EMT in the tumor, with loss of E-cadherin and the characteristic tumor growth phenotypes and induction of vimentin, and acquisition of metastatic potential in vivo [77]. These data suggest that over the ten-day period, NFs are transformed into tumor-associated fibroblasts in co-culture and provide their protumorigenic EMT effects [77]. The modified reactive stroma continues to co-evolve with the cancer [78].

In contrast to early passage culture of NFs with tumor cells, cultured cancer-associated fibroblasts (CAFs) can lose expression of their typical activation markers and ligands and fail to induce EMT in cultured cancer cells [79]. When directly compared, myofibroblast markers were higher in CAFs than non-small cell lung cancer (NSCLC) NFs within five passages in the absence of continuing interaction with carcinoma cells [80].

To characterize cells as fibroblasts, they are fixed on coverslips with 4% paraformaldehyde for 5 min and stained with primary antibodies to vimentin, αSMA or phosphate buffer, and confirmed for lack of staining for cytokeratin epithelial marker, leukocyte, and macrophage markers CD45 and CD68, melanocyte markers melan A and HMB-45, and endothelial marker CD34, as controls [72,73]. They are then stained with fluorescence-labeled secondary antibodies and imaged [73]. Other methods and other control antibodies can be used, as appropriate, for different tissues. Isolated or cultured fibroblasts can be analyzed also using fluorescence-activated cell sorting (FACS). Characterizations must confirm the absence of macrophage marker CD45, epithelial markers E-cadherin and cytokeratins, endothelial markers VE-cadherin, platelet endothelial cell adhesion molecule-1 (PECAM-1), and CD31 or von Willebrand factor [81]. They must confirm the absence of pericyte markers, neuron-glial antigen-2 (NG-2), of desmin, adipocyte markers peroxisome proliferator-activated receptor (PPAR) gamma, CCAAT-enhancer binding protein (C/EBP)α [82], and uncoupling protein 1 (UCP-1) [29], in addition to the epithelial, and melanocyte markers, above.

Fibroblasts can be imaged in situ using an array of in situ sensors [83]. These include force biosensors, imaging reporters for single-cell kinase signaling, FRET-based biosensors for kinase signaling, and a variety of labeled compounds used for measuring metabolic activity, glucose uptake, intracellular adenosine triphosphate (ATP) levels, nicotinamide adenine dinucleotide (NAD)^+^, pyruvate, lactate, cell–cell interactions, and paracrine signaling to induce signaling in adjacent cells [83].

### 2.4. Normal Fibroblasts Suppress Cancer Progression

The spectrum of heterogeneous services delivered by NFs in maintaining the physiologic functions of differentiated organs, congruent with gene expression studies delineating different functional clusters [69,70], extends to their suppressive effects on epithelial cells undergoing malignant de-differentiation. The principle that the microenvironment decides that fate of a cancer has been accepted by the cancer research community for a long time [84] and is supported by extensive experimental validation [85].

Fibroblasts make up the majority of cells in the normal tissue microenvironment [86]. Their roles in the active suppression of cancer progression take on multiple forms that vary with the state of malignant progression, spanning from the early molecular and phenotypic changes in epithelial cells, to carcinoma in situ, and finally to frank, invasive, growing, angiogenic tumors with metastatic capabilities (Table 1). The first challenge that cancer cells face once transformed into carcinoma in situ is breaking through the basement membrane into the submucosal microenvironment.

Fibroblasts play a key role in the formation of the sub-epithelial/endothelial basement membrane by exporting laminins, collagen IV, and basement membrane-associated proteins [57]. Aggressive cancers lose the ability to anchor to laminin because they develop a dysfunctional, improperly glycosylated laminin-binding receptor dystroglycan or because they suppress the expression of the xylosyl and glucuronyl transferase-like protein LARGE1 [87]. These findings support the importance of laminins in the local containment of cancer. Indeed, we had previously demonstrated that the loss of laminin V expression in malignant progression from normal epithelium to ductal carcinoma of the breast occurs as a step function between the stages of ductal carcinoma in situ (DCIS) and invasive cancer [88].

Breast-specific fibroblasts and the extracellular matrix secrete products that promote the myoepithelial lineage, identified by myoepithelial-specific markers, including αSMA and cytokeratin 14 [89]. The myoepithelial cells form a protective incomplete border between breast epithelial cells and the basement membrane, separating the ductal compartment from the stromal microenvironment [90]. They act as dynamic barriers to luminal cancer cell dissemination that enlist both smooth muscle contractility and intercellular adhesion in barrier function [91,92]. They inhibit the progression of abnormally proliferating epithelial cells, including DCIS, invasive cancer, and cancer-induced angiogenesis [93] by downregulating the expression and activity of matrix metalloproteinases (MMPs) in breast cancer (BC) cells and fibroblasts [90], and vitronectin-associated cell–matrix interactions, adhesion, and invasion [94]. When DCIS transitions to invasive cancer, it penetrates the myoepithelial membrane layer and breaks down the ECM through MMPs produced by tumor cells and periductal fibroblasts [90].

Early suppression of newly transformed cells in the organ bed is inhibited by contact with fibroblasts by a process termed decades ago as neighbor suppression [95,96]. NFs variably suppress cancer cell proliferation, migration, invasion, and metastasis [97] through both non-contact and contact co-incubation in vitro [86,95,97,98,99,100]. Contact-initiated inhibition is mediated by multiple structural factors, both directly and indirectly, through the deposition of matrix proteins [101]. NFs and normal prostate epithelial cells inhibit each other’s proliferation in contact co-culture, likely by reorganizing the synthesis and distribution of the cell–cell contact-related proteoglycans decorin (DCN) and lumican (LUM) in the epithelial cells and increasing syndecan (SDC)1 at mutual intercellular contact zones [102]. However, in contrast, PC3 cancer cells do not upregulate proteoglycans in NFs but rather downregulate focal adhesion and adherens junction genes, resulting in cancer cell–cancer cell adhesion rather than cancer cell–fibroblast adhesion and autonomous cancer cell growth [102]. This exemplifies the abrogation of the restrictive roles of NFs on normal epithelial cells imposed by cancer cells through the deregulation of proteoglycans and junctional molecules and the overall disorganization and disruption of the fibroblast-malignant epithelial cell communication [102]. The suppressive effects of fibroblasts are further illustrated by observations that heparin produced by degranulating mast cells in the peritumoral fibrous tissue of human BC, head, and neck squamous cell carcinoma (SSC), NSCLC, ovarian cancer, non-Hodgkin’s lymphoma, and Hodgkin’s disease significantly restrict the colony growth of tumor cells exclusively when co-cultured with fibroblasts, an effect accentuated by FGF-7 [103].

NFs also inhibit the progression of hematologic malignancies, in addition to those of carcinomas. Dermal fibroblasts activated by contact with multiple myeloma RPMI 8226 cells induce growth arrest and alter the expression of CD38, CD45, and CD138 in myeloma cells consistent with dedifferentiation, inflammation, and progression toward dormancy [104]. In an experimental model, NFs treated with TGFβ are able to eliminate transformed fibroblasts by induction of apoptosis, suggesting another pathway in the struggle for tumor suppression by normal tissue fibroblasts [105].

NFs generate an extracellular matrix, which is softer than a CAF-generated matrix, an effect that influences cancer cell proliferation [101]. The mammary extracellular matrix has soluble and structural elements that direct differentiation toward mammary tissue. The characteristics of the matrix are sufficient to direct the differentiation of testicular-derived cells and embryonic stem cells to form functional mammary epithelial trees and normal duct outgrowth in mice [106]. The normal ductal microenvironment is also required to maintain the luminal phenotype of HR^+^ breast tumors grafted in vivo into murine mammary ducts and the suppression of SLUG expression [107]. Normal mammary fibroblasts express tumor whey acidic protein four-disulfide core domain 1 (WFDC1), which is tumor suppressive and highly expressed in resting fibroblasts but downregulated in CAFs [108]. Normal ECM also restricts cancer cell proliferation by inducing a downregulation and nuclear exit of the histone demethylase Jumonji C domain-containing (JMJD)1, resulting in growth restriction of carcinoma cells through JMJD1a-dependent modulation of gene expression [101].

Healthy intestinal NFs activate STAT1 signaling in colon cancer cells and restrain their growth, effects not imposed by CAFs or NFs treated with TNFα or by intestinal fibroblasts from patients with inflammatory bowel disease that produce high levels of TNFα [109]. Expression of RhoA in NFs exerts tumor-suppressive effects by inhibiting tumor motility and growth in vitro and in vivo, maintains tumor epithelial features, and low stiffness, and suppresses inflammation and cancer cell stemness, while, in contrast, its suppression in CAFs drives tumors [110]. Incubating PC-3 cancer cells in direct contact with fibroblast cell lines induces changes in gene expression patterns in several pathways, including Rho, the Yes-associated protein (YAP)/transcriptional coactivator with PDZ-binding motif (TAZ) cascade, pro-inflammatory signaling through NFκB, and TGFβ signaling, and the transcription factor RELA. Fibroblasts that inhibit cancer cell growth in contact co-culture exhibit differences in the Rho signal pathway and in potential individual regulators such as IL-6, MAPK8, MAP2K4, PRKCA, JUN, signal transducer and activator of transcription (STAT)3, and STAT5A compared to non-inhibitory fibroblasts [111]. YAP signaling is also a key regulator of melanoma-associated fibroblasts [112].

Dermal fibroblasts hinder melanoma tumor development when co-implanted with B16F10 cells in a β-catenin-dependent manner [113]. Normal, β-catenin-expressing fibroblasts infiltrate co-implanted tumors that form to a greater extent, permit formation of smaller tumors that proliferate less, that have lower expression of cyclin D1, and increased expression of cyclin-dependent kinase (CDK) inhibitor p16^INK4A^ [113]. Co-implanted fibroblasts also prevent tumor cell EMT, in contrast to dermal fibroblasts with genetically abrogated β-catenin expression co-implanted with tumor cells [113]. Normal stromal fibroblasts inhibit melanoma cell motility through decorin- and chondroitin-6 sulfate-induced melanocyte cell surface and cytosolic acidification that may result in increased adhesion [114].

NFs and cancer cells secrete exosomes or membrane vesicles that contain proteins and microRNAs (miRNA), establishing a cell–cell communication network within the tumor microenvironment [115,116]. NFs inhibit head and neck squamous cell carcinoma (HNSCC) tumor growth and target B-cell lymphoma-2 (BCL2) expression in vivo through the transfer of exosomes containing miR-3188, which are significantly reduced with the transition to CAFs [117]. The expression of miR-124 suppresses the invasive potential and downregulates αSMA and FAP expression in tissue fibroblasts [118]. The levels of miR-124 mRNA are significantly lower in ovarian cancer tissue than in adjacent normal tissue or normal ovaries and in ovarian cancer cell lines compared to normal ovarian surface epithelial cells [118]. miR-124 mRNA transported via exosomes from nontransformed ovarian surface epithelial cells to normal tissue fibroblasts suppresses CAF-associated phenotypes [118]. Similarly, NFs express miR-1-3 and transfer them to cancer cells via extracellular vesicles (EVs) [119]. miR-1-3 expression in BC cells inhibits viability, invasion, migration, EMT, and tumor formation and metastasis [119]. CAFs have lowered miR-1-3 levels, which may be a mechanism of loss of fibroblast-mediated suppression of BC [119]. Fibroblasts transfer miR-34a-5p in exosomes to oral squamous cell carcinoma cells and inhibit their proliferation and metastasis by binding and suppressing AXL-mediated signaling [120]. AXL tyrosine kinase promotes tumor progression through serine/threonine protein kinase (AKT)/GSK-3β/β-catenin signaling, β-catenin nuclear-translocation-induced *snail* family transcriptional repressor 1 (SNAIL) transcription, and activation of MMP-2 and MMP-9 [120]. mir-34a-5p expression is significantly decreased in CAFs, resulting in a loss of its tumor suppressive effect [120]. The expression of miR-204 in cancer-adjacent fibroblasts also inhibits tumors by suppressing fibroblast migration by modulating the expression of signaling molecules and by directly targeting integrin α11 (ITGA11) and is also downregulated in HNSSC of the oral cavity [121].

The neurogenic locus notch homolog protein 1 (Notch1) transmembrane protein family pathway negatively regulates human and mouse fibroblast motility and growth and induces apoptosis [122,123]. Quiescent fibroblasts have high levels of Notch pathway activation and expression of the pathway components, including Notch1, Notch 2, hairy and enhancer of split-1 (Hes) family BHLH transcription factor 1 (Hes-1), delta-like canonical Notch ligand 1 (Dll1), hairy/enhancer-of-split related with YRPW motif protein 1 (Hey-1), while proliferating fibroblasts have low or undetectable Notch pathway activation [123]. High levels of Notch expression in NFs are responsible for restricting melanoma cell growth in vitro and growth in the skin and angiogenesis in vivo [75,124]. Notch activation in melanoma-associated fibroblasts is lost with malignant progression [75]. Analogously, NF PTEN-to-JAGGED-1-Notch3 signaling maintains the mammary stem cell niche and inhibits BC initiation and disease progression [125].

Non-contact co-incubation of normal human foreskin fibroblasts of the cell line Hs68 (HsFb) with human lung carcinoma A549 cells suppresses cancer cell migration and metastasis mediated by TGFβ1-induced EMT, reduces E-cadherin, and elevates N-cadherin, SNAIL, vimentin, and MMP-9 via release of 5-methoxytryptophan (5-MTP) [100]. Export of 5-MTP by fibroblasts inhibits cancer cell COX-2 overexpression by blocking p300 histone acetyltransferase (HAT) and NFκB activation [126,127], inhibits A549 cancer cell migration and invasion, and retards A549 cancer growth and metastasis in a murine xenograft tumor model, possibly through COX-2 suppression [126].

IL-1β or TNFα inhibit the growth of androgen receptor (AR)^+^ LNCaP cells but not that of androgen-independent PC-3 human prostate cancer (PC) cell lines in co-cultivation with monolayers of human diploid fibroblast WI-38 cells derived from embryonic lung tissue [128]. Conditioned medium from WI-38 cells pretreated with IL-1β or TNFα is also sufficient to strongly inhibit LNCaP cell growth in an IL-6-dependent manner [128]. Normal human fibroblasts inhibit the growth of human PC cells through IL-7, induced by endoplasmic reticulum stress-mediated through IRE1alpha, ASK1, p38, and IRF-1 [129]. Endoplasmic reticulum stress is initiated by the tumor suppressor gene reduced expression in immortalized cells/Dickkopf-3 (REIC/Dkk-3 [129].

Non-neoplastic tissues adjacent to BCs have two distinct gene expression subgroups that differ in the expression of genes involved in the activation of fibrosis, cellular movement, cell adhesion, and cell–cell contact [130]. One is an active group, defined by high expression of fibrosis and cellular movement genes, and the other is an inactive group, defined by high expression of claudins and other cellular adhesion and cell–cell contact genes [130]. There is a prognostic relevance to the active gene expression extratumoral subtype, which is associated with inferior overall survival among estrogen receptor (ER)^+^ patients who were hormone-treated. There are no statistically significant associations in other patient groups [130]. The protumorigenic effects of converted fibroblasts are discussed below.

BM MSCs inhibit the proliferation but increase the invasion of oral tongue squamous cell carcinoma cells in vitro [131]. BM MSCs migrate to cancers in the circulation and become a functional part of the TME [46]. In a reciprocal relationship, tongue squamous cell carcinoma cells upregulate the expression of inflammatory chemokines by BM MSCs, whereas BM MSC products induce the expression of known invasion-linked molecules by carcinoma cells and enhance invasion and type I collagen mRNA synthesis. This is functionally significant because high expression of type I collagen N-terminal propeptide (PINP) in vivo correlates with the cancer-specific mortality of tongue squamous cell carcinoma patients [131]. The cancer-inhibitory effects of proliferating normal tissue fibroblasts are comprehensively reviewed by Delinassios and Hoffman (2022) [132].

BM MSCs suppress the proliferation of metastatic BC cells in the bone marrow, decrease cell surface expression of stem cell markers, decrease sensitivity to docetaxel, and inhibit proliferation and invasion. These effects are transmitted by exosomes from BM MSC cultures that include miRNA-23b, which suppresses MARCKS, a cell cycle and motility-inducing gene among others [133]. BM MSCs also induce dormancy in metastatic BC cells [134] through FGF-2-mediated inhibition of cell cycle progression through TGFβ1 [135], activation of cyclin-dependent kinase inhibitors p21^Waf1^ [136], p15^INK4b^ [135], and p27^Kip1^ [135], inhibit motility through stromal FGF-2- and integrin α5β1-mediated RhoA inhibition and actin-rearrangement in the cancer cells [137,138] and induce cytotoxic therapy resistance through phophatidylinositol-3 kinase (PI3K)/AKT [134,139].

**Table 1:** Normal fibroblasts suppress cancer cell malignant behavior.

Mechanism	References
Neighbor suppression	[95,96]
Induce apoptosis in transformed fibroblasts	[105]
Lay down basement membrane	[57]
Anchor to laminin	[87]
Contact inhibition; reorganize synthesis and distribution of DCN, LUM, SDC1	[102]
Induce dormancy-like state in myeloma cells	[104]
Maintain epithelial phenotype by suppressing SLUG	[107]
Suppress cancer progression through WFDC1	[108]
Suppress cancer cell proliferation by suppressing nuclear exit of the histone demethylase JMJD1	[101]
Activate STAT1 in cancer cells	[109]
RhoA expression in NFs; inhibits tumor motility and growth, maintains epithelial features, cancer cell stemness, maintains low ECM stiffness and suppresses inflammation	[110,111]
YAP signaling	[111,112]
NF expression of IL-6, MAPK8, MAP2K4, PRKCA, JUN, STAT3, and STAT5A induced by cancer cell confrontation	[111]
NF expression of β-catenin inhibits tumor proliferation, decreases cyclin D1, increases p16^INK4A^, and prevents tumor cell EMT	[113]
Secrete exosomes with miRNAs	
Suppress MARCKS, cell cycle and motility, tumor growth (miRNA-23b)	[133]
Inhibit tumor growth and Bcl2 (miR-3188)	[117]
Suppress invasion, downregulate αSMA and FAP (miR-124)	[118]
Inhibit viability, invasion, migration, EMT, tumor formation and metastasis (miR-1-3)	[119]
Suppress proliferation and metastasis by suppressing AXL signaling (miR-34a-5p)	[120]
Inhibit fibroblast migration by targeting ITGA11 (miR-204)	[121]
Notch 1 and 2, Hes-1, Dll1, Hey-1, PTEN, Jagged maintain NF quiescence, restrict cancer cells, maintain stem cell niche	[75,123,124,125]
NF 5-MTP	
TGFβ1-induced EMT, reduction of E-cadherin and elevation of N-cadherin, SNAIL, vimentin and MMP-9 in cancer cells	[100]
COX-2 overexpression in cancer cells by blocking p300 histone acetyltransferase and NFκB activation, migration, invasion, growth, and metastasis	[126,127]
FGF-2-mediated cell cycle inhibition and dormancy through TGFβ1, p21^Waf1^, p15^INK4b^, p27^Kip1^, motility inhibition through FGF-2- and integrin α5β1-mediated RhoA inhibition and actin-rearrangement in cancer cells, treatment resistance through PI3K/AKT	[134,135,136,137,138,139]
IL-1β or TNFα treated NFs inhibit cancer cell growth	[128]
NF REIC/Dkk-3-induced IL-7 inhibits tumor growth	[129]

## 3. Cancer-Associated Fibroblasts

### 3.1. Origin of CAFs

The tumor microenvironment consists of cells originally present in the normal tissue, including fibroblasts, pericytes, resident MSCs, adipocytes, endothelial cells, epithelial cells, immune cells, as well as cells recruited to the tumor, including hematopoietic stem cells, which are converted to CAFs [46,140,141]. CAFs become the most common constituents of the tumor stroma and take on roles as agents of cancer progression [142].

Initially, the predominant sources of cancer-associated MSCs are local MSCs, which are reprogrammed early upon tumor formation by direct contact, transfer of exosomes or fusion with cancer cells, or by interactions with other microenvironmental cells [143,144,145]. Later, as the microenvironment becomes more inflammatory [146], tumors recruit exogenous MSCs by secreting chemokines such as CC-chemokine ligand 2 (CCL2), CCL5, CXCL12, and CXCL16 [147,148,149]. BM-derived MSCs are recruited to breast and lung tumors, differentiate to a distinct subpopulation of CAFs that lack PDGFRα, promote tumor growth and angiogenesis via expression of Clusterin, and end up as a diminished PDGFRα-expressing resident CAFs population [150]. The recruited BM MSCs have a negative impact on patient survival, evidenced by the fact that decreased PDGFRα expression in BC patients is associated with a worse prognosis [150].

MSCs represent 0.1–5% of the tumor stroma in a variety of cancers [46,151,152]. No single marker exists to identify the extremely heterogeneous MSC populations and their functions [153], although they continue to fit the minimum criteria outlined by the International Society for Cellular Therapy for BM MSCs [42,46]. Algorithms have been developed to characterize this heterogeneous population by differences in transcription profiles between cancer-associated and normal MSCs [145]. MSCs actively influence tumor progression and metastasis [143,144] by directly interacting with tumor cells and other microenvironmental components [154]. Cancer cells, such as BC cells, acquire gene expression signatures enriched in survival factors, tumor suppressors, and inflammatory mediators, defining a state of secretory senescence [155]. MSCs also modulate the cancer immune microenvironment. They suppress innate and adaptive immune systems, including suppressing of T-cells through prostaglandin E2, CXCL9, CXCL10, and CXCL11, and nitric oxide synthase [156] in response to cancer cell-secreted TNFα, IFNγ, and IL-1 [46]. They induce the differentiation of immunosuppressive M2-polarized macrophages by cancer-secreted exosomes and decrease the cytotoxic activity of natural killer (NK) cells [157]. Cancer-associated MSCs suppress the response to immune checkpoint inhibitor therapy in ovarian cancer through the expression of CCL2, C-X3-C motif chemokine ligand 1 (CX3CL1), TGFβ1, recruitment of C-C motif chemokine receptor (CCR)2^+^ monocytes and M2 tumor-associated macrophages [158].

Direct tumor cell contact co-culture of MSCs and hypoxia are necessary to fully convert MSC to cancer-associated MSCs [145], and in fact, high oxygen tension inhibits CAF protumorigenic effects [159]. Studies have suggested that tumor-associated MSC characteristics do not arise through the acquisition of genetic mutations but rather, through epigenomic reprogramming by cancer cells that induce them to assume a strongly protumorigenic phenotype [46,152]. Cancer-associated MSCs have different gene expression profiles from those of normal tissue MSCs [145,160]. Once MSCs convert to cancer-associated MSCs, they, in turn, may have the potential to convert endemic MSCs into cancer-associated MSCs as well [161]. Tissue MSCs and BM MSCs recruited by the cancer are also epigenetically reprogrammed by cancer cells to become their tools to support tumor progression and to differentiate into multiple lineages, including to CAFs, myofibroblasts, and adipocytes, and assume significant roles in the formation of the TME [46]. Human BM MSCs incubated for 30 days with tumor-conditioned medium from ER^−^ MDA-MB-231 BC cells, U87 glioma cells, and PANC-1 pancreatic carcinoma cells assume a CAF-like myofibroblast phenotype [162]. They acquire sustained expression of SDF-1, αSMA, FSP, and the ability to promote tumor cell growth in vitro and in vivo [162].

Co-incubation of BM MSCs with cancer cells increases the expression of genes associated with tumor growth, metastasis and angiogenesis, and functionally increases tumor cell resistance to carboplatin [161]. The secretome of cancer cells [163] and soluble agents such TNFα contribute to resistance to treatments [143,161]. MSCs also induce treatment resistance in cancer cells through Src activation, enhancing antioxidant defenses, induction of quiescence, and tumor-initiating profiles [164,165]. This is congruent with the EMT-inducing effects of MSCs on cancer cells [155].

The top 10 pathways enriched by a 30-day incubation in cancer conditioned medium are MAP kinase, focal adhesion, cell cycle, regulation of actin cytoskeleton, cytokine–cytokine receptor, Wnt signaling, axon guidance, apoptosis, insulin signaling, and gap junctions [162]. Their gene expression patterns are similar to CAFs exposed to cancer-conditioned medium-treated CAFs [162]. MSCs co-cultured with colorectal cancer (CRC) cells upregulate many factors, of which IL-8 is the most highly upregulated angiogenesis factor, through which they induce human umbilical vein endothelial cell (HUVEC) proliferation and migration, tube-formation, and CRC cell proliferation in vitro and angiogenesis in vivo [166]. Tumor-associated MSCs induce angiogenesis through secretion of TNFα, vascular endothelial growth factor (VEGF), interferon (INF)γ, leukemia inhibitory factor (LIF), macrophage colony-stimulating factor (M-CSF) and endothelin secretion by tumor cells [46]. Parathyroid adenomas also induce neoangiogenesis by stimulating MSC to express FAP and VEGFA mRNA in transwell co-culture with hBM MSCs through CXCL12/CXCR4 [167]. While the hypoxic tumor microenvironment generates abnormal new blood vessels through both tumor-induced and CAF-induced factors, it also is a necessary factor for the conversion of NFs to CAFs, mediated through HIF1α-induced NFκB, CCL5, COL1A2, and αSMA [168]. Hypoxic CAFs contribute to blood vessel abnormalities, which lead to dysfunctional tumor blood vessels [169]. In vitro co-culture of hypoxic mammary CAFs with endothelial cells promotes angiogenesis through the hypoxia-induced remodeling of the CAF proteome, including upregulated expression of novel proteins, most prominently NAV2 Antisense RNA 2 (NCVP2-AS2) (renamed hypoxia-induced angiogenesis regulator (HIAR)), which is necessary for the pro-angiogenic and pro-migratory effects of hypoxic CAFs, mediated through VEGF/VEGFR signaling [169].

However, besides the tumor-promoting effects of MSCs, they can also suppress tumors [170]. Some have proposed that the source and gene expression heterogeneity of MSCs from different organs directs these divergent effects [46]. When stimulated with tumor necrosis factor (TNF)α, human BM MSCs express TNF-related apoptosis-inducing ligand (TRAIL), induce apoptosis, and inhibit in vivo tumor formation in triple-negative BC cells [170]. In turn, apoptotic triple-negative BC cells and the DNA fragments they generate further upregulate TRAIL in a toll-like receptor 3 (TLR3)- [170] and an absent in melanoma 2 (AIM2)-dependent [171] manner, further increasing BC cell apoptosis in a positive feedback loop. In addition, activated MSCs potentiate the effects of a subtoxic dose of doxorubicin synergistically in vitro [170] and on metastases in an in vivo model [171].

Further evidence of this dualism comes from studies that demonstrate that endemic MSCs and those recruited to the tumor microenvironment have both protumorigenic and anti-tumor effects [23,172]. Hybrid cell formation occurs frequently by fusion between cancer cells and various other cell types within the TME, including frequently with MSCs [23]. The resulting hybrid populations are aneuploid, a characteristic associated with chromosomal instability. Fused cancer cells can acquire a slew of new functionalities, and can undergo apoptosis, necrosis, senescence, dormancy, or enhanced proliferative or metastatic capacity. In addition, they engender new therapeutic responsiveness or resistance, based on enhanced tumor plasticity [23]. Triple-negative human BC cells fused with BM MSCs remain quiescent in a dormant state in vivo in immunodeficient mice for up to six months, with the expression of dormancy-associated transcripts, then they awaken and grow rapidly into metastatic and heterogeneous tumors with variable chemosensitivity [173]. MSCs recruited to the tumor stroma surround the BC cells in three-dimensional environments and under conditions of low nutrient availability, promote the spheroid formation, and are then cannibalized by the cancer cells in a Rho kinase-dependent manner [155]. The process suppresses tumorigenicity and promotes dormancy in the cancer cells with characteristic significant increases in the expression of EMT program genes and stem cell markers (SCMs) [155].

### 3.2. Cancer Cells Convert NFs into CAFs

#### 3.2.1. Reciprocal Effects of Cancer Cells and Stromal Fibroblasts

The tumor microenvironment is a chronic site of inflammation [174]. Cancer cells release pro-inflammatory cytokines and chemokines that attract immune cells [175]. The inflamed tumor microenvironment also attracts bone marrow MSCs, which are home to the primary tumor and contribute to the attempt to repair the nonhealing wound that is the tumor [20,176,177]. In turn, they release cytokines that instead, promote malignant behavior in the tumor [175,176]. This cytokine-rich milieu stimulates tumor growth and invasion [177]. The reverse direction of distant control also occurs concurrently. An example is the case of pancreatic stellate cells, which induce fibrinogenesis and stimulate angiogenesis in the primary tumor [178] by depositing a periostin-rich matrix around capillaries and secreting vascular endothelial growth factor (VEGF) [179]. Pancreatic cancer has a significant reduction of microvessel density, and the resultant hypoxia increases stellate cells and their secretion of periostin and collagen I, fibronectin, and vascular endothelial growth factor (VEGF), while, in turn, stellate cells increase endostin production by cancer cells by MMP-dependent cleavage [179]. Stellate cells leave the tumor, circulate to distant sites, and facilitate seeding, survival, and proliferation in metastatic sites [180]. Table 2 outlines the factors contributing to the reciprocal feed-forward relationship between cancer cells, the TME, and microenvironmental fibroblasts.

Tumor cells remodel their microenvironment and enhance tumor progression and chemoresistance in multiple ways [181]. Cancer cells release growth factors, such as FGF-2, VEGF, PDGF, EGF, TGFβ, and cytokines and chemokines, the core group consisting of CCL2, CCL5, IL-6, IL-8, as well as other soluble factors that activate fibroblasts [182,183,184] and modulate CAF gene expression patterns [185,186] and their metabolism [187,188]. These factors promote the proliferation, motility, and survival of epithelial cancer cells, but not that of dormant tumor-initiating cells [189,190]. Other mechanisms of control of stromal behavior by cancer cells include the expression of adhesion molecules. One example is CD147 transmembrane glycoprotein overexpressed on BC cell surfaces, which promotes the transformation of fibroblasts to CAFs, which in turn induces EMT in BC cells [191].

The proximity of cancer cells to adipocytes in the breast microenvironment induces the adipocytes to transform into adipocyte-derived fibroblasts (ADFs) [192]. These adipocyte-derived MSCs respond to Wnt3a secretion by tumor cells by reactivating Wnt/β-catenin, increasing expression of the marker FSP1, but not αSMA, increasing fibronectin and collagen I secretion, and enhancing cancer cells’ migratory and invasive capabilities [192]. Adipocyte-derived MSCs can be differentiated to osteoblasts by expression of FGF-2 [193,194] and BMP-2 [194], and to adipogenic, chondrogenic, and osteogenic lineages by medium additives endothelial growth medium and FGF-2 [195]. Obese subcutaneous adipose tissue and visceral adipose-derived stromal fibroblasts (ADFs) also promote the growth and dissemination of ovarian epithelial cells co-injected intra-peritoneally but do so more effectively than lean mouse subcutaneous adipose tissue [196]. Obese ADFs attract CD3^+^ T-lymphocytes and F4/80^+^ macrophages and increase the expression of chemotactic factors IL-6, MIP-2, and MCP-1 when cultured with tumor cells [196]. However, they differentiate into adipocytes and osteocytes less readily than ADFs from subcutaneous fat from lean mice [196]. Adipocytes promote the growth of other malignancies as well, including multiple myeloma cells in bone [197].

Genetic changes in primary tumor cells induce uniform and permanent genetic changes and tumor-enhancing properties in stromal fibroblasts [198,199] and in bone marrow stromal cells in co-culture [200]. As a consequence, the distribution of mammary stromal cell type changes in cancer. The frequency of CD34^+^ fibroblasts, found in normal breasts, markedly decreases, and the frequency of cancer-associated αSMA^+^ fibroblasts increases [201]. These changes are associated with a higher stage and worse prognosis [201]. CAFs have higher levels of RhoA and Rac1 than mammary fibroblasts not associated with tumors [202]. Co-culture of bone marrow stroma with PC cells endows permanent cytogenetic changes in the stromal cells and an ability to promote the growth of PC in mice [200]. With reactive oxygen species (ROS) acting as mediators, PC cells induce the expression of extracellular matrix (versican and tenascin) and chemokine (BDFN, CCL5, CXCL5, and CXCL16) genes, responsible for the induced growth in vivo of PC [200]. These observations collectively suggest coevolution of cancer and stromal cells occurs under three-dimensional growth conditions, which ultimately accelerates cancer growth and metastasis [200]. When significant cancer cell numbers come in contact with stroma, they impose modified behavior. In our own model 2D co-culture, MCF-7 cells at non-clonogenic density, induced a cell number-dependent induction of IL-6 and IL-8 when co-cultivated with murine bone marrow stroma, generating a positive feedback loop for their growth stimulation [138].

Cancer cells also induce tumor-enhancing properties in MSCs from breast adipose tissue [203]. Adipose tissue MSCs can contribute to pericytes and adipocytes that populate the tumor microenvironment [204]. In addition, cancer cells have been shown to “educate” bone marrow-derived MSCs that home to tumors. In direct co-incubation experiments or through incubation in tumor conditioned medium, bone marrow MSCs increase the secretion of osteopontin (OPN), IL-8, and FGF-2, increasing their ability to act as chemoattractants to cancer cells, while decreasing vimentin and αSMA expression, which in vivo would act to keep them in the microenvironment by decreasing their migration [175].

The features of the primary tumor microenvironment favor the influx and conversion of supporting cells to CAFs. These factors include low pH, hypoxia, glycolytic oxidation, stiffness, inflammation, ionic gradients, and high concentrations of chemokines and cytokines [205]. These factors favor tumor growth and progression and foster the conversion of NFs and other local or recruited cells, outlined above, to CAFs, as well as the preparation of the pre-metastatic niche [206]. Nascent and growing cancer cells initiate the conversion of NFs, MSCs, and other supporting cells to CAFs. The process occurs through soluble factors secreted by the cancer cells, by direct contact with fibroblasts and MSCs, by the export of exosomes, and by direct fusion with fibroblasts and MSCs. These processes, the transfer of exosomes containing proteins and miRNAs, and cell fusion occur multi-directionally between cancer cells and CAFs, and other cells in the microenvironment. This section addresses the conversion of NFs, MSCs, and other cells to CAFs. The reciprocal effects of CAFs on cancer cells are discussed in the later sections below.

Replicative senescence endows fibroblasts with a tumor-supportive ability by direct mitogenic effects and through an MMP-mediated increase in the permeability of adjacent capillaries, which permits exposure of cancer cells to increased levels of mitogens, cytokines and other plasma products [207]. Chemotherapy, biotherapy with CDK inhibitors and radiation induce a secretory senescence phenotype through DNA damage response mediated by ATM and NFκB activation, characterized by secretion of inflammatory cytokines IL-6 and IL-8 [208,209,210]. However, the effects are dependent on chromatin remodeling rather than physical breaks in DNA, evidenced by osteopontin activation form HADC inhibitor treatment [211]. Dietary fat and IL-1β may also provide an environment that promotes NF transition to secretory senescence phenotypes [212]. Aging induces senescence in MSCs through loss of expression of FOXP1 [213]. Secretory senescence promotes tumor progression, immunosuppression, treatment resistance, and recurrence of dormant cells [138,214].

**Table 2:** Reciprocal effects of cancer cells and stromal fibroblasts.

Actions by Cancer Cells	Effects on Fibroblasts	Refs.	Actions by Fibroblasts	Effects on Cancer Cells	Refs.
TME has low pH, hypoxia, glycolytic oxidation, stiffness, inflammation, ionic gradients and high concentrations of chemokines and cytokines	- Environment fosters NF and other local or recruited cell conversions to CAFs - Cancer cells act through soluble factors, direct contact, export of exosomes, direct fusion with MSCs and fibroblasts	[205,206]	Converted CAFs	- Favor tumor growth and progression - Preparation of the pre-metastatic niche	[205,206]
Tumor hypoxia from low vessel count	Stimulate stellate cell periostin, collagen I, fibronectin, VEGF secretion	[179]	- Pancreatic stellate cells induce fibrinogenesis, deposit periostin-rich matrix around capillaries, secrete VEGF - Tumor residing stellate cells circulate to distant sites	- Stimulate angiogenesis in the primary tumor. Induce endostin production by cancer cells - Facilitate tumor seeding, survival and proliferation in metastatic sites	- [178,179] - [180]
Release pro-inflammatory cytokines	Attract BM MSCs to the tumor	[20,175,176,177]	Newly arrived MSCs release cytokines	Released cytokines promote malignant behavior in the tumor	[175,176,177]
Cancer cells release FGF-2, VEGF, PDGF, EGF, TGFβ, CCL2, CCL5, IL-6, IL-8,	- Activate fibroblasts - Modulate CAF gene expression - Modulate CAF metabolism	- [182,183,184] - [185,186] - [187,188]	Activated fibroblasts secrete cytokines	Promote proliferation, motility, and survival of epithelial cancer cells but not dormant tumor initiating cells	[189,190]
Cancer cell co-cultivation or conditioned medium “educate” BM MSCs that home to tumors	BM MSCs increase OPN, IL-8, FGF-2 secretion, decrease vimentin, αSMA expression	[175]	OPN, IL-8, FGF-2 attract cancer cells; vimentin, αSMA decrease MSC migration, keep them in the TME	Promote cancer progression	[175]
BC cells overexpress CD147 transmembrane glycoprotein.	CD147 promotes transformation of fibroblasts to CAFs	[191]	CD147-transformed fibroblasts express αSMA	Induce EMT in co-cultured BC cells	[191]
Cancer cells induce ratio-dependent secretory senescence in co-cultivated BM stroma	BC cells ratio-dependent stromal secretion of IL-6 and IL-8	[138]	Stroma incubated with cancer cells secrete cytokines	Cytokines induce positive feedback loop for cancer cells growth stimulation	[138]
Breast cancer cells transform nearby adipocytes into adipocyte-derived fibroblasts (ADFs) through secretion of Wnt3a	ADFs reactivate Wnt/β-catenin, express FSP-1, are more migratory/invasive	[192]	ADFs secrete fibronectin and collagen I	Increase invasion in co-cultivated tumor cells	[192]
Tumor cells co-cultured with obese animal and visceral ADFs induce inflammation	Increase IL-6, MIP-2 and MCP-1 expression in obese and visceral ADFs	[196]	Tumor-stimulated ADFs attract CD3^+^ T-lymphocytes and F4/80^+^ macrophages	Promote growth and dissemination of ovarian epithelial cells in vivo	[196]
Breast cancer cells modify adjacent adipose tissue MSCs	Cancer-adjacent MSCs upregulate BDNF, NOTCH1, SOX9, vimentin, VCAM1, downregulate growth differentiation factor 15 (GDF15), IGF1, MMP2, PDGFRβ, TGFβ3, BMP4 and have increased proliferative potential	[203]	- Cancer associated adipose MSCs enhance BC cell aggressiveness - Contribute to pericytes and adipocytes populating the TME	Enhanced tumorigenicity, collective cell invasion, EMT^+^ invasive front tumor cells, adjacent nerve invasion in xenografts	[203,204]
Genetic changes in primary tumor cells induce genetic and gene expression changes in stromal fibroblasts through ROS.	- Fibroblasts increase ECM proteins and chemokines	- [198,199,200]	- Tumor-induced genetically modified stromal and BM fibroblasts increase versican, tenascin, BDFN, CCL5, CXCL5, and CXCL16 - Frequency of normal breast CD34^+^ fibroblasts, markedly decreases, and that of αSMA^+^ fibroblasts increases - CAFs have higher levels of RhoA and Rac1 than NFs	- Promote tumor progression - Associated with higher stage and lower overall and disease-free survival - Active in invasion of cancer cells	[198,199,200] - [201] - [202]

#### 3.2.2. Soluble Factors

CAFs contribute to tumor progression by releasing cytokines, growth factors, and hormones [215]). Cancer-derived factors induce fibroblast clustering and production of HGF/scatter factor, which, in turn, enhances the invasiveness of c-Met-expressing carcinoma and leukemia cells through IL1, -6, -8, and 11, leukemia inhibitor factor, and granulocyte-macrophage colony-stimulating factor in a process termed nemosis [216,217]. The process in leukemia requires c-Met expression [216]. Nemosis results in increased production of growth factors, pro-inflammatory cytokines, proteolytic enzymes, and degradation of cytoskeletal proteins [218]. Activated CAFs have increased mitotic indices, mutations in tumor suppressor genes such as p53, and increased secretion of growth factors, chemokines, and ECM components, all enabling invasion and tumor growth [219]. Nemosis variably upregulates the expression of cyclooxygenase-2 (COX-2) and CAF markers αSMA, FSP1 (S100A4), and fibroblast activation protein FAP [218]. Four subpopulations of CAFs have been identified to reside variably in breast tissue [220]. Variable BC subtypes, luminal, Her2^+^, and triple-negative can be classified by differential expression of FAP, SMA, FSP1, PDGFRβ, CD29, and CAV1, with the expression of FAP identifying subpopulations that modulate tumor growth, cancer cell proliferation, extracellular matrix remodeling, metastatic dissemination, immunosuppression, and resistance to treatment [220]. Fluorescent probes [221] and ^68^Ga-FAPi-46 PET [222] have been developed to image FAP distributions in tumors in vivo in preclinical studies and in patients, respectively.

High stromal expression of transcription factor paired related homeobox 1 (Prrx1) in pancreatic cancers is associated with a more aggressive, squamous subtype of the cancer than cancers in other patients [223]. CAFs co-cultured with tumor organoids induce EMT and confer gemcitabine resistance to pancreatic cancer cells through hepatocyte growth factor (HGF) [223].

Hyaluronic acid (HA) in tumor stroma promotes tumor invasion and progression. Co-culture of Panc-1 cells and stromal fibroblasts markedly increases cancer cell migration, an effect dependent on increased hyaluronan synthases *HAS3* mRNA expression and HA synthesis [224]. HtrA serine peptidase 1 (HTRA1), which is involved in malignant transformation of several cell types, is positively correlated with αSMA expression in gastric cancer tissue and induces the expression of αSMA in NFs through NFκB-mediated FGF-2 expression and export [225].

TGF-beta is the most accepted factor driving the transformation of tissue fibroblasts to CAFs [226]. PCs recruit BM-MSCs, which in turn, are able to convert NFs via secreted TGFβ1 to CAFs, which enhance PC growth and invasion [227]. The expression of galectin-1 (Gal1), a functionally polyvalent carbohydrate-binding protein with an affinity for β-galactosides and wide-ranging biological activity and expression in various normal and pathological tissues, is positively correlated with TGF-beta expression in epithelial cells in gastric cancer patients [226]. Conditioned medium from gastric cancer cells induces the expression of both Gal1 and αSMA in NFs via TGFβ secretion [226]. Gal1 is highly upregulated in the CAFs of multiple human cancers, promotes tumor progression, and is required for TGFβ-induced conversion of NFs to CAFs. In turn, conditioned medium from fibroblasts overexpressing Gal1 inhibits cancer cell apoptosis and promotes migration and invasion in vitro [226]. Similarly, Gal3 in pancreatic cancer cells induces proliferation and invasion in pancreatic stellate cells, stimulates transcription of IL-8 through integrin subunit beta (ITGB)1, and enables stellate cells to support tumor growth and metastases in orthotopic tumors when co-implanted in mice [228].

TGFβ is also the most potent suppressor of immune system activity against cancer cells in the TME, with effects mediated through stimulation of CD4^+^ regulatory T-cells (Tregs), suppression of cytotoxic CD8^+^ T-lymphocytes (CTLs) and natural killer (NK) cells, the polarization of macrophages to M2 cells, infiltration and differentiation of fibroblasts into CAFs [229]. TGF-beta also plays a key role in angiogenesis, invasion, and DNA damage responses in cancer cells.

Cancer cells enhance the tumorigenic potential of fibroblasts by secreting pro-inflammatory cytokines, such as LIF and IL-6 to mediate the epigenetic modification of CAFs, which, in turn, secrete LIF and IL-6 and form a feedback loop to enhance actomyosin contractility and ECM remodeling to form the tracks used for collective cancer cell migration [230]. The highly expressed secretory protein tumor necrosis factor alpha stimulates gene 6 (TSG-6) has an essential role in cancer cell-induced inflammatory response and ECM remodeling and is associated with poor prognosis and metastasis [231]. TSG-6 secreted by CRC cells activates Janus kinase (JAK)2-STAT3 and NF reprogramming into cancer-associated fibroblasts with upregulation of pro-metastatic cytokines CCL5 and MMP-3, higher motility in vitro, and increased support of cancer metastasis in vivo by TSG-6–expressing fibroblasts [231]. CAF-secreted IL-17α markedly enhances the migration and invasion of gastric carcinoma cell line AGS and SGC-7901 cells, effects mediated in co-culture by increased levels of MMP2/9, reduced expression of TIMP1/2, and activation of JAK2/STAT3 signaling [215]. The number of αSMA^+^ CAFs in gastric cancer tumor tissue from 227 patient tumor samples positively correlated with advanced TNM stage, and perineural invasion, and was an independent risk factor for worse disease-free survival (DFS) and disease-specific survival (DSS) by multivariate analysis [215]. Prolonged pro-inflammatory stimulation in the tumor microenvironment induces a deficiency in base excision repair in normal tissue fibroblasts, generating unrepaired DNA strand breaks, and triggering an ATF4-dependent reprogramming of NFs into CAFs [232].

Wild-type p53 in NFs modulates the spectrum of secreted proteins to render their microenvironment suppressive of adjacent tumor cells. In turn, cancer cells parry to suppress p53 induction in surrounding fibroblasts through secreted factors transmitted by CM [233,234]. Transcriptional programs and secretomes supported by p53 are altered substantially in CAFs or NFs co-incubated with cancer cells, contributing to the altered, tumor-promoting features in vitro and in vivo [235]. This renders p53 a significant contributor to the intrinsic features of CAFs, including the promotion of cancer migration and invasion through non-mutational modification of its activities, such as the previously noted epigenetic modifications and differential expression of miRNAs [235]. Fibroblasts with ablated or mutant p53 promote the growth of PC3 prostate cancer cells in vitro in an SDF-1-dependent manner and significantly enhance the metastatic spread of PC [236] and other cancer cell types in vivo [234]. In fact, because fibroblasts with defective p53 undergo senescence at much lower rates than NFs, there is a selection for mutant p53- or p53-deficient fibroblasts that promote tumor progression in the TME in vivo [237], suggesting that tumor progression may involve a process of non-mutational modification of p53, switching its effects from tumor-suppressive to tumor-enhancing transcriptional programs [235].

Chemotherapy with doxorubicin has the unintended effect of inducing senescence in NFs, eliminating their protective, anticancer effects [238]. However, pre-treatment with Quercetin can reduce the number of senescent cells and the production of senescence-associated secretory phenotype (SASP) factors and decreases the protumor effects of conditioned medium from doxorubicin-induced senescent fibroblasts on osteosarcoma cells [238]. Similarly, radiation of normal lung fibroblasts induces the stabilization of TRAF4-mediated phosphorylated cytosolic protein p47-phox-p22phox (NOX) complex, decreases lysosome degradation, induces higher endosomal ROS levels and NFκB-mediated ICAM1 upregulation and secretion, leading to enhanced NSCLC proliferation and EMT in vitro and in vivo [239].

PC significantly increases the expression of YAP1 in the normal stromal cell, converting them to CAFs through the YAP1/TEAD1 protein complex, which regulates SRC transcription, affects downstream cytoskeletal proteins, and converts NFs to CAFs [240]. The CAFs, in turn, enhance the proliferation, growth, invasion, and metastasis of tumor epithelial cells [240]. The relevance of this mechanism is supported by the detection of high expression levels of YAP1 in the tumor stromal cells of PC patients with advanced tumor stage and poor prognosis [240].

BC cells trigger stromal Notch-Myc and drive RNA polymerase III (PolIII)-induced increases in RNA components of signal recognition particle (RN7)SL1, an endogenous RNA normally shielded by RNA binding protein SRP9/14 [241]. RN7SL1 hence becomes unshielded in stromal exosomes, and upon transfer to immune cells and BC cells, drives an inflammatory response and activates damage-associated molecular patterns (DAMPs) pattern recognition receptor (PRR) retinoic acid-inducible gene I (RIG-I) to enhance tumor growth, metastasis, and therapy resistance, respectively [241].

#### 3.2.3. Exosomes

Tumor cells, including tumor-initiating cells [242], can remodel their microenvironment in the primary tumor setting and in metastatic sites to enhance tumor progression and chemoresistance through the secretion of exosomes [133,243,244,245,246]. Exosomes either fuse with or interact with other cells, and their content molecules mediate specific cell-to-cell interactions and activate signaling pathways in the cells with which they interact [247]. Since exosomes are secreted by normal, metaplastic as well as transformed cells, they can act as universal tools for either preventing tumor progression, as in the case with NFs, or in converting fibroblasts to CAFs and in turn, conferring the protumorigenic effects and drug resistance endowed by CAFs [247]. Exosomes also have the ability to induce oxidative reprogramming by restoring lost respiration in cancer cells and suppressing tumor growth [184].

Acidic and hypoxic microenvironments generate exosomes with characteristic CAF miRNAs that promote proliferation, immune suppression, EMT, drug resistance, and metastasis [248]. Exosomes are a subset of secreted EVs with lipid bilayer membranes and diameter in the range of 20–100 nm, while microvesicles, another subset of EVs, have a diameter range of 100–1000 nm [184,247,249,250]. Exosomes are shed by most cell types and are present in many body fluids, including blood, urine, saliva, and breast milk [250]. They can contain coding and non-coding RNAs, circular RNAs (circRNAs), ssDNA and dsDNA, lipids, small molecules, proteins, and growth factors, all of which can be taken up by nearby or distant cells [242,246,250,251]. In cancer, they can reach distant sites through circulation and act as likely envoys with roles in establishing the metastatic niche [242,246,251].

Exosomal RNAs are heterogeneous in size but are enriched in small RNAs, such as miRNAs, which are able to exert genome-wide regulation of gene expression [247]. miRNAs are short, non-coding RNAs involved in post-transcriptional gene regulation that negatively modulate gene expression [252], are important regulators of tumor invasion, proliferation, and colony formation [253] and are regarded as agents acting as tumor messengers to “corrupt” stromal cells [254]. In addition to transferring miRNAs, tumor cells also transfer exosomal circular RNAs, which are long non-coding stable, degradation-resistant RNA molecules with closed loop structures lacking a 5′ cap and 3′ tail. Exosomal circRNAs affect malignant progression through signal transduction, promote tumor metastasis by regulating gene expression, RNA transcription, and protein translation and facilitate metastasis by altering the tumor microenvironment and the pre-metastatic niche [250]. While a great deal of the literature describes the transfer of miRNAs from cancer cells to fibroblasts and vice versa via exosomes, naked nonvesicular extracellular mRNAs (nex-mRNAs) are taken up by cells and exert effects as well [255]. In one example, human NK cells take up an IL-1β nex-mRNA, which exerts a translation-independent function [255]. The IL-1β nex-mRNA 3′UTR binds RNA-binding zinc finger CCCH domain-containing protein 12D (ZC3H12D) at the cell membrane and transports it to the nucleus [255]. There, it enables NK cell survival by upregulating antiapoptotic gene expression, migration activity, and interferon-gamma production, leading to NK cell killing of cancer cells and antimetastatic effects in mice [255]. Not all miRNAs are tumor-promoting, however. As noted below and in the section on NFs discussed earlier, some miRNAs have cancer-inhibitory effects and play functional roles in the struggle by fibroblasts to contain cancer progression. However, many of these inhibitory miRNAs are lost with CAF activation by the cancer cells.

When acting to promote tumor progression, exosomes in the TME traffic between all the cell types populating it [246,256,257,258,259], both in primary tumors and at metastatic sites [260]. They induce a multitude of changes, including ones involved in tumor initiation and progression, resistance to a spectrum of therapies, tumor cell EMT, increased vascular permeability, microtubule formation, and tumor migration in vascular endothelial cells [246,249,261]. In BC, exosomes induce resistance to angiogenesis inhibitors through the transfer of VEGF [262], the proliferation of CAFs through the transfer of miR105 from BC cells [263], the transfer of miR200c/114 and miR126a from cancer-to-cancer cells [258], from cancer to endothelial cells, TH1 T cells [259] and NK cells [257], resulting in local suppression of the immune response [246]. In addition, exosomes may also mediate systemic immunosuppression that antagonizes anti-programmed-death ligand (PD)-1 checkpoint therapy [264]. CAF-derived Wnt2 also induces tumor angiogenesis [265].

Exosomes also play a major role in BC pathogenesis, specifically in promoting primary cancer development, invasion, metastasis, and chemotherapeutic resistance [266]. Exosome proteins from ER^+^ and ER^−^ BC cells have both common and disparate members [115]. MCF-7 and MDA-MB-231 cells have 27 exosomal proteins and several oncogenic miRNAs in common. Examples of exosomal proteins common to both cells include members of the annexin family of calcium-dependent phospholipid-binding proteins, involved in the regulation of cellular growth and in signal transduction pathways, histone H4 protein, involved in epigenomic cell alterations associated with increased malignant properties, and the AKT pathway modulator calmodulin, associated with a poor prognosis in BC patients. MDA-MB-231 exosomes have higher amounts of MMPs, consistent with their greater invasive properties, while MCF-exosomes have greater concentrations of nucleic acid, protein binding, and transfer proteins [115]. Exosomes from MDA-MB-231 cells contain greater levels of miR-130a associated with colon cancer tumorigenicity through TGB-β/suppressor of mothers against decapentaplegic (Smad) signaling and miR-328, which targets CD44 to reduce cell adhesion, enhance migration, and regulate capillary structure [115]. MCF-exosomes contain higher amounts of mir-301a, implicated as a negative prognostic indicator of lymph node-negative invasive cancer, miR-34a, which regulates p53 and miR-106b, which can promote invasion and metastasis by targeting transcriptional repressor and anoikis regulator (BRMS1) and Rb and inducing EMT [115].

Both CAFs and NFs transfer exosomes to BC cells, but exosomes from CAFs promote BC cell migration and invasion to a greater extent than exosomes form NFs [267]. CAF-derived exosomes have high levels of miR-18b, which promote BC cell migration and metastasis by binding to the 3′ untranslated region (UTR) of transcription elongation factor A-like (TCEAL)7. The miR-18b-TCEAL7 pathway promotes ectopic activation of nuclear Snail, which induces NFκB, EMT, and invasion and metastasis in vivo [267]. MiR-222 is upregulated in CAFs as compared with NFs and is sufficient and necessary to induce a characteristic CAF expression profile and phenotype [268]. mir-222 directly downregulates lamin B receptor (LBR) expression, and miR-222 overexpression or LBR knockdown is sufficient to induce NFs to exhibit CAF characteristics of enhanced migration, invasion and senescence, and to induce increased BC cell migration and invasion [268].

MicroRNA miR-9 is upregulated in various BC cell lines and is expressed at higher levels in primary triple-negative breast CAFs compared to NFs isolated from patients [269]. The transfer of miR-9 from HR^−^ cells and tumors to NFs via exosomes enhances their transition to CAFs and results in their increased motility [269]. miR-9 expression in CAFs induces downregulation of RNA and protein levels of COL1A1 and EGF-containing fibulin extracellular matrix protein 1 (*EFEMP1*), which encodes the ECM glycoprotein Fbln3, increases the expression of MMP-1, and endows the CAFs with an increased ability to migrate and invade [254,269]. In turn, miRNA secreted by CAFs lowers the expression of E-cadherin in cancer cells, increases their motility through modulating genes involved in motility, induces extracellular matrix remodeling pathways, and promotes in vivo tumor growth [269]. Conditioned medium from CAFs containing miR-9 also induces HR^−^ BC cells to acquire resistance to cisplatin [254].

Head and neck SSC cells can reprogram NFs into CAFs, both in vitro and in vivo, through small EV-packaged TGFβ1, which regulates fibroblast conversion through fibronectin modulation and not through canonical TGFβ1 signaling [270]. In pancreatic cancer, CAF-derived EVs containing elevated levels of miR-331-3p enhance the proliferation migration and invasive potentials of cancer cells by inhibiting scavenger receptor class A member 5 (SCARA5) expression and activation of the focal adhesion kinase (FAK) pathway [271].

Lung cancer cells transform NFs into CAFs by causing an increase in CAF miR-31 and a decrease in their miR-1 and miR-206 content, effects which induce the expression of VEGFA/CCL2 and FOXO3a and promote tumor angiogenesis, accumulation of tumor-associated macrophages, tumor growth, and lung metastasis [143]. Similarly, intrahepatic cholangiocarcinoma cells downregulate miR-206 in NFs and convert them to CAFs [272]. miR-206 suppresses tumorigenesis of intrahepatic cholangiocarcinoma by inhibiting cell proliferation, migration, and invasion [272]. However, incubating the cancer cells with CAFs that were converted promotes cancer progression and gemcitabine resistance [272]. In co-culture with lung cancer A549 cells, CAFs transfer programmed death ligand (PD-L)1 via exosomes to the cancer cells and decrease the apoptotic effects of peripheral blood mononuclear cells (PBMC) [273]. CAF exosomes also contain high levels of Opa-interacting protein 5 antisense RNA (OIP5-AS)1, which significantly downregulates miR-142-5p [273] and miR-34a [274] by acting as a sponge, thereby upregulating PD-L1, suppressing PBMS killing activity and promoting tumor progression.

Non-small cell lung cancer CAF-derived exosomes have higher concentrations of miR-210 than NFs [275]. miR-210 is taken up by lung cancer cells, inhibits UPF1 RNA helicase and ATPase (UPF1) involved in mRNA nuclear export and surveillance and phosphatase and tensin homolog (PTEN), but activates the PTEN/PI3K/AKT pathway and broadly enhances cancer cell migration, proliferation, invasion and EMT status [275]. Mouse pulmonary fibroblasts co-cultured with Lewis lung carcinoma cell line-derived exosomes secrete increased amounts of CCL1, which induces differentiation of Tregs through their specific receptor CCR8 [276]. The activated Tregs, which are immunosuppressive and present ubiquitously in tumors, contribute to the establishment of immunologically tolerant polymorphonuclear leukocytes (PMNs) and a potential pre-metastatic niche in lungs [276].

In cancer of the esophagus, miR-27a/b induces αSMA and TGFβ expression in NFs converting them to CAFs [277]. Esophageal cancer cells cultured in supernatants of miR-27a/b-transfected normal fibroblasts have reduced chemosensitivity to cisplatin, an effect mediated by TGFβ [277]. High serum miRNA levels in patients with esophageal cancer correlate with poor response to chemotherapy [277]. CAFs from patients with gastric cancer have upregulated levels of miR-106b compared with NFs from normal gastric mucosa [278]. miR-106b inhibits the expression of PTEN, a tumor suppressor with much lower expression in gastric cancer CAFs from patients than in NFs, and induces higher levels of migration and invasion in vitro [278]. High expression levels of miR-106b and low expression levels of PTEN in CAFs are associated with poor survival in patients with gastric cancer by Kaplan–Meier log-rank test analysis [278].

Normal murine pancreatic fibroblasts are transformed to CAFs in co-culture with pancreatic cancer cells or microvesicles isolated from pancreatic cell cultures through the transfer of miR-155, which downregulates tumor protein p53-inducible nuclear protein 1 (TP53INP1) [253].

The conversion of NFs by CRC-derived EVs into CAFs is inhibited by long non-coding RNA (LNC) LINC01915 [279]. EVs stimulate NF proliferation, migration, and angiogenesis and facilitate NF conversion into CAFs. However, LINC01915 inhibits the uptake of CRC-derived EVs by NFs through the miR-92a-3p/Kruppel-like factor (KLF)4/CH25H axis, thus arresting angiogenesis and the conversion of NFs into CAFs and in turn, preventing tumor growth [279]. In fact, the overall survival of CRC patients with low LINC01915 expression is much lower than that of patients with high LINC01915 expression [279]. Expression profiling with enrichment analysis demonstrates that secretory exosomes from CRC cell line HCT116 cells contain significantly higher levels of miR-10b than do exosomes from normal colorectal epithelial cells [280]. A bioinformatics analysis of normal CRL1554 fibroblasts with transferred exosomes containing miR-10b identified phosphatidylinositol-4,5-bisphosphate 3-kinase catalytic subunit alpha (PIK3CA) as a potential direct target whose expression is inhibited by miR-10b [280]. Co-culture of exosomes containing miR-10b with fibroblasts significantly suppresses PIK3CA expression in fibroblasts and decreases PI3K/AKT/mTOR pathway activity, reduces their proliferation, and converts them to CAFS expressing TGFβ and αSMA, which are able to promote CRC growth in vitro and in vivo [280].

In vitro culture of human colon cancer stem cells can generate MSC-like cells that are comparable to BM MSC, as defined by surface antigens and the capacity for multilineage differentiation to osteocytes and adipocytes [281]. The exosomes from cancer-derived MSCs are enriched in miR-30a and miR-222, as compared with the miRNAs from the cancer-derived MSCs themselves [281]. miR-30a and miR-222 bind and suppress the expression of their shared downstream target MIA3 and promote colon cancer cell proliferation, migration, and in vivo metastasis [281].

Ovarian CAFs have decreased expression of miR-31 and miR-214 and upregulated expression of miR-155 compared with normal or tumor-adjacent fibroblasts [252]. The transfection of miR-155 and genetic repression of miR-31 and miR-214 convert NFs into CAFs [252]. Converted NFs and CAFs share a large number of chemokines important in CAF function, with the most prominent effect impacting CCL5, a direct target of miR-214 [252].

Melanoma exosomes carrying miR-155 and miR-210 are rapidly taken up by human dermal fibroblasts and lead to a decrease in oxidative phosphorylation (OXPHOS) and an increase in aerobic glycolysis and extracellular acidification [206]. An acidified microenvironment in the primary tumor site favors tumor growth and progression and may favor pre-metastatic niche formation [206]. CAFs can associate with circulating tumor cells in clusters and appear to increase their metastatic efficiency [282]. CAFs are found in PC bone metastatic sites and have emerged as important factors in the development of metastatic outgrowth [283]. Hence, cancer cells modify primary and bone marrow stroma with which they are in direct contact, as in turn, stroma modify the behavior of primary cancer cells and macro- and micrometastases. The 2D modular co-culture methods we have used can successfully query these interactions using a variety of available methods [22,134,138].

The induction of MSCs or fibroblasts into CAFs is not limited to carcinomas [251]. MEC-1 chronic lymphocytic leukemia cells (CLL) generate exosomes containing miR-146 at much higher concentrations than the parental cells [251]. When exosomes are co-cultivated with BM MSCs, they upregulate miR-146 in the MSCs, upregulate the expression of CAF markers αSMA, FAP and downregulate the expression of the tumor suppressor ubiquitin-specific peptidase 16 (USP16) by binding to its 3′UTR, which is the mechanism of induction of CAF transition by exosomal miR-146 [251].

In the tumor microenvironment, both cancer cells and NFs secrete exosomes that contain proteins and microRNAs (miRNA), establishing a cell–cell communication network [115,116]. Before activation of CAFs, NFs have inhibitory capabilities, which they exert to suppress the cancer. This is limited temporally by the initiatives undertaken by cancer cells to convert them into CAFs. Direct contact co-culture, or confrontational culture [111] of colon cancer cells with BM MSCs induces MSCs to exhibit typical characteristics of αSMA-expressing CAFs through activation of Notch and the TGF-beta/Smad signaling pathway [284].

miR-1-3 expression in BC cells inhibits viability, invasion, migration, EMT, tumor formation, and metastasis [119]. Similarly, NFs express miR-1-3 and transfer them to cancer cells via EVs [119]. CAFs have decreased miR-1-3 levels, which may be a mechanism for the loss of fibroblast-mediated suppression of BC [119].

Fibroblasts transfer miR-34a-5p in exosomes to oral squamous cell carcinoma, suppressing their proliferation and metastasis by binding and suppressing AXL-mediated signaling [120]. AXL tyrosine kinase promotes tumor progression through AKT/GSK-3β/β-catenin signaling, β-catenin nuclear-translocation-induced SNAIL transcription, and MMP-2 and MMP-9 activation. mir-34a-5p expression is significantly decreased in CAFs, resulting in a loss of this tumor suppressive effect [120]. NFs also inhibit HNSCC tumor growth and de-repress BCL2 expression in vivo through the transfer of exosomes containing miR-3188, but this miRNA is also significantly reduced in CAFs [117]. The expression of miR-124 suppresses the invasive potential and downregulates αSMA and FAP expression in tissue fibroblasts [118].

miR-101 is the most downregulated miRNA in non-small cell lung cancer CAFs compared to NFs [285]. It enhances apoptosis and directly targets CXCL12, significantly impairing its ability to stimulate tumor cell proliferation, sphere formation migration, and invasion [285]. The downregulation of mir-101 in CAF exosomes releases their tumor-promoting effects through CXCL12 [285].

The expression of miR-124 suppresses the invasive potential and downregulates αSMA and FAP expression in tissue fibroblasts, including normal ovaries [118]. The levels of miR-124 mRNA are significantly lower in ovarian cancer tissue than in adjacent normal tissue or normal ovaries and in ovarian cancer cell lines compared to normal ovarian surface epithelial cells [118]. miR-124 mRNA transported via exosomes from nontransformed ovarian surface epithelial cells to normal tissue fibroblasts suppresses CAF-associated phenotypes [118].

miR-148b functions as a tumor suppressor in endometrial carcinoma cells by directly binding and suppressing DNA methyltransferase 1 (DNMT1) and its effects of inducing EMT, thereby suppressing endometrial cancer metastasis [286]. Endometrial cancer cell CAF exosomes have significantly lower miR-148b levels than normal fibroblasts and promote endometrial cancer cell invasion and metastasis, as they have lost the suppressive effects of the exosomal transfer on mir-148b on DNMT1-mediated EMT [286].

In contrast to the effects of β-catenin signaling in normal dermal fibroblast, which are instrumental in NF suppression of melanoma development, discussed earlier [113], activated melanoma CAFs depend on β-catenin signaling to respond to melanoma stimulation and support melanoma growth in vitro and in vivo [287].

#### 3.2.4. Fusion

Cancer cells form hybrid cells with other cancer cells and with non-cancer cellular elements of the tumor microenvironment, including fibroblasts, mesenchymal stem cells, and macrophages, which endow them with newly acquired phenotypes and capabilities of increased metastatic potential, survival capabilities, including chemotherapy and radiotherapy resistance, and foster tumor progression (comprehensively reviewed by Hass et al. (2021) [288]. Fusion of cancer cells with cells in the tumor microenvironment can also function as a natural defense against cancer, including correction of genetic and/or phenotypic changes underlying malignant transformation, aiding the stromal non-malignant cells in combatting tumor growth, providing neoantigens that elicit anti-tumor immune responses or promoting the function of antigen-presenting cells in generating an anti-tumor response [289]. The resulting phenotype of the fused cell varies with the cancer cell type, origin, and genotypic profiles positioning along the epithelial–mesenchymal and tumor-initiating spectra, the origin, status, and stem cell characteristics of the microenvironmental fibroblasts or mesenchymal stem cells.

Cell fusion is a normal event in normal cellular physiology, for example in mononuclear precursor fusion in osteoclast formation, and is a tightly regulated process [173]. The process, characterized by an initiation and a termination phase, is divided into five discrete steps: (1) priming, (2) chemotaxis, (3) adhesion, (4) fusion, and (5) post-fusion [290]. The process appears to depend on F-actin cytoskeletal structures [291]. These events are followed by a post-hybrid selection process (PHSP) encompassing a remodeling of DNA stability and adaptation to normal cell metabolism [23,292]. Cell fusion may also play a role in premalignant tumor progression. In normal human prostate cells, their senescent characteristics, which depend on the loss of p16^INK4a^ and human telomerase reverse transcriptase (hTERT) expression, also favor fusion with low tumorigenic subsets of AR^+^ LNCaP PC cells [293]. These fusion events result in highly aggressive hybrid cells with enhanced tumorigenicity [293]. The enhanced tumorigenicity depends on maintaining the loss of p16INK4a and hTERT and is inhibited by exogenous p16^INK4a^ and hTERT, needed for maintaining genomic stability [293].

In contrast to differentiated tissue, the cancer microenvironment presents a highly fusion-permissive bioenergetic and physical set of circumstances that enable a process termed “accidental cell fusion” [173]. Tumor pH gradients, hypoxia, damage-associated molecular patterns, membrane lipids, destabilizing ions, and peptides [205,294,295], increased matrix stiffness [230,296], and NFκB and TNFα-mediated signaling [297] collectively generate an environment permissive for cell fusion [23,205,294]. This uncontrolled process leads to progressive heterogeneity of both cancer cells and stromal cells [173,298,299], and cancer cell invasion [205]. The prevailing hypoxia induces apoptosis, which stimulates fusion between BC cells and MSCs, with hybrid cells acquiring enhanced migratory capacity [300]. Other mechanisms of cancer hybrid cell development are entosis, emperipolesis, cannibalism, therapy-induced polyploidization/endoreduplication, and horizontal or lateral gene transfer [288]. All of these processes occur at various rates and are enabled by the same microenvironmental factors that generate a permissive environment for fusion, and result in the progression toward cancer heterogeneity and treatment resistance [288]. The acidified microenvironment in the primary tumor site not only favors tumor growth and progression but may also predispose to the formation of the pre-metastatic niche [206]. Fusion of tumor cells with other tumor cells or with normal microenvironmental premalignant epithelial cells, stromal fibroblasts, and other cells populating the stroma takes place as part of tumor progression and contributes to the genomic instability of the cancer cells, as well as to that of fibroblasts and other supporting cells [299].

The frequency of cancer cell microenvironmental non-malignant cell fusions are generally accepted as being low, 0.1–2%, both in vitro and in vivo [297,301], but their actual frequency in vivo is difficult to gauge because the resulting hybrids may lose their mesodermal or hematopoietic markers due to induced genomic instability, rendering them indistinguishable from unfused tumor cells [299]. The fused hybrid progeny display divergent behaviors and exhibit permanent genomic hybridization, modeling one mechanism of in vivo development of cancer cell heterogeneity [302] and the development of specific aggressive cancer types [303]. The spectrum of divergent possibilities includes apoptosis, necrosis, senescence, dormancy, proliferation, or invasion [23,155,173]. In addition, fusion may also serve to generate protective mechanisms, whereby natural defenses develop against tumor progression through correction of genetic or phenotypic changes in trans, or expression of neoantigens able to elicit immunity or generate effective antigen-presenting cells [289,304].

Cancer cells hijack CAF-derived functional mitochondria through the formation of cellular bridges [305]. CAFs transfer mitochondria to PC cells to serve as energy sensors and transducers of CAF-dependent catabolic reprogramming [305]. MSCs also donate mitochondria to neoplastic cells of various types and induce chemoresistance [306,307]. On the other hand, they can also restore impaired mitochondria function, including activity and normal function of the electron transport chain complexes and functional oxygen consumption and respiratory control, cellular behavior, aerobic viability, and oxidative phosphorylation-reliant cellular motility [308,309].

The fusion of cancer cells with MSCs plays a role in tumor progression and treatment resistance in a number of cancers. Cancer cell fusion with MSCs can restore cancer stem cell/initiating cell properties, including dependence on North American Network Operators’ Group 1 (Nanog1) embryonic stem cell homeobox transcription factor-dependent migration, and plays a likely role in tumor progression [310].

Co-culture experiments between various breast and ovarian cancer cell lines and human MSCs demonstrate significant bidirectional exchange of cell membrane antigens between cancer cells and MSCs, which depend on the formation of intercellular nanotubes, at far higher rates than cell fusion, which result only in a small population of hybrid cells [311]. Nanotube transfer of cellular proteins results in a vast majority of cancer cells that acquire CD90 and CD105 cell surface stem cell proteins and ecto-5′-nucleotidase (CD73) [311]. This is accompanied by an acquired capability to metabolize 5′cAMP associated with aggressive cancer, which also occurs at the transcriptional level, while cancer cells downregulate a variety of transcriptional regulatory genes [311]. In turn, MSCs acquire the expression of epithelial cell adhesion molecules (EpCAMs), cytokeratins, and epithelial-like differentiation factors [311]. BC cells engulf adjacent MSCs in the tumor microenvironment and generate cells with mesenchymal-like, invasive stem cell signatures consisting of Wnt5A, macrophage scavenger receptor (MSR)1, engulfment, and cell motility (ELMO)1, IL-1 receptor-like (IL1RL)2, zona pellucida-like domain containing (ZPLD)1, and signal regulatory protein beta (SIRPB)1, and a metastatic potential that correlates with their engulfing capacity [312]. Spontaneous fusion between MDA-MB-231 HR^−^ BC cells and primary human MSCs yields more than one population with enhanced telomerase activity and enhanced proliferative capacity [313]. The fused cells have upregulated EMT genes SNAIL2 (SLUG), Wnt5A, collagen, fibronectin, N-cadherin, MMP3 and MMP9, and S100 calcium-binding protein A4 (S100A4), which supports tumorigenic proliferation, a decrease in several epithelial-associated genes, a significant increase in subcutaneous tumor growth and a shortened time to distant organ metastasis when injected into mice [313].

While the proximity of MSCs to SK-OV-3 ovarian cancer cells induces increased tumor growth and liver metastases in a NOD/SCID mouse model, hybrid cells that form from their fusion exhibit reduced proliferative capacity compared to the parental SK-OV-3 cells and fail to develop tumors, regardless of whether they have gene expression profiles similar to those of SK-OV-3 cells or ones similar to MSCs [314]. Spontaneous cell and nuclear membrane fusion of patient-derived endometrial cancer cells with omental adipose-derived stromal cells, which are similar to MSCs, results in fusiform, heterogeneous cells that underwent EMT, with decreased E-cadherin and increased vimentin expression, and migratory ability [315].

MSCs can fuse spontaneously with lung cancer cells and reprogram them to exhibit slower growth and a stem-like, more benign state with p21^Waf1^-mediated growth inhibition, and a reprogramming of terminal differentiation pathways mediated by forkhead box (FOX)F1 [316]. Spontaneous fusion between non-small cell lung cancer (NSCLC) cell lines with BM MSCs provide a nonmutational mechanism to generate highly malignant subpopulations that transition from epithelial to fibroblast-like morphology, with both EMT and stem cell-like gene expression profiles [317]. Lung cancer cells fused with MSCs undergo EMT, characterized by downregulation of E-cadherin and pancytokeratin expression and gain of N-cadherin, Vimentin, αSMA, and fibronectin expression, and increased EMT transcription factors Snail1, Slug, Twist1, Zeb1, and Zeb2, and acquisition of stem cell properties [317,318]. The newly acquired stem cell properties are exhibited by increased expression of stem cell transcription marker CD133, transcription factors OCT4, Sox2, Nanog, BMI1, Notch1, aldehyde dehydrogenase (ALDH)1, and kinesin family member 4 (Kif4), as well as BMI1 proto-oncogene, polycomb ring finger (Bmi1), increased pneumosphere-forming capacity and tumor-forming ability in NOD/SCID mice [317,318]. Characteristic of EMT, they have enhanced metastatic capacity with increased expression of MMP-2 and MMP-9 but inhibited proliferative capacity secondary to cell cycle inhibition by elevated cyclin-dependent kinase inhibitors p21^Waf1^, p27^KIP1^ and of p53 [318].

Resulting hybrids of human HepG2 cells with low metastatic potential fused in vitro with rat BM MSCs have reduced expression of E-cadherin, increased expression of vimentin, Twist, Snail, MMP-2, and MMP-9, are aneuploidy, have enhanced invasion and migration in vitro and increased numbers of metastatic liver and lung lesions in vivo in xenograft assays compared to parental cells [319].

Experimental fusion using polyethylene glycol of gastric cancer cells with human umbilical cord MSCs results in hybrid cells exhibiting EMT, with downregulation of E-cadherin and upregulation of vimentin, N-cadherin, αSMA, and FAP, increased expression of stemness factors Oct4, Nanog, Sox2, lin-28 homolog A (Lin28), CD44 and CD133, enhanced migration and proliferation and growth of xenograft tumors in vivo [320]. On the other hand, the artificial fusion of esophageal carcinoma cells with human umbilical cord MSCs results in hybrids exhibiting benign transdifferentiation, with decreased cell growth, increased apoptosis, and suppressed tumorigenicity, with striking upregulation of a MAPK pathway inhibitor dual specificity phosphatase 6 (DUSP6)/MAP kinase phosphatase 3 (MKP3) expression able to suppress growth [321]. The plasticity of CAFs in pancreatic cancer is mediated by transcription factor Prrx1, which is critical in CAF activation, in allowing a dynamic switch between a dormant and an activated state, and in exerting a significant impact on pancreatic cancer biology and therapeutic resistance [223]. BM MSCs fused with HNSCC cells develop sustainable drug resistance mediated through IL-6 and epigenetic imprinting, and impart more aggressive in vivo tumor growth [322].

Glioblastoma stem cells fused with cancer-associated BM MSCs exhibit enhanced proliferation, migration, and invasion, mediated by the downregulation of miR-146b-5p [323]. The loss of miR-146b-5p results in the abrogation of its effects on downregulating its target SWI/SNF-related, matrix-associated, actin-dependent regulator of chromatin, subfamily A, member 5 (SMARCA5) and its TGFβ-mediated pro-malignant effects [323]. Human glioblastoma-derived stem/progenitor SU3 cells fused spontaneously in co-culture with C57Bl nude mouse BM MSCs also exhibit tube-forming ability in vitro and generate solid tumors with CD105^+^ blood vessels in vivo [324]. When the SU3 cells are injected in vivo into the caudate nuclei of mice, they form tumors in which SU3 cells fused with C57Bl mouse CD105^+^ BM MSCs cells can be identified [325]. Fused cells have elevated vascular endothelial cell markers CD31, CD34, and VE-Cadherin, as well as stem cell markers prominin-1 (CD133), Nanog, octamer-binding transcription factor (Oct) 4, and Sox2, and exhibit increased angiogenicity in vitro and in vivo, potentially related to their stem cell properties [325].

Patient-derived pleiomorphic sarcoma cells undergo spontaneous fusions, where the resulting cells exhibit highly malignant and metastatic traits when transplanted into mice [326]. Fusion of transformed MSCs recapitulates this behavior and results in large-scale genomic rearrangements and highly aneuploid aggressive malignant cells with metastatic capacity with traits that include those of rare human mesenchymal pleomorphic sarcomas [326].

Cancer cells also fuse with macrophages and enter the circulation as dual-positive fusion hybrids that have acquired macrophage-associated features, endowing them with accelerated growth, increased motility, enhanced genetic and epigenetic heterogeneity, enhanced invasive activity and higher efficiency in metastasis formation, chemotherapy resistance, and immune tolerance [327,328]. Wnt/β-catenin-mediated fusion with macrophages also endows EMT and stem cell characteristics to BC cells, with an increased migratory and invasive capacity [329]. Cancer stroma fusion hybrids in vitro can be propagated at low-density replating for colony formation into derivative cell populations.

While generalizations on the direction of the effects of CAFs on cancer cells are difficult to make, it is clear that the heterogeneity in gene expression profiles in fusion cell products results in emergent clones with variable responses due to the preponderance of either mesenchymal or epithelial signaling. Clones with genes positively regulating EMT are prone to motility, invasion, and metastasis, and potentially dormancy and acquisition of stem cells traits [330], while cells that acquire genes regulating epithelial characteristics, including proliferation, emerge as growing clones in the primary tumor, enter the circulation and lay the basis for metastatic outgrowth. Variables that contribute to this dichotomy include the origins of CAFs from the heterogeneous populations of NFs and MSCs, the time that the fibroblast spends in contact with the cancer cells, and the limited opportunity to exert cancer inhibitory effects before it gets activated [331]. The number of passages in the culture must be considered before accepting the results of gene expression profiling data from cultured cells. Further, population-averaged analysis has been replaced by single-cell multimodal molecular analysis that has endowed a better understanding of the dichotomy of the effects that CAFs exert over cancer progression.

### 3.3. Differences between CAFs and NFs

#### 3.3.1. Isolation of CAFs

The study of the molecular characteristics and gene expression patterns of CAFs has been made practical by techniques used to isolate fibroblasts from primary and metastatic tumors. Investigators use well-described methods similar to the ones outlined above for isolating NFs from a variety of tissues. Generally, tumors and adjacent non-cancer tissue are minced into 1–2 mm chunks in Roswell Park Memorial Institute (RPMI)-1640 or Dulbecco’s Modified Eagle Medium (DMEM). The tissue chunks are digested with combinations of trypsin, collagenase, and 0.1 μg/mL DNase, 0.1 mg/mL hyaluronidase for 2 to 3 h at 37 °C three times, purified by using magnetic beads, cultured in fibroblast growth medium-2 (FGM-2, Lonza) and sorted with anti-fibroblast microbeads [118,332]. Many other routine isolation protocols are outlined in the references on CAFs. Alternately, CAFs can be isolated using a commercial tissue dissociator with subsequent enzymatic digestion, filtering through a 100 μm strainer, centrifuging for collection, lysing red blood cells, and analyzing for antigen expression [69]. CAFs can be characterized by immunofluorescence microscopy or flow cytometry using antibodies to αSMA, FAP, FSP-1, and vimentin, antigens robustly expressed by CAFs, while weakly expressed by NFs [279,333].

Once purified, CAFs and NFs can be analyzed for molecular differences using a variety of standard, population-averaged molecular techniques, including gene expression microarrays and RNAseq, as well as single-cell RNAseq technology [69,70,76]. In addition, fibroblasts can be imaged in situ using an array of in situ sensors [83]. These include force biosensors, imaging reporters for single-cell kinase signaling and FRET-based biosensors for kinase signaling [83]. They also include a variety of labeled compounds for measuring metabolic activity, glucose uptake in cancer cells, intracellular adenosine triphosphate (ATP) levels, nicotinamide adenine dinucleotide (NAD)^+^, pyruvate, lactate, cell–cell interactions, used as above in the NF section and for determination of CAF paracrine signaling for inducing EMT in cancer cells [83].

#### 3.3.2. Distinctions in Molecular Profiles between NFs and CAF

Systematic investigations to differentiate NFs from CAFs have identified the NF marker CD39 as belonging to normal healthy tissue and CAF markers CD87, CD44, CD49b, CD95, and Ly-6C as being associated with mouse tumors [334]. The data demonstrate a functional association of most cancer-related fibroblast markers with proliferation and a systemic upregulation of CD87, and CD49b in tumor-bearing mice, even in tissues not affected by the tumors [334].

Transcriptomes from BC stroma primarily consist of ECM components and genes involved in the remodeling of the ECM, as compared with those from normal stroma [335]. NFs inhibit pre-malignant MCF-10AT cell proliferation in transwell and more so, in direct contact co-culture, while CAFs are less growth inhibitory and able to promote epithelial cell growth [336]. In a gene array analysis, 420 genes were differentially expressed between the two fibroblast populations [336]. rtPCR and immunohistochemistry validation in breast tissue reveal upregulated dickkopf 1, neuregulin 1, plasminogen activator inhibitor 2, and tissue plasminogen activator (TPA) in CAFs, and Fbln1 upregulation in NFs [336]. Fbln 1 expression is higher in ERα^+^ cancers, and low stromal expression of Fbln correlates with higher proliferation in cancer epithelial cells [336].

miRNA microarrays of CAFs cultured from previously untreated resected breast tumors demonstrate three upregulated miRNAs, miR-221-5p, miR-31-3p, miR-221-3p, and eight downregulated miRNAs, miR-205, miR-200b, miR-200c, miR-141, miR-101, miR-342-3p, let-7g, miR-26b when compared to NFs [337]. The collective group of target genes affects cell differentiation, adhesion, migration, proliferation, secretion, and cell–cell interaction [337]. CAFs and NFs cultured from early BC tissue are much more vigorous in their proliferation, migration and invasion potential than tissue NFs [338]. Population-averaged gene expression profiling of heterogeneous fibroblast populations demonstrates upregulated cell cycle, cell adhesion, signal transduction and protein transport pathways derived from gene ontogeny databases reported from other tumors [338]. In co-culture, CAFs promote BC cell invasion through secreted CCL18 and CXCL12 [338].

Significant differences are noted between premenopausal and postmenopausal normal mammary fibroblasts [339]. Premenopausal breast fibroblasts have elevated MMP10, MMP3, CXCL8, CXCL1, cathepsin L (CTSL), mesenteric estrogen-dependent adipogenesis (MEDAG), CXADR-like membrane protein (CLMP), podoplanin (PDPN) and phosphoglycerate mutase (PGAM)1 [339]. In contrast, fibroblasts in the postmenopausal breast have elevated ubiquitin (UBC), heat shock protein (HSP)AB, DnaJ heat shock protein family (Hsp40) member B1 (DNAJB1), HSPA1A, early growth response protein (EGR)1, FOS, JUN, activating transcription factor (ATF)3, KLF4, MAF bZIP transcription factor (BMAF)B [339]. The analysis of differences between NFs and cancer-derived fibroblasts is fraught with challenges based on the substantial variation in the stromal and immune composition of different patient samples [339]. This reflects the inherent tissue diversity of cancers, as well as the location of the specimen within tumors, the timing between tissue collection and processing and the precise digestion protocol used to isolate the diverse cell types [340]. Within these constraints, BRCA1-mutant tumors demonstrate decreased PDGFRA^+^ fibroblast and MCAM^+^ pericyte populations [339]. Single-cell gene profiling analysis of CAFs demonstrates increased levels of collagen COL1A2 and COL3A1, the WNT inhibitor secreted frizzled-related protein (SFRP)2, and the calmodulin- and actin-binding protein glutamine amidotransferase class 1 domain containing 1 (GALDI), which mediates the contractile function of myofibroblasts [339]. Tumor tissues also exhibit marked changes in the immune microenvironment, infiltration of immune cells and macrophages, with infiltration of CD8^+^ T cells characterizing triple-negative and HER2^+^ cancers but not ER^+^ tumors [339]. Greater than 90% of BC CAFs, especially HR^+^ cancer CAFs, express the glucocorticoid receptor, with its expression correlating positively with tumor grade, Ki-67 index, and expression of the glucocorticoid receptor in the cancer cells [341].

Protein tag profiling of PC CAFs and NFs identified 671 transcripts that were enriched in CAFs and 356 transcripts whose levels were decreased relative to NFs from prostate tissue. Gene ontology analysis revealed that enriched transcripts in CAFs are associated with upregulated processes involving prostate gland morphogenesis, development, and growth, the retinoic acid receptor signaling pathway, and uterus, ovarian, and vaginal development, while downregulated processes in CAFs involve all phases of the cell cycle, mitotic machinery, nuclear and organelle division [342]. Using the Human Protein Atlas, the study found seven tag protein candidates that are higher in CAFs than in NFs, the cartilage extracellular protein asporin (ASPN), cathepsin K (CTSK), FBLN1, fibronectin (FN)1, osteoglycin (OGN), the actin-binding protein Parvin alpha (PARVA) and ZEB1 [342]. The investigators also found five tag proteins that are depleted in CAFs, CAV1, complement factor H (CFH), natural killer cell triggering receptor (NKTR), S100A6 and stanniocalcin (STC)1, compared to NFs [342]. Asporin is a stroma-derived inhibitor of TGFβ1 and a tumor suppressor in BC, where high asporin expression is significantly associated with less aggressive tumors when stratifying patients according to the clinical outcome [343]. It appears that CAFs from PC and other cancers adopt a gene expression pattern that favors the balance to a mesenchymal program over an epithelial, proliferative program found in NFs. However, the upregulation of CXCL12, hypoxia-inducible factor (HIF)1α, glial cell line-derived neurotrophic factor receptor (GFR)A1, ERα, Hes1, and c-fos-induced growth factor (FIGF, VEGFD) [344,345], and the downregulation of prohibin (PHB), WAP four-disulfide core domain 1 (WFDC1) and cerebral adrenoleukodystrophy (CALD) in CAFs [346,347] collectively promote cancer cell proliferation and angiogenesis. These results and experimental evidence support the concept that the presence of significant stromal fibroblast heterogeneity is an ever-present and necessary factor in the promotion of tumor progression [347].

Comparative secretome analysis conducted by 2D gel electrophoresis, matrix-assisted laser desorption/ionization time-of-flight (MALDI-TOF) mass spectrometry (MS) identified 11 proteins differentially secreted between CAFs and NFs isolated from HNSCC [348]. These proteins are fructose bisphosphate aldolase A, plasminogen activator inhibitor 1, cathepsin L, membrane annexin A5, 14-3-3σ protein, cystatin C, complement component C1s precursor, Cu/Zn-superoxide dismutase (SOD), heterogeneous nuclear ribonucleoprotein A1, Rho-GDP dissociation inhibitor 1 and Galectin 1 [348], and play roles in tumor progression by protecting cells against ROS injury, in protein degradation, invasion and metastasis signaling [348].

CAFs isolated, cultured, and purified from HNSCC oral cavity tumors have greater proliferative ability and less apoptosis compared to NFs purified from normal tissue [73]. Unlike cancer cells, CAFs preferentially use oxidative phosphorylation over glycolysis and have stronger maximal respiration, greater mitochondrial spare respiratory capacity (SRC), and higher adenosine triphosphate (ATP) production capacity than NFs [73]. In this context, CAFs have higher ATP synthase subunit O (ATP5O) and lower tumor necrosis factor receptor-associated protein 1 (TRAP1) expression in the mitochondria than NFs, effects responsible for increased basal oxygen consumption, maximal respiration, ATP production, spare respiratory capacity, and decreased glycolysis [73]. TRAP1 is the only mitochondrial member of the HSP90 family that regulates a metabolic switch between mitochondrial respiration and aerobic glycolysis. Its expression is downregulated in tumors along with 26 differentially expressed proteins associated with OXPHOS, and indeed, enforcing its expression in CAFs suppresses tumor growth in vivo [73].

Investigations have demonstrated that loss of organ-specific gene expression patterns that differentiate normal fibroblasts are downregulated in CAFs in lung cancer [70]. Similarly, genes with unique, high expression in colon submucosal fibroblasts relative to other fibroblasts are downregulated in CRC, including the NK2 homeobox 3 (NKX2)-Wnt family member 2B (WNT2B), an effect that may be related to the development of colorectal CAFs [70,349]. Wnt family members, including Wnt3s, activate CRC CAFs [349].

There is a potential subtlety and context-dependent dichotomy in the reported effects of CAV1, a scaffolding protein which is involved in several cancer-associated processes, that of having both protumorigenic and tumor suppressive roles [350]. This may be associated with differences in localization of its two different isoforms. CAV1β is more prominent in shallow caveolae and CAV1α is predominantly observed in deep caveolae, resulting in a higher ratio of CAV1α/CAV1β in deep caveolae [351]. In HNSCC, investigators determined that the CAV1β isoform is more highly expressed than CAV1α in both CAFs and NFs, but the two isoforms differ in their oligomerization profile, with NFs expressing higher levels of the higher molecular weight oligomerized isoform of CAV1s than CAFs [350].

### 3.4. Acquisition of CAF Heterogeneity

CAF heterogeneity has been expertly reviewed by Pradhan et al. (2021) [39] and Walter et al. (2021) [352]. These reviews underscore the importance of the pre-existing heterogeneity of the fibroblasts in the normal organ in order to understand the origin and function of turned CAFs in the tumor microenvironment and metastases [39]. They outline the various characteristic functional and phenotypic categories of CAFs, with roles in tumor proliferation, angiogenesis, chemoresistance, inflammation, immunosuppression, immunomodulation, ECM deposition and remodeling, invasion, metastasis, as well as tumor suppression [352]. It is also clear that the tumor microenvironment, noted for pH gradients, hypoxia, and destabilizing ions, described above, induces DNA and membrane lipid damage, results in gene expression changes, and contributes to fusion, which, in turn, contributes to the development of CAF heterogeneity [173].

Here, I outline some of the data that describe genomic instability in CAFs and the functional heterogeneity of CAFs that occur as a result, with a focus on differentiating characteristics that promote tumor progression from those that resist it. I also outline reports of CAF and NF heterogeneity originating from racial characteristics and backgrounds of patients that may impact differences in characteristics, aggressiveness, and treatment response of cancers.

#### 3.4.1. Genomic Instability of CAFs and Co-Evolution with Cancer

Cancer cells generate oxidative stress in adjacent fibroblasts, mimicking the effects of aerobic hypoxia, which initiate anti-oxidant anti-apoptotic defense mechanisms in the stroma and feedback autophagy and mitophagy in the cancer cells, which drive metabolic and mutagenic cancer cell co-evolution, DNA damage and aneuploidy [353,354]. Cancer cell autophagy results in the overproduction of recycled nutrients, ketones, and L-lactate, and drives mitochondrial biogenesis and anabolic cancer cell growth via an energy imbalance [354]. Cancer cells with mesenchymal transcription programs at the invasive front exhibit higher levels of autophagic flux than cancer cells inside the tumor body in primary human NSCLC, which endows them with invasive behavior and positively correlates with advanced tumor stage and reduced patient survival [355]. Signaling in CAFs activates HIF1α and NFκB, potentiating a hypoxic and inflammatory response, and also upregulating the autophagic program in the stroma [354]. Stroma ROS leads to DNA damage, aneuploidy, and genomic instability in fibroblasts [354]. Specifically, oxidative stress induces a loss of CAV1 expression in CAFs, which leads to nitrous oxide overproduction, mitochondrial dysfunction, oxidative stress, aerobic glycolysis, and elimination of the defective mitochondria by autophagy/mitophagy [353]. The modified CAFs, in turn, provide lactate to stimulate mitochondrial biogenesis and oxidative metabolism in adjacent cancer cells through a bystander effect, known as the “Reverse Warburg Effect”, thereby increasing their malignant behavior [353].

The metabolic shift to aerobic glycolysis and its induction of cancer progression are inhibited by alpha/beta-hydrolase domain-containing 5 (Abhd5), an intracellular lipolytic activator [356]. Knockout of Abhd5 in mouse intestine robustly increases malignant transformation of adenomatous polyps, induces EMT in colon cancer by suppressing the AMPκ-p53 pathways, and its silencing in normal fibroblasts induces malignant transformation through increased aerobic glycolysis [356].

CAFs generated from normal skin and lung fibroblasts by non-contact co-culture with breast, brain, lung, and PC cells at in-vivo-oxygen tensions decrease their expression of CAV1 and p21^Waf1^, begin expressing CAF biomarkers and undergo perturbations in their redox environment [357]. This is evidenced by increased protein carbonylation, increased mitochondrial superoxide anion levels, and modulation of the activity of the antioxidants manganese SOD and catalase [357]. Continued propagation of CAFs for 25 population doublings results in enhanced genomic instability and decreased expression of senescence markers β-galactosidase and p16^INK4a^, and relative resistance to ^137^Cs gamma irradiation compared with NFs, an effect dependent on enhanced repair capacity of single- and double-stranded DNA breaks [357]. DNA damage response to induction of pro-oxidant and pro-fibrosis genes by TGFβ in NFs and cancer cells also triggers the expression of a wide range of pro-oxidant and pro-fibrosis genes, leading to fibrosis, genomic instability, and radioresistance [229].

Loss of CBF-1, suppressor of hairless, Lag-2 (CSL)/RBP-Jκ, the canonical Notch signaling effector with intrinsic transcription repressive functions, causes the conversion of dermal fibroblasts into CAFs and triggers DNA damage, telomere loss, and chromosome end fusions [358]. CSL is part of a multiprotein telomere protective complex, which binds directly, with high affinity to telomeric DNA and to up frameshift 1 protein (UPF)1 RNA helicase and ATPase and the heterodimer X-ray repair cross-complementing 6 (Ku70/Ku80) non-homologous end-joining repair proteins, and is required for its telomere association, resulting in CAF genomic instability [358].

#### 3.4.2. Molecular Heterogeneity

The heterogeneity of tumor stroma depends on the tumor type, and its interaction with the TME dictates the gene expression profile of the stroma [359]. CAFs modulate the TME and either promote or inhibit the associated tumor, highlighting their pleiotropic functions and their inherent plasticity [298]. Comparison and analysis of gene expression profiles of early passage primary CAFs isolated from BC patient samples representing three main subtypes, ER^+^, triple-negative, and Her2^+^ yielded significant expression differences between CAFs derived from Her2^+^ and CAFs derived from triple-negative and ER^+^ BCs, particularly in pathways associated with cytoskeleton and integrin signaling involved in motility [360]. Canonical pathways selectively upregulated in Her2^+^ compared to ER^+^ and triple-negative BC samples involve the actin cytoskeleton and extracellular matrix, integrins, actin-based motility through Rho and Cdc42, integrin-linked kinase (ILK), PI3K/AKT, phospholipase C, protein kinase A, FAK, serine/threonine p21-activated kinase PAK1 (PAK1), and several other pathways associated with motility, cell structure, guidance, and endocytosis [360]. The frizzled class receptor 1 (FZD1) Wnt signaling protein is notably higher in triple-negative BCs than in Her2^+^ and ER^+^ CAFs [360]. In another study of microdissected stromal cells from patients with both ER^+^ and ER^−^ locally advanced BC, four pairs of markers could correctly classify all tumor samples to a group more likely to respond to neoadjuvant chemotherapy [361]. The markers are mainly involved in immune response or lymphocyte activation, including CD47, LCK proto-oncogene, Src family tyrosine kinase (LCK), NCK adaptor protein (NCK)1, CD24, CD3 epsilon subunit of T-cell receptor complex (CD3E), zeta chain of T cell receptor-associated protein kinase 70 (ZAP70), FOXP3, and CD74, among others [361]. The four pairs of markers are patched domain containing 1 (PTCHD1)/pyridoxal dependent decarboxylase domain containing two pseudogenes (PDXDC2P), Proton Channel OTOP1-like (LOC100506731)/neuralized E3 ubiquitin-protein ligase (NEURL)4, SH2 domain containing 1A (SH2D1A)/CCZ1 Homolog, vacuolar protein trafficking and biogenesis associated (ENST00000478672), and thymocyte selection associated high mobility group box (TOX)/H2A.J histone (H2AFJ) [361].

CAF heterogeneity can be categorized into the inflammatory CAF group with low αSMA expression and high IL-6 expression, the TGFβ-dependent myofibroblast CAFs with high αSMA expression, or tumor suppressive CAFs with inhibitory effects mediated through stroma-specific Hedgehog (Hh) activation [230]. A twelve-gene CD10^+^ stromal cell signature from fibroblasts, myoepithelial, and mesenchymal stem cells associated with breast tumors includes genes involved in matrix remodeling (MMP11, MMP13, and COL10A1) and genes related to osteoblast differentiation (periostin), among others, with mesenchymal stem cells having the highest signature score [362]. The signature can differentiate in situ from invasive BC and is associated with a worse prognosis of HER2^+^ BCs and a lack of response to chemotherapy [362].

An orthotopic murine model or BC revealed a dynamic functional change in CAF transcriptional programs over time of tumor development and in metastases, in line with the investigations outlining a co-evolution of cancer cells and the TME [331]. The TME evolution described in the study by Friedman et al. (2020) [331] outlines a transitioning of the CAF population over time. At two weeks, the CAFs consist of a population expressing an immunoregulatory program, identified by a 70% prevalence of S100A4-pododoplanin (Pdpn)^+^ CAFs likely originating from normal breast tissue NFs [331]. By four weeks, they transition to a population expressing a wound-healing TGFβ-responsive program, populated by a predominantly S100A4^+^Pdpn^−^ CAF population whose origins from BM MSCs recruited to breast tumors were suggested by similarities in their gene expression profiles [39,331]. The Pdpn^+^ CAF population not only dropped to 23% at 4 weeks as tumors progressed, but its heterogeneous gene expression profile changed from an immunoregulatory phenotype characterized by CxCl12, Saa3 to a predominantly TGFβ-response actin alpha 2, smooth muscle (Acta2)^+^ profile [39,331]. The clinical implications were underscored by the fact that the ratio of these two CAF populations is associated with disease outcomes across subtypes and is particularly correlated with BRCA mutations in triple-negative BCs [331].

In a polyoma middle-T murine model of BC, four subsets of CAFs are present [363]. These include vascular CAFs and cycling CAFs, representing CAFs of perivascular origin that eventually invade tumor stroma with cancer progression, matrix CAFs of tumor residential fibroblast origin, and developmental CAFs with transcriptional similarities to vascular CAFs, and are identified as originating from epithelial cells that underwent EMT [363].

Cancer-associated fibroblasts (CAFs) from HNSCC are exceptionally heterogeneous. Single-cell fibroblast transcriptional profiles of 1500 single CAFs from 18 HNSCC patients identified two heterogeneous populations [79]. One of the populations is associated with TGFβ-responsive genes, such as periostin, stromelyin-3 (MMP11), and collagen I and secreted protein acidic and cysteine-rich (SPARC), associated with lymph node metastases [79]. The second population expresses high levels of CXCL12 and CXCL14, FGF-1, and adipocyte markers adipsin and apolipoprotein D (APOD), suggesting an adipocyte fibroblast lineage origin [79]. Interactions between CAFs and malignant cells promote an EMT program at the leading edge of HNSCCs with potential roles in invasion, an effect corroborated by associations of high CAF and EMT valuations in tumors with metastasis [79]. In pancreatic cancer, the expression of SPARC by peritumoral fibroblasts is associated with a poorer prognosis for patients [364]. However, in addition to enhancing stroma formation, SPARC can prevent fibroblast activation, a mechanism endowing it with anticancer effects [365].

In immunohistochemical analyses, SCCs of tongue tumors can be divided into CAF-rich, -intermediate, and -poor categories, with a high density of αSMA^+^ CAFs associated with recurrence and poor survival [366]. The most frequent category of CAFs is myeloid/monocytic-derived cell marker CD86^+^/αSMA^+^, significantly associated with CAF-rich tumors and poor survival, followed by myeloid/monocytic-derived cell marker CD80^+^/αSMA^+^ and mesenchymal stem cell marker Nanog^+^/αSMA^+^ cells, with a CD133^+^αSMA^+^ phenotype only associated with blood vessels [366]. A study of single-cell and bulk tissue transcriptome profiles of HNSSCs from patients identified eight CAF subclusters [367]. Seven of the clusters were expressed to higher levels in tumor tissues than in normal tissue. Three clusters were associated with poorer overall survival, characterized, respectively, by myofibroblast functions with enriched αSMA expression and smooth muscle contraction, another by expression of ECM remodeling and EMT-related genes, and a third with high levels of the major histocompatibility complex (MHC) class II family genes, characterized as antigen-presenting CAFs [367].

In pancreatic cancer, when compared to NFs, CAFs include subpopulations associated with insulin-like growth factor (IGF) signaling, a population expressing lymphocyte antigen complex locus and locus cl (Ly6a/cl) and peptidase inhibitor 16 (Pi16), and a population with mesothelial markers, including mesothelin (Msln) and Cav1, cadherin 11 (Cdhll), and growth arrest-specific 6 (Gas6), with potential immune-modulatory functions [368]. Distinct CAF lineages in pancreatic cancer derive from the existing heterogeneity of normal pancreatic tissue. One lineage, the TGFβ-responsive myofibroblast CAF population, is located in the tumor nests, originates from stellate cells, and generates ECM mRNAs and TGFβ-induced genes such as Col1A1, Col5A1, Ctgf, smooth muscle α-2 actin (Acta2), or vimentin (Vim) [39,68,369,370]. The other lineage is the IL-1-responsive inflammatory CAF population, located distal to tumor nests in a desmoplastic area, coding for increased Jak/STAT signaling pathway members, interleukins, chemokines, and cytokines such as Cxcl1, 2, or 3, IL-6, IL-11, IL-21 and Lif [39,68,369,370]. Secretion of IL-6 promotes TGFβ-mediated EMT in ovarian cancer via the JAK2/STAT3 pathway and leads to the inhibition of apoptosis and paclitaxel resistance [371]. CAFs from a third category, termed antigen-presenting CAFs, expressing high levels of class II MHC and CD74, able to activate CD4^+^ T cells, also originate from pancreatic stellate cells [68,370]. A population of TGFβ-converted CAFs with an elevated leucine-rich repeat containing 15 (LRRC15) protein signature surrounding tumor islets, identified using single-cell transcriptomics in the process of charting pancreatic ductal adenocarcinoma progression, correlates with poor response to anti-PD-L1 immunotherapy across clinical trials comprising more than 600 patients in six cancer types [68].

Investigators have sought some order in the heterogeneity and differential function of CAFs within pancreatic tumors. As noted earlier, tumor/TME interactions do not uniformly promote or inhibit tumor progression. While CAF populations within a tumor are exceptionally heterogeneous, CAF heterogeneity is not random but marks fundamental tissue organizational units [372]. Grunwald et al. (2021) [372] identified three distinct TME phenotypes in human pancreatic cancer samples with characteristic CAF features, single-cell gene expression profiles, and prognostic significance, found in both primary and metastatic sites [372]. The three regions are (1) “deserted”, with thin, spindle-shaped fibroblasts, loose fibers, and frequent myxoid features, exhibiting strong enrichment of ECM, ECM-signaling, and humoral immunity pathways, (2) ‘‘reactive’’ regions containing plump fibroblasts with enlarged nuclei, few acellular components, usually containing inflammatory infiltrates, expressing gene sets related to cellular stress response, growth factors with CAF-activating and immunomodulatory functions, and (3) regions with intermediate levels of combinations of these features [372]. The three phenotypes are termed ‘‘sub-TMEs’’, as they could occur in spatially confined areas of the same tumor simultaneously [372]. They have prognostic survival impacts, with “reactive” subTMEs supporting a proliferative, basal-like, and poorly differentiated tumor cell phenotype with poorer survival than “deserted” TMEs, and the co-occurrence of sub-TMEs having a characteristic poor prognosis [372].

Pancreatic cancer CAF heterogeneity is exemplified by the presence of two subpopulations, one with elevated αSMA located immediately adjacent to neoplastic cells in mouse and human cancer tissue, which produces a desmoplastic reaction, and another subpopulation of αSMA- CAFs more distant from cancer cells that secrete IL-6 and other inflammatory mediators [66]. Within the context of stromal heterogeneity in various tumor models and spontaneous murine and human pancreatic cancers, there is a relationship between mesenchymal and immune cell subsets delineated by two stable and functionally distinct classes of CAFs differentiated by endoglin, an accessory TGFβ receptor (CD105) expression [373]. CD105^+^ CAFs are permissive for tumor growth in vivo, while CD105- CAFS are highly tumor suppressive, an effect dependent on adaptive immunity [373].

Metabolic and single-cell transcription factor regulon activity profiling and KEGG pathway allocation in pancreatic cancer CAFs divides them into three distinct populations: myofibroblasts (myCAFs), inflammatory CAFs (iCAFs), and antigen-presenting CAFs (apCAFs) [374]. The iCAF subtype is represented by complement and coagulation cascades and cytokine–receptor interactions and trended to glycolysis with cAMP-responsive element binding protein 3-like (CREB3L)1, early growth response (EGR)2 and SOX4 activation and is a protective factor associated with an inflammatory phenotype. The myCAF subtype is noted for focal adhesion and ECM–receptor interactions and depends on the tricarboxylic acid cycle and its derivatives with nuclear factor erythroid 2-related factor (NRF)2, CCAAT enhancer binding protein delta (CEBPD) and Y-box binding protein (YBX)1 activation and is an important negative prognosis indicator [374]. The third category, apCAFs, is prominent for antigen processing and presentation [374].

Cancer cells communicate intensely with cancer-associated fibroblasts in endometrial carcinomas, as well as with other cells in the microenvironment, more so than with each other [76]. Fibroblasts signal through direct interactions, through fibroblast-secreted cytokines and growth factors and various receptors. They activate cancer cell signaling pathways and transcription factors, including IGF1/α6β4 complex, FGFR2, TIMP, DNA replication and mTOR signaling, Smad7, KLF4, and TBX2, and downstream proto-oncogenes such as MYC, MET, and CDK1, which facilitate cancer progression [76]. CAFs in endometrial carcinoma, identified by classic fibroblast markers ATCA2, COL1A1, COL3A1, and THY1, are divided into four subsets with distinct characteristic and exclusive expression patterns that perform unique functions in the tumor ecosystem [76]. Specifically, cluster (a) was identified as matrix CAFs with genes enriched in extracellular matrix and structure organization and collagen metabolic processing, and an ECM signature exemplified by glycoprotein (PDPN), structural protein (COL12A1), matricellular proteins (FBLN2 and SOX6) and matrix modifying enzymes (LOXL1 and MMP2). Cluster (b) is the inflammatory CAF population and features high levels of SLPI, IGF1, CD24, CXCL12, and TFF3, ontogeny terms associated with myeloid leukocyte migration, mononuclear cell migration and leukocyte chemotaxis. Cluster (c) is named the vascular CAF cluster and is identified by genes such as MYH11, GJA4, RGS5, ESAM, MCAM and EPAS1, involved in muscle system process, muscle contraction and tissue migration. Cluster d) fibroblasts are identified as antigen-presenting CAFs and express major histocompatibility complex II (MHC-II) genes CD74, HLA-DPA1, HLA-DPB1 and HLA-DQB1, mainly involved in immunomodulation such as T cell activation and leukocyte cell–cell adhesion. The cells in this cluster express high levels of immune checkpoint molecules PDCD1, CTLA-4, LAG3, HAVCR2, TIGIT and ICOS, suggesting they may contribute to an immunosuppressive effect [76]. Combining these data with data from public databases of endometrial carcinoma gene expression, patients in the antigen-presenting CAF cluster, who incidentally had the lowest stroma score, had prolonged survival [76]. In contrast, patients with vascular-associated fibroblasts had the shortest survival, supporting a role for vascular-associated fibroblasts as an independent risk factor due to their effect on restraining the infiltration of immune cells [76].

#### 3.4.3. Racial Differences

Variable social and disease factors contribute to a disparity in survival of African American (AA) patients with BC [375,376,377], but emerging data are making it evident that differences in the molecular characteristics of the cancer and the tumor microenvironment also contribute to the disparities [375,378,379,380,381,382,383,384]. The differences in the molecular characteristics of the epithelial cancer cells and pluripotential stem cells have been and continue to be investigated extensively [380,384,385,386,387]. While the extracellular matrix of AAs has a greater density of protumorigenic immune cells, such as M2 macrophages and regulatory T-cells and of microvasculature, as well as higher levels of inflammatory cytokines [388], it also has fibroblasts with a greater capacity to support the malignant progression of hormone receptor (HR)^−^ BC cells [389]. Here, I outline some of the significant studies that have demonstrated differences in the microenvironment, specifically fibroblasts, and MSCs in benign mammary glands from AA patients that extend our understanding of their exceptional heterogeneity. References below also address race-based differences in cancer-associated fibroblasts (CAFs).

AA ECM induces significantly more aggressive behavior in vitro in ER/PR^−^ cell lines, while Caucasian ECM induces significantly more growth in soft agar and invasive behavior in vitro and tumorigenicity and metastasis in vivo of ER/PR^+^ cells [389]. Primary BC cells develop significantly larger tumors when injected into mouse mammary glands humanized with premenopausal Caucasian donor-derived fibroblasts than with AA fibroblasts [389]. Correspondingly, estradiol, estriol, and 2-methoxyestrone are significantly higher in Caucasian breast tissue. NF-derived gene signatures and secreted molecules and cytokines in premenopausal breast stroma are distinctly different between Caucasian and AA women [389]. Only 1759 of approximately 8000 ECM proteins identified in this study were expressed in common between AA and Caucasian ECM by mass spectroscopy. AA-unique proteins were primarily related to tumorigenesis/neoplasia, consistent with a mesenchymal signature, and included higher testosterone levels and BC-associated proteins related to tumorigenesis/neoplasia, while Caucasian-unique proteins had higher expression of estradiol, estriol, and 2-methoxyestrone, protease activity and growth/metastasis, suggestive of an epithelial signature [389].

CM from dermal fibroblasts obtained from AA skin biopsies had higher levels of TGFβ than those from matched Caucasians and correlated with higher levels of TGFβ mRNA in AA skin analyzed by cDNA arrays [390]. Treatment with TGFβ1 resulted in a considerably higher induction of elastin mRNA in dermal fibroblasts from AAs than from Caucasians, indicative of enhanced TGFβ signaling in AA skin [390]. Dermal fibroblasts of AA patients with systemic sclerosis had a widespread reduction in DNA methylation in CpG sites compared to control AA individuals [391]. Sites with decreased methylation were most enriched in introns and intergenic regions but depleted in 5′ untranslated regions (UTRs), promoters, and CpG islands [391]. Enrichment was prominent in interferon signaling and mesenchymal differentiation pathways. Specifically, hypomethylation of distal-less homeobox 5 (*DLX5*) and transmembrane protein 140 (*TMEM140*) genes was associated with their overexpression in patients and an underexpression of long non-coding RNA (lncRNA) uncharacterized protein *MGC12916* [391]. AA descent positively correlates with high-efficiency iPSC reprogramming in dermal fibroblasts, which is strongly impacted by variations in the expression of the ATPase Brahma-related gene-1 (BRG1), BAF155, and BAF60a, epigenetic enzyme components of the SWI-SNF chromatin-remodeling complex [392].

AAs have a significantly higher rate of profibrotic conditions that are consistent with an altered allostatic load, which is an evolutionary propensity for altered tissue repair, including keloid formation, leiomyomas, glomerulosclerosis and sarcoid [50,393,394,395,396]. Signal pathways associated with profibrotic processes in fibroblasts and macrophages in AA populations that regulate fibroblast plasticity, growth, differentiation, motility, and epithelial–mesenchymal transition (EMT) include TGF-β1, TGF-β2, TGF-β3, TGF-βR1, TGF-βR2, TGF-βR3, Smad3, Smad6, Smad7, epidermal growth factor receptor (EGFR), TNFα induced protein 6, p53, and multiple human leukocyte antigen alleles [50]. AA dermal fibroblasts also express and secrete higher levels of TGFβ1 at baseline and respond much more robustly to exogenous TGFβ1 in elastin production than fibroblasts from Caucasian skin biopsies [390]. Recombinant HGF selectively abolishes connective tissue growth factor (CTGF) expression and collagen accumulation, effects mediated through cMET phosphorylation in lung fibroblasts isolated from Caucasian but not from AA patients with systemic sclerosis (SSc) [397].

Monocytes from AAs and from patients with SSc have low CAV1 levels, which causes enhanced migration towards C-X-C motif chemokine receptor (CXCR)4 ligand stromal cell-derived factor (SDF-1) and enhanced differentiation to fibroblasts [398]. However, monocytes from AA donors have limited extracellular receptor kinase (ERK) activation and no JNK, p38, or Smad2/3 activation, but significantly activate ERK in response to SDF-1, in contrast to high-level activation of these pathways without further SDF-1 induced enhancement in SSc patients [398].

Keloid formation in AAs is associated with a higher occurrence of SNPs in myosin genes 1E (MYO1E) and MYO7A in studies using admixture mapping and whole-exome associations [394]. Growing fibroblasts cultured from keloids demonstrate glucocorticoid resistance, decreased expression of the Wnt inhibitor SFRP1, SFRP2, MMP-3 and dermatopontin (DPT), and increased expression of insulin-like growth factor binding protein 5 (IGFBP5) and jagged 1 (JAG1), likely controlled by altered epigenetic programming with an altered pattern of DNA methylation and histone acetylation [399].

There is a highly significant association between the -656 minor T allele of SERPINH1 heat-shock protein 47, a chaperone essential for collagen synthesis in fibroblasts, and premature rupture of membranes, which occurs at a significantly greater frequency in AAs than in Caucasians [400]. The SERPINH1 -656 minor T allele displays significantly reduced promoter activity compared to the major -656 C allele in amnion fibroblasts responsible for laying down the fibrillar collagen that gives tensile strength to the amnion, and also occurs at a significantly greater frequency in AAs [400].

Dermal fibroblasts in AA newborns of obese mothers have a relatively inefficient mitochondrial function and increased oxidative stress compared to newborns of lean mothers [401]. This is evidenced by significantly higher mitochondrial respiration without an increase in ATP production, higher levels of ROS, and altered expression of genes involved in fatty acid and glucose metabolism and mitochondrial respiration in fibroblasts from newborns of obese mothers [401].

Studies of CAFs from tumors from AA patients also demonstrate differences. Laser capture microdissected stromal cell amplified mRNA from fresh frozen BC samples from 18 AA and 17 European ancestry patients analyzed by Affymetrix gene arrays demonstrated increased expression of genes regulating translational initiation, RNA processing, angiogenesis, and blood vessel development, and response to DNA damage and endogenous stimuli [378]. The greatest change, by more than a factor of two, in the expression of specific genes in AA patients compared with European patient stroma was observed in phosphoserine phosphatase-like (PSPHL) and CXCL10 in both ER^−^ and ER^+^ tumor stroma and in CXCL11 and in soluble carrier family 38, member 1 (SLC38L1) in ER^+^ stroma [378].

Beta-crystalline B2 (CRYbetaB2) is upregulated in AA breast tumors and promotes de-differentiation, an increase in mesenchymal markers, cancer-associated fibroblasts, and enlargement of nucleoli [402]. CRYbetaB2 induces and directly interacts with nucleoli, leading to the activation of AKT and EGFR signaling and an aggressive phenotype [402]. Higher levels of nucleolar CRYbetaB2 in primary triple-negative BCs in AA patients correlate with decreased survival [402].

Data suggest that the TME may contribute to racially disparate clinical outcomes in PC [403]. Prostate tumors from AA patients have increased reactive stroma associated with chronic inflammatory infiltrates compared to those from Caucasian patients, with AA CAFs having a greater expression of proinflammatory paracrine mediators, including brain-derived neurotrophic factor (BDNF), chitinase 3-like 1 (CHI3L1), dipeptidyl peptidase 4 (DPPIV), FGF-7, IL-18 binding protein (BP), IL-6, and VEGF than Caucasian CAFs [403]. AA CAFs have greater growth response to androgens, FGF-1, and PDGF, and have higher expression of αSMA, vimentin, and tenascin-C, markers associated with myofibroblast activation, than Caucasian CAFs [403]. The differential expression of PDGF is clinically significant based on a study that demonstrates significant associations between low expression of perivascular PDGFR-alpha and -beta and shorter overall survival in CRC [404].

CM from AA CAFs enhances the proliferation and motility of PC cell lines to a greater extent than CM from Caucasian CAFs [403]. In addition, AA patient-derived prostatic epithelial cell line E006AA responds more aggressively to BDNF ligand than Caucasian tumor-derived LNCaP and C4-2B cells in vitro, and an antagonist to the BDNF tyrosine kinase receptor type 2 (TrkB) reduces the protumorigenic effects AA CAFs to a much greater extent than those of Caucasian CAFs [403]. AA CAFs also enhance the tumorigenicity of E006AA in vivo to a greater extent than Caucasian CAFs [403].

### 3.5. Effects of CAFs on Cancer Cells and the Microenvironment

#### 3.5.1. CAFs Generate the Cancer Microenvironment

Fibroblasts are the predominant cell type in the tumor microenvironment [405]. Primary tumor and normal tissue fibroblasts co-evolve with tumor progression, as they cooperatively express a variety of ECM-remodeling enzymes, structural components of the ECM, exosomes, and soluble factors [75,406,407]. This is exemplified by a ten-day contact co-culture of NFs with tumor cells, where the fibroblasts induce EMT in the tumor, with characteristic loss of E-cadherin, induction of vimentin, tumor growth, and metastatic potential in vivo [77]. This suggests that, in fact, the contact-cultured tumor cells transformed the NFs into tumor-associated fibroblasts, which then exerted their protumorigenic effects [77].

Stromal fibroblasts are primarily responsible for generating the cancer stroma, which consists of mostly ECM proteins, such as fibrillar Type I collagen and non-fibrillar HA, constitute up to 85% of the tumor volume in desmoplastic pancreatic cancers or BCs [13,345,408,409], and influence cancer cell behavior in a number of ways [408,410,411]. The tumor matrix fosters tumor cell invasion into the stroma and migration toward the vasculature [412]. The CAF-mediated desmoplastic reaction, secretion of immunosuppressive cytokines, and production of protumoral ECM are primarily generated by TGFβ signaling through F-actin organizing protein paladin isoforms 3 and 4 [413].

Cancer cell invasive potential is suboptimal without the recruitment of CAFs, intense chemical, and physical cross-talk, and interactive remodeling of the ECM [414]. CAFs apply traction forces and deform and remodel ECM by deposition, reorganization, and degradation of fibrous structure, mechanosensing, and stiffening [414]. CAFs remodel stromal collagen I, resulting in stiffer tissue and increased cell–matrix stiffness over that of the softer matrix generated by NFs [101]. A stiff matrix promotes cancer invasion and metastasis [415] and the collapse of blood vessels, which restricts access to therapeutics [230]. In fact, the aging microenvironment may contribute to dormant tumor cell reawakening with stiffening of collagen [416] and secretion of Wnt signaling antagonist SFRP2, reducing response to ROS-induced DNA damage and targeted therapy resistance [417], with endemic fibroblasts losing their capacity to constrain cancer [29].

The tumor matrix modifies cancer behavior through the generation of increased tensile strength that modifies cancer cell signaling [418], lowering oxygen tension, immune cell and therapeutic drug access by stromal pressure on sprouting vessels, generating pH changes [39,411,419] and secreting cytokines [420]. The stiffness of mammalian tissues ranges from 17 kg m^−1^ s^−2^ (Pascals, Pa) for fat, 160 Pa for the normal breast to 2200 Pa in mammary DCIS, 4000 Pa for BC, which ranges from 2000 Pa at the core [421] to 20,000 Pa at the tumor periphery [422], 6000 Pa for pancreatic cancer, 45,000 Pa in thyroid cancer to 310,000 for the Achilles tendon [83,423,424]. Stroma structural proteins increase integrin expression [425] and initiate anti-apoptotic pathways in cancer cells through the binding of integrins [134,137,139,425,426,427], and block the efficacy of drugs such as cisplatin and doxorubicin [428], taxanes [139,427] and flavopiridol [139]. Close proximity to the ECM in tumors increases the survival of cancer cells [425].

Stromal stiffness promotes cancer progression in a variety of solid tumors through mechanical stress on the nuclei and DNA damage during invasion and endothelial transmigration, resulting in genomic instability and cancer progression [83,429,430] (expertly reviewed by Fuller, at al. (2022) [83]. External forces can upregulate transcription factors β-catenin, Hif1α, Twist, Snail, Smad2/3, STAT3, AP-1, c-Myb, Lef1, MRTFA, p53, p300ZNF217 and Yorkie-homologues YAP and/or TAZ, downregulate SRY-related HMG-box (SOX)2 and Nanog, and variably regulate NFκb by tumor type [424], leading to cancer progression. Matrix stiffness also induces stemness in melanoma, hepatocellular carcinoma, glioma, NSCLC and CRC, while it can decrease stemness in some hepatocellular carcinoma (HCC) cell lines, in an intensity-dependent manner, with an optimum stiffness of 5000 Pa in BC, 25,000 Pa in CRC and gastric cancer and 50,000 Pa in osteosarcoma [424].

#### 3.5.2. Effects of CAFs on Cancer Cells

CAFs can affect cancer cell behavior in a dual manner, by both promoting protumorigenic effects, and attempting to inhibit cancer progression (Table 3). The duality has been described and studied by many groups and has been reviewed extensively.

The role of fibroblasts in the tumor microenvironment is one of the most reviewed topics in cancer biology. Fibroblasts have been called spears and shields [14], foes and friends [431], seeds fertilizing the soil [432], partners-in-crime [432], cockroaches [13], maestros orchestrating tissue regeneration [54], protecting angels and wicked witches [433], diabolic liaisons [434], two sides of the same coin, in the case of MSCs and fibroblasts [53], the dark side of the coin, in the case of CAFs [435], drivers or passengers, in the case of fibrous stroma [436], and plying the double-edged sword of fibrosis [437]. This anthropomorphized turning of the agent or tool, which is the fibroblast guarding the host against the insurgent aspirations of cancer, has implicitly been covered as well by the countless articles on cancer cell fusion with mesenchymal stem cells, exosomes, miRNA transfers, cytokine, chemokine and growth factor secretions, immune cell activations, and other mechanisms. The differences in the origins of the heterogeneous tissue fibroblasts that are converted to CAFs may impact whether they are protumorigenic or inhibit cancer progression [438], as do the characteristics of the cancer types with which they interact [439].

Here, I will summarize some of the features that may contribute to the differential effects of CAFs. I will address the salient effects that seem to be central to the epic battle between the cancer and the fibroblast in the tumor microenvironment. I will outline the heroic efforts of the fibroblasts to constrain the cancer and the more resourceful, dominant, atavistic but naïve forces of the cancer to ultimately and inevitably overcome the constraints placed before it, escape the primary site and establish metastases that will eventually doom the host (along with the cancer itself…oops!).

##### Protumorigenic Effects

CAFs lay down and remodel the ECM by generating MMPs, inducing tumor EMT and promoting cancer motility, invasion, and angiogenesis, providing nutrients, inducing inflammation, immune suppression and resistance to drugs and radiation [184,345,438,440,441,442,443,444]. CAFs isolated from human BCs secrete significantly greater amounts of collagen triple helix repeat containing-1 (CTHRC1) than normal fibroblasts, which promotes and is necessary for Wnt/β-catenin signaling pathway-induced migration, invasion, and EMT of BC cells [445]. Adhesion proteins on CAFs also contribute to protumorigenic effects. Melanoma patients with CAFs expressing podoplanin, a mucin-type transmembrane protein, have tumors with greater tumor thickness, more lymph node metastases and poorer survival than patients with podoplanin-negative CAFs [446].

Tumor cells occupy a dynamic plastic range spanning the epithelial to mesenchymal spectrum, which they traverse freely as a result of interactions with stromal elements through secreted growth factors and cytokines, other proteins, miRNAs, adhesion signaling, stiffness, pH and hypoxic signaling, exosomes or fusion with stromal cells [50]. Different ligands have functions in driving EMT, including TGFβ, FGFs, EGF, IGF, HGF, and PDGF, which co-ordinate with inflammatory signaling, including IL-1β, NFκb and TNFα [50]. Receptors activate transcriptional pro-epithelial regulators including FOXA1, GATA3, or EMT signaling, including SNAIL, SLUG, ZEB, and Twist1, to achieve the plasticity needed to transition from the invasive mesenchymal state to the epithelial state to allow colonization of the primary and the metastatic sites [50].

The activation of NFs to CAFs endows them with multiple tumor-promoting functions. They play key roles in drug resistance, ECM remodeling, produce growth factors, cytokines, and chemokines, and modulate metabolism and angiogenesis, modulate immune cell activity through T-lymphocytes and other immune cells to suppress anti-tumor immune response [447]. CM from NSCLC CAFs induces EMT, acquisition of cancer stem cell-like qualities in vitro, and enhanced tumor formation in vivo in lung cancer cell lines, mediated through an increased expression of TGFβ1, as compared to NFs [80]. CAFs induce EMT in gastric cancer cells through activation of the ERK1/2-SP1- zinc finger E-box binding homeobox (ZEB)2 pathway in an IL-33- and its receptor ST2L L-dependent manner [448].

Mechanistically, CAFs induce activation of TGFβ/SMAD4 signaling and enhance colorectal cancer aggressiveness by reducing fibronectin leucine-rich transmembrane protein 3 (FLRT3) expression, an inhibitor of EMT, proliferation, migration, invasion, and enhancer of apoptosis [449]. CM from human breast tissue CAFs expressing high levels of αSMA and SDF1/CXCL12 induces enhanced cell–extracellular matrix adhesion, migration, invasion and EMT through TGFβ1/Smad signaling [442]. Triple-negative BC-derived CAFs induce CXCL12-CXCR4-mediated recruitment and reprogramming of blood monocytes towards immune suppressive stabilin (STAB)1^+^ translocation associated membrane protein (TREM)2 high lipid-associated macrophages, which inhibit T-cell activation and proliferation and support an immunosuppressive microenvironment [450]. In endometrial carcinoma, CAFs promote cancer progression via the SDF-1α/CXCR4 axis, through PI3K/AKT and MAPK/ERK signaling in a paracrine-dependent manner, and increase MMP-2 and MMP-9 secretion in an autocrine-dependent manner [451].

IL-17a secretion by CAFs markedly enhances tumorigenic effects of gastric cancer through the JAK2/STAT3 pathway in the gastric cancer cells in vitro, with CAFs acting as an independent risk factor for DFS and DSS, as noted above [215]. Wnt/β-catenin signaling is necessary to maintain CAF function and promote tumor progression [287]. IL-1α in bladder cancer cell CM induces the release of IL-8, HGF, MMP-2, GM-CSF and monocyte chemotactic protein (MCP)-1 by CAFs, which, in turn, reciprocally induce migration and invasion in the tumor cells through MCP and HGF [452]. Stromal HGF also induces protumorigenic effects in small-cell lung cancer in vitro and in vivo [453].

AR expression in PC CAFs provides a good prognosis, but a loss of AR [454] and of fibroblast-dependent androgen activation may be responsible for PC progression [455]. Spontaneously immortalized stromal fibroblasts [456] promote the growth and progression of prostate tumors in mice, and stroma can induce permanent gene expression and behavior changes in epithelial cells [200]. CAFs from tumors co-cultured with cancer cell lines increase cancer cells’ secretion of IL-6 and IL-8, the invasive capacities of MDA-MB-231 cells, and the angiogenic capacities of HUVEC cells [457]. Expression of MMPs by cancer-associated fibroblasts and mononuclear inflammatory cells is associated with a worse prognosis in BC [458], and tumor-associated CD163^+^ and CD68^+^ macrophages (TAMs) in the tumor stroma are associated with higher stage and invasiveness in BC [459].

High stroma-tumor ratios indicate an independent risk factor for a worse prognosis and resistance to treatment in aggressive breast, pancreatic, colon and other cancer types [372,460,461,462,463,464], with concurrent loss of Smad4 staining contributing to classifying patients to a worse prognostic group [460]. However, the effects of CAFs on cancer progression are not uniform [372], and higher stromal content may be beneficial to prognosis in some situations [465,466], with their depletion potentially inducing more aggressive tumors [467,468]. A gene expression profile of BC stroma, characterized as activated by fibrosis and motility genes, has an unfavorable prognostic effect on survival of ER+ BC patients, reaching statistical significance in hormone-treated patients, in contrast to inactive stroma characterized by expression of claudin and adhesion molecule genes [130]. Malignant stroma also exerts a powerful chemoprotective effect on cancer cells [411,469].

Pancreatic cancers generate their exceptionally malignant traits in part through their characteristic extreme activation of tumor stroma through one of their primary drivers, the production of Sonic Hedgehog (SHH) by both tumor and stroma [470]. Patient survival analysis and immunohistochemistry of surgical samples identified cytidine deaminase (CDA), EGF-like repeats and discoidin domains (EDIL)3, ITGB4, plasminogen activator, urokinase receptor (PLAUR) and SPARC (osteonectin), cwcv- and kazal-like domains proteoglycan (SPOCK)1 as SHH-dependent stromal factors that are associated with poor prognosis in pancreatic cancer patients [470]. SHH-related genetic changes occur in multiple cancers, including 85% of basal cell carcinomas, 87% of medulloblastomas, and less often in breast, colorectal, gastric, pancreatic, non-small-cell lung, and ovarian cancers [471]. SHH signaling in CAFs induces angiogenesis, fibrosis, immune evasion, and neuropathic pain [471]. SHH signals are transduced through patched receptors to the smoothened class frizzled G-coupled receptor (SMO)-SUFU negative regulator of hedgehog signaling (SUFU)-GLI-Kruppel family member GLI1 (GLI) and SMO-Gli-RhoA signaling cascades. The GLI transcription network upregulates target genes, such as *BCL2*, *FOXA2*, *FOXE1*, *FOXF1*, *FOXL1*, *FOXM1*, *GLI1*, *HH interacting protein (HHIP)*, *patched (PTCH)1*, and *WNT2B*, in a cell context-dependent manner [471]. In triple-negative BC models, SHH ligand produced by cancer cells reprograms CAFs to generate a supportive niche to acquire a chemo-resistant, cancer stem cell phenotype through FGF-5 expression and production of fibrillar collagen [472]. Treatment of patient-derived xenografts in a murine model with SMO inhibitors reverses the expression of cancer stem cell markers and sensitizes tumors to docetaxel chemotherapy, prolonging survival and decreasing metastases [472]. However, as discussed below, due to their heterogeneity, some pancreatic stromal elements can also act to restrain cancer growth.

The Wnt, Hippo, TGFβ, and MAPK pathways are deregulated in both CAFs and melanoma cells in the tumor microenvironment [473]. All four pathways exert a protumorigenic effect in melanomas, but only Wnt, TGFβ, and MAPK clearly support a protumorigenic effect by CAFs and may be candidates for therapeutic targets in CAF tumorigenic function [473]. However, Hippo signaling in CAFs may induce tumor suppression and may have a role in metastatic regression, raising caution in its use as a target in therapy [473].

CAFs converted by co-culture of BM MSCs with esophageal carcinoma cells have high expression of FAP, which is associated with poor prognosis of esophageal squamous cell carcinomas in patients [474]. CAFs play a tumor-promoting role in esophageal carcinoma through IL-6- and CCL2-promoted tumor migration and TAM-like polarization and invasion of macrophages [474]. These effects are mediated by plasminogen activator inhibitor-1 (PAI-1), activation of AKT and ERK signaling via low-density lipoprotein (LDL) receptor-related protein 1, and PTEN/Akt and MEK/ERK and their downstream targets, NF-kappaB and β-catenin [474]. The CAFs express high levels of metallothionein 2A (MT2A), which is necessary for their tumor-promoting effects. The effects of MT2A are mediated through the expression and secretion of insulin-like growth factor binding protein (IGFBP)2 via NFκB, AKT, and ERK signaling, and high MT2A expression correlates with poor prognosis in esophageal cancer patients [475].

Data support a significant role for epigenetic processes in the transformation of NFs to CAFs [476]. CAFs induce malignant behavior through epigenetic reprogramming, evidenced by enhanced migration of several ovarian cancer cell lines by non-contact co-incubation with CAFs, dependent on an induced increase in the expression of histone methyltransferase and enhancer of zeste homolog (EZH)2 in the cancer cells [477]. CAFs work in conjunction with the cancer cells, undergoing a reciprocal co-evolution and generation of an inflammatory microenvironment [432]. Breast CAF pro-invasive activity is sustained by the constitutive activation of STAT3 and DNA methyltransferase (DNMT)3B, Janus kinase 1/signal transducer and activator of transcription 3 (JAK1/STAT3), and hypomethylation of tumor suppressor genes TGFβR2, THY1, and PTEN in CAFs [478]. CAFs that undergo epigenetic modification have increased proliferation, change the microenvironment composition, inhibit apoptosis, prevent cytotoxin penetration, and induce treatment resistance in vivo in pancreatic cancer [479,480,481].

CM from CAFs from HNSCC patient tumors increase HNSCC cell migration, invasion, and proliferation in vitro and CAFS increase tumor growth and metastasis in vivo in a murine floor of mouth tumor model [482]. HNSCC CAFs fall into two groups, depending on the presence or absence of a well-demarcated boundary between epithelial cancer cells and the surrounding ECM [483]. The CAFs from non-demarcated boundary tumors stimulate matrigel invasion and tumorigenicity in a mouse tumor transplant model, in sharp contrast to CAFs from tumors with demarcated boundaries that have particularly high expression of COL3A1 and COL6A6 in gene expression microarrays [483].

Co-culture of cancer cells with CAFs slows cancer cell circadian cycling periods and metabolic parameters to resemble that of fibroblasts, including glycolytic capacity and reserve, respiration, ATP production, mitochondrial coupling efficiency, and spare respiratory capacity, and results in decreased apoptosis, increased viability and chemoresistance [333]. These changes correspond to increased mRNA levels and expression patterns of core clock genes basic helix-loop-helix ARNT-like 1 (BMAL1), circadian locomotor output cycles kaput protein (CLOCK), cryptochrome circadian regulator 1-2 (CRY1-2), period circadian regulator 1-3 (PER1-3), and clock-controlled genes WEE1 G2 checkpoint kinase (WEE1) and cMYC in cancer cells placed in CAF co-culture or treated with exogenous TNFα, and resulted in increased resistance to chemotherapy [333].

CAFs contribute to the strategies used by cancer cells to interfere with the anti-tumor response of tumor-infiltrating cytotoxic T-cells, which are mainly restricted to stromal zones [484]. CAFs sample, process, and cross-present antigens, killing CD8^+^ T-cells in an antigen-specific, antigen-dependent manner via PD-L2 and fas cell surface death receptor ligand (FASL), whose blocking by checkpoint inhibitors can restore T-cell cytotoxic activity [484].

Additional evidence supporting this role comes from data demonstrating that CRC CAFs have an enhanced capacity to cross-present neoantigens when compared with normal colonic fibroblasts, through a process that involves the lysosomal protease cathepsin S, which is upregulated in fibroblasts by tumor cell CM [485]. Cognate interactions between CD8^+^ T-cells and cross-presenting CAFs suppress T-cell function, with associated decreased cytotoxicity, reduced activation by CD137 expression and increased exhaustion [485]. This is reflected by Hepatitis A virus cellular receptor 2 (HAVCR2, TIM3), lymphocyte activating gene (LAG)3, and ectonucleoside triphosphate diphosphohydrolase 1 (ENTPD1, CD39) marker expression, supporting a role for CAFs in directly suppressing tumor-specific T-cell function in an antigen-dependent fashion [485].

Hence, the effects of CAFs on the entire microenvironment contribute to tumor promotion. The microenvironment can also promote tumor progression in metastasis. Tumor-associated fibroblasts in growing metastatic tumors have different gene expression profiles than primary tumor-associated fibroblasts or normal breast fibroblasts, and in particular, have higher levels of IGF-1 to drive tumor growth and suppress T-cell proliferation [181,486]. CAFs induce tumor cell clusters with epithelial and mesenchymal plasticity at metastatic sites through the production of SDF-1 and TGFβ [487]. Stroma also promotes the cancer cell’s ability to induce osteoclastogenesis [488].

Other effects of CAFs on the microenvironment include educating NFs to generate ROS, induce NFκB-mediated expression of inflammatory cytokines and the ECM protein ASPN, which increases the expression of indoleamine 2,3-dioxygenase 1 (IDO-1), kynureninase (KYNU) and pregnancy-associated plasma protein-A (PAPP-A), without increasing αSMA [489]. The educated fibroblasts are cytocidal to CD8^+^ T-cells, activate cancer cell IGF-1 production, and continue to educate other NFs, in turn [489]. Functionally, ASPN^+^/IDO^+^/KYNU^+^/αSMA^−^ fibroblasts are at the invading front of gastric cancers, expand in the fibrotic stroma and cause cancer cell dissemination [489].

CAFs also enhance monocyte differentiation into M2-like tumor-associated macrophages via secretion of IL-6 and GM-CSF in vitro and increase IL-6 and GM-CSF, infiltrating tumor-associated macrophages and metastases in vivo, effects dependent on these cytokines in an orthotopic mixed CAF–tumor cell transplant [490]. GM-CSF and IL-6 expression also correlates positively with disease course in clinical data analysis [490]. Pancreatic stellate cells in the juxtatumoral compartment also modulate resistance to cellular immunity in pancreatic cancer through the exclusion of FoxP3^+^, CD56^+^, CD20^+^ or CD8^+^ T-cells through their characteristic desmoplastic reaction, and by attracting CD8^+^ T-cells away from tumor cells through secretion of CXCL12 [491]. The desmoplastic T-cell exclusion translates to clinical effects, with distinct pancreatic cancer patient subpopulations with high juxtatumoral densities of CD8^+^ T-cells having longer survival times than patients with low densities [491]. Furthermore, differential fibrosis affects T-cell infiltration differentially in peritoneal metastases than the effect observed in liver and lung metastases of patients with colon cancer [492]. The infiltration of CD3^+^/CD8^+^ tumor-infiltrating lymphocytes into colorectal cancer peritoneal metastases is also prevented by high concentrations of αSMA^+^ CAFs, collagen fibers, and high intratumoral fibrosis, contributing to treatment resistance of peritoneal metastases and a poor prognosis [492]. This is seen in contrast to low levels of CAFs and fibrosis in liver and lung metastases, which have high concentrations of tumor-infiltrating CD3^+^/CD8^+^ T-cells [492].

Tumor fibroblasts and MSCs also affect other cells in the microenvironment. In gastric cancer, associated MSCs prime neutrophils through the secretion of IL-6 and activate neutrophil STAT3 and ERR1/2 pathways, and in turn, neutrophils induce differentiation of normal MSCs into CAFs [493].

##### Tumor-Inhibitory Effects

In contrast to the induction of stemness in cancer cells, stemness marker Notch1 intracellular signaling in CAFs acts as a molecular switch to regulate melanoma cell plasticity [494]. It regulates plasticity by inhibiting the stemness of melanoma stem/tumor-initiating cells, as defined by abrogation of the CD271^+^, Nestin^+^ and spheroid colony-forming phenotype and suppression of Sox2/Oct4/Nanog expression, with concomitant dampening of in vitro and in vivo tumor aggressiveness and metastasis [494]. Stem cell characteristics of fibroblasts in heterogeneous populations appear to either support [495], or suppress cancer progression [75,124,125,496]. CAFs in melanoma have lower Notch pathway activity compared to NFs. Notch expression in CAFs restricts melanoma cell growth in vitro and tumor growth and tumor angiogenesis in vivo [75]. Once again, this is circumstance-specific, and phenotypically opposite data in nasopharyngeal carcinomas, where αSMA^+^ SDF1^+^ CXCR4^+^ CAFs are associated with increased CD133^+^/VEGFR2^+^ cells and increased microvessel density in the stroma surrounding cancer nests suggest induction of neoangiogenesis by CAFs [497].

Cancer-promoting CAFs and cancer-inhibiting CAFs are seen in the same stroma, suggesting that we are observing different aspects of the same fibroblasts depending on experimental conditions, observational contexts, or CAFs with different origins, spatial distributions and gene expression profiles [496]. Differences in CAF effects on different tumor types even translate to patient prognosis. For example, patients with pancreatic ductal carcinoma with αSMA expression in tumor samples have significantly shorter survival and recurrence-free survival than patients without αSMA expression, whereas biliary cancer patients with αSMA expression in tumor samples have better recurrence-free survival than patients without αSMA expression [439]. Functionally, CAF CM and IL-8 secreted into the CM suppress the proliferation of human extrahepatic bile duct carcinoma OCUCh-LM1 cells in an IL-8 receptor CXCR2-dependent manner, but not that of pancreatic ductal cancer cells [439]. Additionally, invoking a cellular context assumes that this factor has an overriding role in CAFs. Due to the heterogeneity of stromal cell populations, SHH signaling in CAFs, which can support tumor promotion, in some CAFs may be responsible as well for tumor differentiation, immune surveillance, T-cell immunity, decreased angiogenesis, and αSMA-expression [467,498,499,500,501,502]. SHH signaling in CAFs inhibits Ras-dependent tumor progression in pancreatic intraepithelial neoplasia and pancreatic cancer [499]. These CAFs are considered tumor repressive, along with other CAFs exhibiting stem cell characteristics, inhibit tumor growth and the effects of regulatory T-cells, and proliferate in response to SHH signaling [496]. Hence, it is possible that CAFs may restrain tumors in their nascent stages but subsequently acquire opposite effects as tumors progress, escape these controls, and further modify CAFs. In another instance, Rho signaling governs the inhibitory effects of fibroblasts on tumor cell growth [110].

Both hepatic stellate cells, the main source of myofibroblastic CAFs, and all other CAFs promote tumor metastatic growth and mortality of desmoplastic CRCs and pancreatic cancer liver metastases through secreted hyaluronan and HGF, but not those of non-desmoplastic tumors [503]. The effect is opposed by myofibroblast CAF-expressed collagen I, which suppresses tumor growth by mechanically restraining tumor spread, overriding its own stiffness-induced mechanosignals [503].

αSMA-depleted CAFs, which express meflin, a glycosylphosphatidylinositol-anchored extracellular protein recognized as a marker for undifferentiated MSCs, suppress tumor progression in pancreatic ductal carcinoma cells [496]. Meflin binds to BMP-7 through its leucine-rich repeats, augments its signaling, inhibits TGFβ signaling and prevents fibrosis by inhibiting collagen crosslinking activity, and physiologically induces stromal softening [496,504]. Genetically induced expression of meflin in CAFs in vivo improves drug delivery to tumors [504].

Expression of inhibitor of nuclear factor kappa B kinase subunit beta (IKKβ)/NFκB has a tumor-suppressive function in CAFs in colitis-associated cancer [502]. IKKβ/NFκB negatively regulates the expression of protumorigenic HGF and of TGFβ through Smad7 and Smurf1 [502].

Human liver CAFs from colon cancer metastases promote a malignant phenotype after the loss of expression of miR-10-5p present in normal liver fibroblasts, which suppresses proliferation, migration, and IL-6 and IL-8 levels in colon cancer cells [505]. miR-206, a suppressor of the tumorigenic phenotype in cholangiocarcinoma cells, is downregulated in NFs when they are co-cultivated with cholangiocarcinoma cells, resulting in their transformation into CAFs [272]. CAFs transformed from NFs by suppression of miR-206 by cholangiocarcinoma cells, in turn reciprocally promote malignant behavior and gemcitabine resistance in cholangiocarinoma [272].

The relevant points that appear to emerge from the totality of the data are that NFs retain cancer suppressive characteristics for short periods in proximity to cancer but lose it relatively quickly once they are activated. Exosomes bearing tumor-suppressive miRNAs are diminished as NFs become converted to CAFs. The positional anatomic source of NFs from within the differentiated organ factors into the ultimate genetic profile of the activated CAF. Acquired genomic instability in the CAFs contributes to their cancer-induced exceptional heterogeneity. Some order in the classification of the heterogeneity of gene expression has been demonstrated but this varies in different studies with different tumor types and also varies among patients with similar tumors and with differential progression within a given tumor. Retention of stem cell characteristics, along with associated signaling pathways in the CAFs provides tumor-suppressive traits. However, stem cell characteristics are minimized in the progressive heterogeneity of the CAF populations that favor trending to tumor-supportive abilities and which are often identified as tumor supportive in the presence of mixed populations. Retained characteristics in some CAFs that support efforts of structural stromal suppression of cancer are countered effectively by the growing corps of activated CAFs exerting opposing effects.

**Table 3:** CAFs regulate cancer cell behavior.

Effects	Mechanisms	References
**Tumor enhancing**		
Generate desmoplastic stroma	High fibrotic stroma-tumor ratio	[372,460,461,462,463,464,489]
	Immunosuppressive cytokines	[413]
	TGFβ singling activation by paladin-mediated F-actin organization	[413]
	Tumor invasion toward vasculature	[412]
	application of traction force and stroma remodeling supporting invasion	[414]
	Increasing stroma stiffness-initiating cancer cell protumorigenic signals, mechanical stress on nuclei, DNA damage, genomic instability	[83,415,418,429]
	Upregulation of transcription factors, EMT and cancer cell stemness	[424]
	Collapse of blood vessels accessing tumors decreases oxygen tension, pH, immune cells, therapy access and angiogenesis	[39,230,411,419]
	Secreting cytokines	[420]
	Initiating anti-apoptotic and treatment resistance signaling through binding of upregulated integrins	[134,137,139,425,426,427,428]
	Aging-induced collagen stiffening, reduced ROS DNA damage response and targeted therapy resistance	[29,416,417]
Secrete growth factors cytokines and chemokines		
	TGFβ and Wnt/β-catenin signaling	[445]
	Podoplanin and surface membrane protein signaling	[446]
	Metabolism	[447]
	Proliferation	[215]
	Angiogenesis	[447,457]
	Induce EMT	[80,287,448]
	Proliferation, migration, invasion, and metastasis	[442,449,451,452,457,474,475,482,489]
	Immune system modulation	[181,447,450,457,459,474,484,485,486,489,490,491,492]
	Loss of CAF androgen receptor expression in prostate cancer	[454,455]
SHH signaling	angiogenesis, fibrosis, immune evasion, and neuropathic pain	[470,471]
Notch1 signaling	Increased cancer cell stemness, microvessel density, neoangiogenesis	[497]
Wnt, Hippo, TGFβ, MAPK signaling	Proliferation, invasion, metastasis	[473]
Epigenetic reprogramming	Migration, inhibited apoptosis, treatment resistance	[477,478,479,480,481]
Slowing of cancer cell circadian cycling periods	Slowing of cancer metabolic parameters of glycolytic capacity, reserve, respiration, ATP production, mitochondrial coupling efficiency, decreased apoptosis, increased viability and chemoresistance	[333]
Prime neutrophils	Activate MSCs to CAFs	[493]
		
**Tumor suppressive**	High stroma–tumor ratio	[465,466,467,468]
	Active extratumoral fibroblasts with high expression of fibrosis and cellular movement genes	[130,469]
Notch1 signaling	Inhibiting stemness	[494]
SHH signaling	Tumor differentiation, immune surveillance, T cell immunity, decreased angiogenesis and αSMA-expression	[467,498,499,500,501]
Increased CAF stem cell characteristics	Inhibiting proliferation and angiogenesis	[75,124,125,495,496]
IL-8	Inhibiting proliferation of some cancer types, not others	[439]
Rho signaling	Inhibiting tumor proliferation	[110]
CAF-expressed collagen I	Suppressing tumor growth by mechanically restraining tumor spread, overriding its own stiffness-induced mechanosignals	[503]
Meflin-expressing αSMA-depleted CAFs	Inhibiting TGFβ-induced fibrosis through inhibition of collagen crosslinking	[496,504]
IKKβ/NFκB	Negatively regulates HGF and TGFβ expression, inhibits tumor growth, promotes tumor cell death and suppresses regulatory T cells	[502]
miR-10-5p, miR-206	Suppressing proliferation, migration, cytokine levels	[272,505]

## 4. Cancer Stroma as a Therapeutic Target

Therapeutic approaches to CAFs and MSCs are expertly reviewed elsewhere [25,26,46,352,506,507,508]. A significant body of literature discusses the potential of targeting cancer stroma, and specifically CAFs. Most of the studies address in vitro approaches to the unique characteristics of CAFs that they acquire through genetic, epigenetic, and cellular transfer mechanisms outlined above, with a goal of eliminating them or reverting them to deactivated fibroblasts that no longer support tumor progression. Some investigations have addressed the potential of either eliminating CAFs from in vivo tumors in animal models or targeting their protumorigenic characteristics. A number of clinical trials have attempted to translate successes in pre-clinical models, with variable and modest successes. The following is a review of selected relevant pre-clinical investigations and clinical trials that are ongoing or that have reported outcomes.

### 4.1. In Vitro Investigations

Chemotherapy, biotherapy, or radiation therapy can, in some cases, reverse the protumorigenic effects of CAFs [25,26,46,352,506,507,508]. Disruption of growth factor signaling was an approach taken in one study, where antisense oligonucleotides targeting HGF, which was secreted by CAFs, inhibited VEGF production and CAF-induced increase in clonogenic potential in co-cultured tumor cells [453]. The use of phthoxazolin A, which acts as a small-molecule modulator of tumor–stromal cell interactions, can indirectly suppress PC cancer cell growth through inhibition of IGF-I production by prostate stromal cells [509]. In experiments targeting melanoma, simultaneous inhibition of IL-6 and IL-8 is sufficient to fully inhibit CAF-induced human melanoma cell invasiveness [190].

Targeted inhibitors of signal pathways involved in CAF-mediated tumor promotion demonstrate that inhibiting deregulated Wnt, TGFβ and MAPK pathways in CAFs inhibits their protumorigenic effects [229,473]. Dual Raf plus HGF or MET inhibitory combination therapy is a potential therapeutic strategy for BRaf-mutant melanoma to overcome drug resistance [510]. Targeting CAF-mediated HGF-MET signaling in triple-negative BCs inhibits the tumorigenic activities of HGF-MET in an organotypic model [511]. Inhibition of IL-17a reverses the protumorigenic effects of CAFs in vitro in gastric cancer [215]. Knockdown of MT2A in CAF-like cells suppresses the expression and secretion of IGFBP2 and inhibits the growth, migration, and invasion of esophageal carcinoma cells via NFκB, AKT, and ERK signaling pathways [475].

CAF-promoted angiogenesis is inhibited by atractyloside, a small molecule that targets CAFs in CRC cells [512]. Atractyoloside inhibits angiogenesis through target proteins FGF1, ITGB1, and endothelin receptor type A (EDNRA), and migration through MMP9 and integrin subunit alpha (ITGA)V within the ECM [512].

Biologic modulation of CAFs from patients affects their protumorigenic gene expression profiles and characteristics in vitro [513]. 1,25 dihydroxy-vitamin D_3_ downregulates NRG1, WNT5A, PDGFC, genes associated with proliferation, and increases the expression of NFKBIA, TREM-1, genes involved in immune modulation, effects consistent with the anti-tumor effects of 1,25 dihydroxy-vitamin D_3_ in BC [513]. Moreover, treatment of NFs with 1,25 dihydroxy-vitamin D_3_ induces genes involved in the prevention of apoptosis, detoxification, antibacterial defense, and protection against oxidative stress, supporting a fibroblast-mediated role in the intervention to limit carcinogenesis [513]. Treatment of pancreatic CAFs with all-trans retinoic acid (ATRA) reduces expression of αSMA and FAP, decreases the production of ECM, reduces the expression and secretion of IL-6, and results in CAF-mediated reduction of migration and EMT in the tumor cells [514]. ATRA, in combination with inhibition of HSP47, a collagen-specific molecular chaperone, induces pancreatic stellate cell quiescence, re-educates pancreatic stellate cells, and inhibits ECM hyperplasia, enhancing drug delivery to the tumor in a 3D in vitro model [515].

Incubation of CAFs with Desatinib, a small molecule inhibitor of multiple target kinases, including BCR-ABL and members of the SRC family, partially reverses the CAF phenotype and gene expression profile to that of NFs, and reduces the effects of CAF CM that promote tumor cell proliferation [516]. Other approaches to revert CAF programming to NF programming have been investigated, including epigenetic inhibitors of DNA methyltransferases (DNMTs) with inactivated cytidine analogs that block DNA methylation and degradation through the obstruction of DNMT activities [476]

Radiation therapy induces the secretion of miR-9 by microglia, which inhibits mesenchymal–epithelial transition (MET) in tumor cells by targeting the E-cadherin gene, decreasing cell localization in the brain and inhibiting metastases [517]. This anti-metastatic effect of lung cancer cells on the brain contrasts the effects of miR-9 in BC CAFs that promote BC tumor growth, discussed above [269].

As noted above, the pro-inflammatory environment of the stroma induces a deficiency in base excision repair in NFs, generating unrepaired DNA strand breaks and an ATF-4-induced reprogramming into CAFs [232]. Base excision repair deficient CAFs are selectively eliminated by midostaurin, a derivative of staurosporine multi-kinase inhibitors able to interfere with essential CAF functions, such as Ca^++^ signaling [232]. Midostaurin abrogates their tumor proliferative and pro-migratory effects in vitro [232].

Drug resistance in carcinomas is aided by the structural proteins in the microenvironment. Binding to integrins by structural proteins [134,137,139,426,427] and protection by stroma at primary and metastatic sites [134,518] initiates survival signaling, and functional and structural changes in the cancer cells, making them less responsive to cytotoxic therapy. Stromal proteins also pose structural and stiffness-mediated diffusion barriers to drug access [28]. They have been considered potential therapeutic targets themselves and some have already progressed to testing in clinical trials [28]. Another approach to eliminating the tumor-supportive effects of CAFs is through synthetic disruption of structural proteins. Treatment with preoperative systemic chemotherapy for BC has mixed effects on ECM structural proteins [519]. The expression of collagen IV and Tenascin-C is upregulated, syndecan-1 is downregulated, and SMA remains unchanged in CAFs in progressive disease (519). Losartan inhibits collagen I synthesis by hepatic stellate cells, attenuates the compactness of multicellular hepatic tumor spheroids, and reverses their therapeutic resistance to sorafenib and cisplatin [520].

The CAF-induced upregulation of HA synthesis-promoted migration is inhibited by 4-methylumbelliferone (4-MU), a potent HA synthesis inhibitor, suggesting a potential therapeutic role [224]. Analogously, targeting CD44, the ligand of HA, with a small molecule Oncofid-P20 (Onco-P20) reduces growth factor production, downmodulates the Wnt signaling pathway, dampens the marked pro-inflammatory profile of melanoma CAFs and reduces cancer cell proliferation [521].

Human MSC-conditioned medium can arrest human HCC and lung cancer cell line proliferation and migration in vitro, promote apoptosis through downregulation of BCL2, pro-caspase-7, β-catenin, and c-Myc, and upregulate dormancy marker ephrin receptor EphA5 and the spontaneous fusion of MSC with cancer cells [522]. These and other studies [523] suggest that MSCs may have a therapeutic potential in cancer.

### 4.2. In Vivo Investigations

Depletion of FAP-expressing cells in lung and pancreatic carcinomas in mouse tumors causes rapid hypoxic necrosis of cancer and stromal cells in these immunogenic tumors, mediated by interferon-γ and TNFα [524]. Elimination of FAP-expressing cells modulates immune suppression of growth, supporting their roles as nonredundant, immune-suppressive components of the tumor microenvironment [524]. An FAP-directed vaccine suppresses primary and metastatic colon and breast tumor growth, decreases collagen I in tumors, and increases chemotherapy uptake by tumors by 70% [525]. The vaccine prolongs average survival three-fold and results in complete rejection of tumor cell challenges in a murine model, suggesting that it may function as a potential model for combined immune and chemotherapy [525].

Adipose stromal cells recruited to triple-negative BCs are converted to CAFs and in turn, induce tumor growth, EMT, invasiveness, metastasis, and chemotherapy resistance in triple-negative BC cells [526]. D-CAN, a pro-apoptotic modified circular peptide of amino acid sequence CSWKYWFGEC targets adipose stromal cells and suppresses spontaneous BC lung metastases in a mouse allograft model when combined with cisplatin and suppresses lung metastases by itself in a human BC xenograft model [526].

Glucagon-like peptide-1 receptor agonist exendin-4-treatment inactivates pancreatic stellate cells, suppresses their proliferation and migration, and attenuates NFκB-dependent SDF-1 secretion [527]. Pancreatic cancer cells incubated with a conditioned medium from exendin-4-treated stellate cells have a decreased ability to proliferate, migrate, and invade in vitro, similar to effects induced by CXCR4 inhibitor AMD3100 [527]. In vivo administration of exendin-4 attenuates pancreatic stellate cell function by suppressing the deposition of ECM, attenuating tumor growth [527].

Pirfenidone, a small molecule developed to reduce fibrosis in idiopathic pulmonary fibrosis through downregulation of the production of growth factors and procollagens I and II, is synergistic when combined with cisplatin treatments of animals with co-implanted CAFs and NSCLC cells, increases cell death in CAFs and inhibits tumor progression [528]. In addition to reducing fibrosis, pirfenidone also reduces the acquisition of CAF-mediated invasive and immune-suppressive capacity of BC cells by reducing the expression of PD-L1 in CAFs and decreasing the secretion of CCL17 and TNFβ [529].

Eliminating CAFs using FAP-directed vaccines and antibody targeting or FAP^+^-redirected T-cell transfer has had some effects in mice [530]. Another approach has been to revert CAFs to NFs as a potential strategy in cancer therapy [476]. Treating CAFs with DNA methyltransferase inhibitors, with ATRA, calcipotriol and minnelide, a prodrug of triptolide, which is an IL-23 inhibitor, restores NF characteristics, reduces markers of inflammation and proliferation, induces quiescence, inhibits cancer invasion by changing the tumor microenvironment composition, and decreases the fibrosis that generates a barrier between the cancer cells and the basement membrane [479,480,481]. These treatments reduce ECM components such as HA and collagen, improve functional vasculature, induce cancer cell apoptosis, and improve cytotoxin penetration, accumulation, response, treatment outcome, and significantly increase survival in a murine model of pancreatic cancer in vivo [479,480,481]. While 1-25 dihydroxy-vitamin D_3_ inhibits CRC [349] and BC cell [531,532] proliferation and differentiation, it also has a significant effect on CAF proliferation and migration and decreases their activation as measured by the ability to contract collagen gels [349]. Evidence from a study of hepatic fibrosis demonstrates that the anti-fibrotic effects of vitamin D receptor ligands are generated by binding of the vitamin D receptor, which then displaces the TGFβ-directed Smad3 occupation of profibrotic target genes and inhibits chromatin remodeling [533]. Indeed, the presence of vitamin D receptors in CRC and their associated gene expression signatures in stromal fibroblasts are positive predictors of outcome [534]. Other compounds able to reverse fibrotic responses, such as JQ1, a small molecule inhibitor of bromodomain-containing protein 4 (BRD4), a member of bromodomain and extraterminal (BET) proteins able to abrogate cytokine-induced activation of HSCs, may also be considered in fibrogenic tumors [533].

Inhibiting SHH and Gli1 signaling with a small molecule inhibitor in hepatic stellate cells that express high levels of SMO receptor and low levels of SHH ligands, in contrast to cancer cells, which express low levels of SMO and elevated SHH, inhibits proliferation in vivo [535]. Specific inhibition of stromal SHH signaling with neutralizing antibodies or small molecule inhibitors inhibits tumor growth in xenograft murine models [536]. Small molecule SHH inhibitor-mediated stromal depletion in a pancreatic cancer murine model administered in combination with gemcitabine produces a transient increase in tumor vascular density and an increase in the intratumoral concentration of gemcitabine, leading to transient stabilization of disease [537].

Treatment of tumor-bearing mice with polyethylene glycol (PEG)ylated liposomes loaded with salvianolic acid-B (Dan Phenolic Acid A), a small molecule natural product used in traditional medicine as an antifibrotic, can interfere with CAFs by inhibiting the secretion of TGFβ1, reduce collagen deposition and increase the penetration of nanoparticles into tumors [538]. Treatment with Dan Phenolic Acid A results in high expression of Th1 cytokines and chemokines CXCL9 and CXCL10, recruitment of CD4^+^, CD8^+^ T-cells, and M1 macrophages [538]. Conversely, it induces low expression of Th2 cytokine and chemokine CXCL13, decreases myeloid-derived suppressor cells (MDSCs), Tregs, and M2 macrophages in the tumor area, inhibits tumor metastasis to the lungs, and significantly inhibits tumor growth when combined with docetaxel-loaded PEG-modified liposomes [538].

Neutralizing TGFβ in vivo causes remodeling of CAF dynamics, reduces the frequency and activity of myofibroblasts, promotes the formation of immunomodulatory fibroblasts with a strong response to interferon and the development of productive anti-tumor immunity [539]. Blocking the effects of TGFβ induces greater efficacy of programmed death (PD)1 immunotherapy and provides a rationale for the evaluation of TGFβ and PD1 protein co-blockade in patients [539]. Inactivating CAFs by inhibition of TGFβ in vivo also reduces collagen deposition, induces cell-mediated immune response with high expression of Th1 cytokines and chemokines CXCL9 and CXCL10, the recruitment of CD4^+^, CD8^+^ T-cells, and M1 macrophages to the tumor area [538]. CAF and TGFβ inhibition also reduces the expression of Th2 cytokine and chemokine CXCL13, decreases myeloid-derived suppressor cells, Tregs, and M2 macrophages in the tumor area, and decreases tumor growth and metastasis to the lungs [538]. In a CRC model, joint IL-6 and GM-CSF blockade dramatically reverses TAM infiltration, which generates an immunosuppressive tumor niche, and metastasis in orthotopically co-implanted CAFs and CRC cells in vivo [490]. In another transplanted CRC tumor model, the administration of a chemokine receptor CCR5 inhibitor reduces intratumoral αSMA^+^ CAFs that express EGF, and reduces growth [540]. Administration of a CXCL12 receptor CXCR4 inhibitor AMD3100 to mice with human pancreatic tumors induces rapid T-cell accumulation among cancer cells and acts synergistically with αPD-L1 to reverse immune suppression and resistance to immune therapy to diminish or eliminate cancer cells and their loss of heterogeneity of transformation related protein 53 (TRP53) [541].

Leptin is an important cytokine regulating CAF crosstalk with BC cells and is implicated in breast carcinogenesis [542]. Leptin-activated signaling and leptin-induced expression of target genes are inhibited by the nuclear Farnesoid X Receptor-(FXR), a tumor suppressor, through induction of the suppressor of the cytokine signaling 3 (SOCS3) [542]. A synthetic FXR agonist, GW4064, inhibits the leptin-induced tumor-promoting activities of CAFs and CAF-conditioned medium in BC cells by inhibiting growth, motility, and invasiveness in vitro [542]. GW4064 also inhibits tumor growth of human ER^+^ BC cells co-injected with CAFs in a mouse model in vivo, with tumors exhibiting decreased levels of leptin-regulated proteins and a strong staining intensity for SOCS3 [542].

Approaches to normalizing tumor stroma have been considered with some pre-clinical successes [543]. In one study, blocking IL-8 produced by BM MSCs inhibited angiogenesis and CRC growth in vivo [166]. Using retinoids to normalize cancer and stroma behavior has been a long-term goal. In one study, treatment of mice with a synthetic retinoid in vivo induced the expression of meflin, an inhibitor of TGFβ-induced fibrosis, stiffness, and collagen crosslinking, increased tumor vessel area, drug delivery, and the response of pancreatic carcinoma to chemotherapy [504]. Administration of ATRA to mice in a pancreatic cancer tumor model rendered pancreatic stellate cells quiescent, reversed the secretion of cytokines, chemokines, including CXCL12, which regulate T-cell migration towards the stellate cells and away from the tumor, and of adhesion molecules, and increased the numbers of CD8^+^ T-cells in juxtatumoral compartments [491,530]. The administration of dexamethasone has been investigated in CAF normalization. Dexamethasone reduced the recruitment of myeloid cells responsible for CAF activation and decreased CAF accumulation, collagen deposition and the cancer-promoting microenvironment [544]. Collagenase treatment also augments T-cell mobilization and permits their accessibility to tumor cell nests [545].

Targeting and using exosomes in cancer therapy have been considered in pre-clinical models in vitro and in vivo [184]. The preparation of plasma membrane vesicles for cytoplasm or miRNA replacement therapy has been described and is technically feasible [546]. Exosome-based therapy can either utilize inhibitors to prevent exosome uptake using amiloride or diannexin to enhance T-cell activation [184] or to potentially deliver miRNAs, such as miR-222 [268], miR148b [286], miR-101 [285] or miR-3188 [117] to tumors. miRNA delivery can be achieved either by systemic administration, which takes advantage of their extended half-lives in the circulation to directly target tumor receptors, or by direct intratumoral injection, resulting in some antitumor effects in animals [120,184].

As noted earlier, cancer cells induce transcriptional alterations of p53 in CAFs relative to normal NFs [235,237], including in gastric cancer [547]. Gene therapy with a replicative oncolytic adenovirus-expressing wild-type p53 gene (OBP-702) against CAFs and peritoneal metastases in gastric cancer (GC) resulted in cytotoxicity to both gastric cancer cells and CAFs but not to normal gastric fibroblasts in a murine model [547]. It induced apoptosis and autophagy in CAFs, decreased their secretion of cancer-promoting cytokines, was synergistic with paclitaxel in inhibiting the growth of peritoneal metastases, and decreased CAFs in peritoneal metastases [547].

### 4.3. Theranostics

A significant new approach to cancer treatment involves combining tumor imaging and therapy, termed theranostics, which uses a combination of biomarker imaging with therapy. The field is mostly driven by the development and use of radionuclide-labeled markers, but other approaches have been devised. In CAFs, the membrane-bound protease FAP has become a promising target for molecular imaging with positron emission tomography (PET)/computer-assisted tomography (CT) using radiolabeled FAP inhibitors (FAPIs) [548]. FAP-targeted theranostics appear to be equal or superior to the current oncology practice standard that uses tracer 18-fluorodeoxyglucose (FDG) imaging but benefits from lower backgrounds, stronger uptake, and sharper contrasts in several tumor types [549,550]. An initial Iodine-125 radiolabeled FAPI, ^125^I-FAPI-01, targeting human and murine FAP-expressing cells rapidly internalized tracer into fibroblasts, but experienced rapid deiodination and short retention times, limiting the therapeutic efficacy potential [551]. Investigations improved on the early class of agents. In a retrospective analysis, Gallium-68-labeled FAPI ^68^Ga-FAPI-04, an alternate version of the inhibitor, was able to identify 28 different cancer types in 80 patients with 54 primary tumors and 229 metastases, some as small as one cm in diameter, with the highest SUV found in sarcoma, esophageal, breast, cholangiocarcinoma, and lung cancer, with no adverse events reported [552]. Subsequent quinolone-based radiotracer modifications on the original compounds have been developed, which have higher uptake and retention in patients [553]. Specifically, two radiotracers, FAPI-21 and -46, displayed substantially improved ratios of tumor to blood, liver, muscle, and intestinal uptake [553]. Other improved radiolabeled FAP-binding proteins, including dimers, have demonstrated superior preclinical antitumor efficacy [554]. A Technetium-99m-labeled FAP ligand ^99m^Tc-FL-L3 has been developed for imaging triple-negative BC [555]. As noted earlier, fluorescent probes [221] and ^68^Ga-FAPi-46 PET [222] have been used to identify FAP CAF subpopulations that modulate tumor aggressiveness. A prospective exploratory imaging clinical trial with ^68^Ga-FAPi-46 PET demonstrated that tracer uptake and biodistribution across multiple cancers strongly correlated with FAP expression of surgically excised tumor and metastasis tissues [222]. Another FAP binding protein, FAP-2286 linked to a radionuclide chelator, along with its metal complexes, demonstrated high affinity to recombinant FAP and cell surface FAP with rapid and persistent biodistributed uptake of radiolabeled versions ^68^Ga-FAP-2286, Indium-111-labeled ^111^In-FAP-2286, and Lutetium-177-labeled ^177^Lu-FAP-2286 in FAP-positive tumors, with good renal clearance and minimal normal tissue uptake [556]. ^177^Lu-FAP-2286 exhibited antitumor activity in FAP-expressing HEK293 tumors and sarcoma patient-derived xenografts, with no significant weight loss and longer tumor retention and suppression than treatment with radiolabeled FAPI-46 in animal studies [556]. Other, ^68^Ga- and ^177^Lu-bound FAP albumin-binding ligands are under development [557]. Of four albumin-binding FAPIs (TE-FAPI-01 to 04) labeled with ^68^Ga, Yttrium-86 (^86^Y), and ^177^Lu, TE-FAPI-04 had the most favorable performance with respect to in vitro stability, binding affinity, in vivo biodistribution, pharmacokinetics, tumor uptake, labeling, and imaging [558].

Investigations of different radionuclides demonstrated that the beta-emitter ^177^Lu-labelled FAPI-46 had relatively slower treatment effects, but ones that lasted longer than those of the alpha-emitter Actinium-225-labeled ^225^Ac-FAPI-46 in a pancreatic cancer xenograft model [559]. A ^177^Lu-labeled bivalent FAPI (ND-bisFAP) demonstrated eight-fold higher binding affinity to FAP-expressing cells, higher specific tumor uptake, and retention in vivo, four-fold higher radiation delivery, greater reduction in tumor growth but comparable median survival in study animals than ^177^Lu-labeled cells expressing monomeric complexing agent dodecane tetraacetic acid (DOTA)-FAPI-04 [560]. That study serves as a reminder that tumor response does not correspond to differences in survival, and that multitargeted approaches need to be considered in devising therapeutic strategies. Novel hetero-bivalent radiolabeled antibodies to FAP and prostate-specific membrane antigen (PSAMA) are being developed, having demonstrated high and specific tumor targeting of FAP and PSMA [561]. They will enable imaging of lesions expressing FAP, PSMA, or both on the tumor cell surface or within the TME for the theranostic management of heterogeneous tumors [561].

As discussed above, tumor hypoxia is a significant contributor to tumor progression and NF conversion to CAFs, and theranostics have begun to take advantage of it. The most common radiotracer used in hypoxia imaging is ^18^F-fluoromisonidazole (FMISO), which can predict the microenvironment of a tumor, including necrosis, vascularization, and permeability [562]. FMISO PET has been successful in distinguishing glioblastomas from lower-grade gliomas, in providing prognostic information, including survival and treatment response information, and in generating a potential strategy to radiation dose escalation to hypoxia-induced radioresistance [562].

Other theranostic approaches are being investigated as well. Complement factor H (FH)-modified 298 nm nanobubbles carrying doxorubicin, which bind to CAF tenascin C, can be used for contrast-enhanced ultrasonic imaging, as well as eradication of CAFs [563]. Core–shell cobalt–gold nanoparticles with spherical morphology (Co@Au NPs) have superparamagnetic properties and are able to generate hyperthermia upon magnetic field stimulation [564]. Cobalt–gold nanoparticles with elliptical morphology (Co@Au NEs) can be optically excited to generate hyperthermia upon photostimulation, and elevate the medium temperature to 45 °C, which causes the detachment of a previously complexed methotrexate molecule from a Co@Au NEs-MTX complex and induces a 90% reduction in colon cancer cell viability in vitro [564]. Both nanomaterials can be used as contrast agents [564].

Fibrogenic fibroblasts are particularly significant contributors to the malignant potential of cancers and targeting and elimination of such fibrogenic populations has become of interest in theranostic investigations [565,566]. In one study, an infrared dye IR-780 was used to eliminate a fibrogenic population of glycolytic fibroblasts to control the cutaneous scarring [567]. Innovative, precise near-infrared-specific FAP targeting for imaging and photothermal therapy is under development [568].

### 4.4. Clinical Trials

A number of drugs of various classes, signaling inhibitors, natural compounds, retinoids, deltanoids, epigenetic modifiers, immunomodulators, radiolabeled antibodies and inhibitors to CAF antigens, inhibitors of leucine-rich repeat superfamily (LCC) members on CAFs, radioimmunotherapy, MMP inhibitors, disruptors of the microenvironment and its signaling, disruptors of CAFs and other fibroblasts, including ones isolated from fibrogenic tumors that impede immune response are all under prior, current and prospective investigations in multiple clinical trials [352,506,565,566,569,570,571,572,573].

A number of clinical trials directed at stromal components are underway either not completed or with results pending [28], including one using ATN161, a small peptide antagonist of integrin α5β1 with carboplatin in glioblastoma, MEDI-522, a humanized monoclonal antibody to αvβ3 with dacarbazine in metastatic melanoma, volociximib, a chimeric monoclonal antibody to integrin α5β1 with gemcitabine in metastatic pancreatic cancer, verteporfin, a benzoporphyrin derivative photosensitizer alone in glioblastoma or in combination with photodynamic therapy in pancreatic cancer [28]. The following represent some additional relevant examples. Over seventy Notch targeting clinical trials are registered, including a Phase III trial of a Notch gamma-secretase inhibitor (GSI) nirogacestat, which addresses tumor cells, CAFs, and endothelial cells supporting tumor progression [574]. A phase I/II clinical trial in patients with pancreatic cancer is underway to convert CAFs to cancer-restraining NFs on the basis of inhibiting meflin, which, as noted above, is an inhibitor of TGFβ signaling, mediated by BMP-7 binding, to induce stromal softening by preventing collagen crosslinking and fibrosis in combination with two chemotherapy agents [575]. C52-halichondrin-B amine (E7130), a novel microtubule dynamics inhibitor able to increase intratumoral CD31-positive endothelial cells and reduce αSMA^+^ CAFs at pharmacologically relevant concentrations and significantly enhance antitumor agents in mouse models is being tested in a clinical trial [576].

Epigenetic drugs such as DNMT inhibitors (DNMTis) and HDAC inhibitors (HDACis) are currently undergoing clinical trials and have demonstrated abilities to reactivate epigenetically inactivated genes [476]. DNMTis (Azacitidine (Vidaza) and 5-Aza-2′-deoxycytidine (Dacogen)) and five HDACis (vorinostat, romidepsin, belinostat, panobinostat, and chidamide) are US Food and Drug Administration (FDA)-approved for hematologic malignancies and are candidates for CAF-directed clinical trials [476].

Other trials test the prognostic significance of stromal characteristics or the use of radiotracers to stratify prognostic features or staging. A prospective randomized clinical trial is on the way to determine the value of adjuvant chemotherapy for stage II CRC using desmoplastic classifications of immature, intermediate, and mature, based on hematoxylin–eosin staining for myxoid stroma and keloid-like collagen features generated by CAFs exclusively in the tumor front during a desmoplastic reaction, to determine if an intermediate/immature desmoplastic reaction is one of four risk factors supporting the advantage of treatment [577]. Use of the radiolabeled FAP inhibitor (FAPi) [^68^Ga]-FAPi-46 PET has demonstrated heterogeneous localization of FAP in tumors in a clinical trial and will be further explored as a pan-cancer imaging biomarker for FAP expression and as a stratification tool for FAP-targeted therapies [222]. Furthermore, another PET reagent, ^68^Ga-FAPI-04 PET is likely more efficient in providing primary gastric cancer diagnosis and staging than ^18^F-FDG PET in data from a clinical trial [578]. In a trial of neoadjuvant chemoradiotherapy in locally advanced oral cavity HNSCC followed by radical surgery, a higher abundance of PD1^+^ CAFs, as well as PD1^+^ cytotoxic T-cells and macrophages are associated with incomplete response [579].

In a multicenter cohort of prostatectomy patients, tumor and stroma samples were analyzed by in situ hybridization in tissue microarrays for the expression of miR-210, a direct responder to and regulator of hypoxia in PC-CAF crosstalk [580]. Results demonstrate a statistically significant association between the overexpression of miR-210 and reduced clinical failure-free survival [580].

Trials have investigated the response of tumor markers to stromal-directed treatment. Hypoxia-directed therapy for pancreatic cancer with a combination of two functionally different chemotherapy agents with a hypoxia-activated prodrug DNA crosslinker bromo-isophosphoramide mustard significantly increased DNA damage, apoptosis, and tumor necrosis, inhibited cell proliferation and reduced stroma density and intratumoral hypoxia, as compared with one chemotherapy agent plus the prodrug or the two chemotherapy agents alone in tumor biopsies [581]. A phase II trial of green tea active compound polyphenon E, which contains a specific mixture of polyphenols administered to men with positive prostate biopsies prior to prostatectomy demonstrated a decrease in HGF and VEGF in prostate CAFs and correlated with a significant reduction in serum PSA, HGF, and VEGF [582].

To date, most of the completed clinical trials, have not resulted in major advances in the survival of patients with difficult-to-treat solid tumors [24,573]. One example with a modicum of success was reported in a meeting abstract, but not published yet in manuscript format [583]. In that phase II trial, the effects of the TGFβ inhibitor LY2157299 in HCC patients with high alpha-fetoprotein (AFP) levels, which correlate with high TGFβ 1 levels, who failed Sorafenib treatment or were ineligible to receive Sorafenib were reported [584]. These patients had a significant reduction in AFP, TGFβ 1, and E-cadherin levels, and a prolongation in the time to progression and overall survival compared with patients who did not have a reduction in AFP levels [583,584].

Results of other completed clinical trials directed at stroma have not been promising. Single-agent FAP inhibitor Val-boroPro (Talabostat) had minimal activity in patients with previously treated metastatic colorectal cancer in a phase II trial, despite significant, but incomplete inhibition of FAP enzymatic activity in the peripheral blood [585]. In a phase II trial of patients with metastatic colorectal cancer, sibrotuzumab, an antibody targeting FAP did not meet the criteria for a minimal response [586]. Cilengitide, a selective αvβ3 and αvβ5 integrin inhibitor with documented antitumor activity as a single agent in recurrent glioblastoma and in combination with standard temozolomide chemoradiotherapy in newly diagnosed glioblastoma, did not improve outcomes when combined with temozolomide chemoradiotherapy in patients with newly diagnosed glioblastoma with methylated MGMT promoter in a randomized phase III trial, and will not be developed further as an anticancer drug [587]. Trials adding simituzumab, a monoclonal antibody that inhibits the extracellular matrix-remodeling enzyme lysyl oxidase-like 2 that maintains pathological stroma in tumors, to gemcitabine in pancreatic cancer [588] and to 5-fluorouracil, leucovorin, and irinotecan (FOLFIRI) [589] have been completed with negative results with respect to clinical outcomes compared to the chemotherapy regimens alone.

Some trials had modest successes. Chemokines have been targeted in a number of clinical trials in multiple tumor types to determine immune modulation to reduce desmoplasia and increase T-cell infiltration of tumors as demonstrated in preclinical models with variable results [569,590]. Their safety and early efficacy have been tested and have shown tolerability and some promising responses alone or in combination with chemotherapy and radiation [565,569,590]. A phase I study of eribulin plus balixafortide, a CXCR4 inhibitor, demonstrated a 30% objective response rate in heavily pretreated, relapsed metastatic BC patients [591]. Inhibiting desmoplasia with chemokine inhibitors may have more pronounced effects in tumors that are more desmoplastic, such as ER^+^ breast tumors [590]. Clinical trials targeting CAFs, MSCs, and pericytes have had modest successes in prolonging survival, but due to CAF heterogeneity and concerns of opposing effects on cancer progression, global approaches must be abandoned for more specific targeted approaches that perhaps address cancer cell and CAF survival mechanisms in concert [508]. A systematic review and meta-analysis of twenty clinical studies and six randomized controlled trials of anti-VEGF/VEGFR therapy revealed a treatment advantage of combined therapy with nab-paclitaxel, with the only marginal improvement of objective response rates and progression-free survival, but not overall survival [592].

Several challenges presented by the tumor and its stroma outlined throughout this manuscript pose significant obstacles to stroma-based therapy. One of these obstacles to stromal targeting in clinical trials may be the reality of the heterogeneity of stromal effects on cancer and the potential of some CAFs with stem cell characteristics to restrain tumor progression. Clinical trials inhibiting Hedgehog signaling have yielded moderate results, at best, possibly influenced by roles of SHH signaling in restraining pancreatic cancer cell progression [499]. More recently, as a result of positive preclinical data and divergent downstream signaling through SMO, which supports an immunosuppressive TME, a phase I/II clinical trial has been initiated in basal cell carcinoma patients with the FDA-approved SHH inhibitor vismodegib in combination with the checkpoint inhibitor pembrolizumab [471]. The rationale was based on the fact that combined modality may overcome resistance due to mutations, deletions, amplification and bypass signaling downstream of SHH [471]. In a phase I clinical trial of the SMO inhibitor sonidegib combined with docetaxel chemotherapy in patients with metastatic triple-negative BC, 25% of patients derived clinical benefit and one patient had a complete response, suggesting that in specific circumstances, SHH signal blockade may enable tumors to respond to chemotherapy [472].

Overall, the obstacles in targeting CAFs and related stromal cellular and structural elements are colossal. The heterogeneity of CAFs and the spectrum of gene expression, epigenetic, and structural survival characteristics are practically limitless. Stromal cells continue to be generated, recruited, and activated by the cancer cells and by CAFs themselves, despite being targeted. In addition, while cancer cells rely on stroma for support and generation of optimal growth, invasion, and establishment of optimal metastatic conditions, the cancer cells also grow, expanding their heterogeneity and treatment resistance in parallel to that of CAFs and stroma. The direction in developing CAF- and stromal-directed therapy may be gleaned from the few trials that employed hybrid therapy directed at cancer cells, CAFs, stroma, and immune modulation in concert to achieve some modicum of success in prolonging patient survival.

## 5. Conclusions

All of the cells in the TME affect each other, train each other and support the communal goal in the dynamic reciprocity that is the cancer ecosystem [593]. CAFs originate in the solid tumor microenvironment from NFs, pericytes, resident or recruited MSCs, adipocyte-derived fibroblasts, endothelial cells, epithelial cells, and cells of hematopoietic origin. NF and other CAF precursors are heterogeneous and this heterogeneity dictates, to an extent, the genetic characteristics of the converted CAFs. Cancer cells convert CAF precursors into activated CAFs through a number of mechanisms, including ones mediated through soluble factors, direct contact signaling, exosomes carrying miRNAs and other molecules, and fusion. In turn, activated CAFs have reciprocal effects on the tumor, affecting the microenvironment and the cancer cells themselves. The effects are mostly protumorigenic and utilize a highly varied and extensive list of mechanisms, but there is substantial data supporting efforts by CAFs to suppress cancer progression. These efforts are mostly unsuccessful, due to the heterogeneity of the CAF population, most of which is protumorigenic, and which continues to grow and expand its heterogeneity due to genomic instability and continued generation of CAFs by the cancer cells. Indeed, the malignant tumor is a multidimensional autonomous entity, whose elements co-evolve over time to advance each other’s capacities to adapt to the host’s desperate defense mechanisms, which ultimately turn out to be no match for the singularly focused, cooperative efforts of the ever-adaptable cancer cells, associated fibroblasts, and other supporting cells [594].

Efforts to target CAFs in cancer therapy have had some pre-clinical effects and have even resulted in modest effects in clinical trials. However, most clinical trial results have been disappointing, particularly in regard to prolonging survival. Future trials will need to use combined approaches targeting cancers, stroma, and the immune system to move the field forward.

## References

[1] Lengauer C., Kinzler K.W., Vogelstein B. (1998). Genetic instabilities in human cancers. Nature.

[2] Michor F., Iwasa Y., Vogelstein B., Lengauer C., Nowak M.A. (2005). Can chromosomal instability initiate tumorigenesis?. Semin. Cancer Biol..

[3] Vogelstein B., Papadopoulos N., Velculescu V.E., Zhou S., Diaz L.A., Kinzler K.W. (2013). Cancer genome landscapes. Science.

[4] Zhang Y., Weinberg R.A. (2018). Epithelial-to-mesenchymal transition in cancer: Complexity and opportunities. Front. Med..

[5] Derynck R., Weinberg R.A. (2019). EMT and Cancer: More Than Meets the Eye. Dev. Cell.

[6] Ye X., Weinberg R.A. (2015). Epithelial-Mesenchymal Plasticity: A Central Regulator of Cancer Progression. Trends Cell Biol..

[7] Chaffer C.L., San Juan B.P., Lim E., Weinberg R.A. (2016). EMT, cell plasticity and metastasis. Cancer Metastasis Rev..

[8] Dongre A., Weinberg R. (2018). New insights into the mechanisms of epithelial–mesenchymal transition and implications for cancer. Nat. Rev. Mol. Cell Biol..

[9] Goldie J.H. (2001). Drug resistance in cancer: A perspective. Cancer Metastasis Rev..

[10] Reiter J.G., Baretti M., Gerold J.M., Makohon-Moore A.P., Daud A., Iacobuzio-Donahue C.A., Azad N.S., Kinzler K.W., Nowak M.A., Vogelstein B. (2019). An analysis of genetic heterogeneity in untreated cancers. Nat. Rev. Cancer.

[11] Egeblad M., Nakasone E.S., Werb Z. (2010). Tumors as organs: Complex tissues that interface with the entire organism. Dev. Cell.

[12] Koliaraki V., Prados A., Armaka M., Kollias G. (2020). The mesenchymal context in inflammation, immunity and cancer. Nat. Immunol..

[13] Kalluri R. (2016). The biology and function of fibroblasts in cancer. Nat. Rev. Cancer.

[14] Alkasalias T., Moyano-Galceran L., Arsenian-Henriksson M., Lehti K. (2018). Fibroblasts in the Tumor Microenvironment: Shield or Spear?. Int. J. Mol. Sci..

[15] McAllister S.S., Weinberg R.A. (2014). The tumour-induced systemic environment as a critical regulator of cancer progression and metastasis. Nat. Cell Biol..

[16] Binnewies M., Roberts E.W., Kersten K., Chan V., Fearon D.F., Merad M., Coussens L.M., Gabrilovich D.I., Ostrand-Rosenberg S., Hedrick C.C. (2018). Understanding the tumor immune microenvironment (TIME) for effective therapy. Nat. Med..

[17] Metcalf K.J., Alazzeh A., Werb Z., Weaver V.M. (2021). Leveraging microenvironmental synthetic lethalities to treat cancer. J. Clin. Investig..

[18] Hanahan D., Coussens L.M. (2012). Accessories to the Crime: Functions of Cells Recruited to the Tumor Microenvironment. Cancer Cell.

[19] Granot Z., Fridlender Z.G. (2015). Plasticity beyond cancer cells and the “immunosuppressive switch”. Cancer Res..

[20] Tlsty T.D., Gascard P. (2019). Stromal directives can control cancer. Science.

[21] Sahai E., Astsaturov I., Cukierman E., DeNardo D.G., Egeblad M., Evans R.M., Fearon D., Greten F.R., Hingorani S.R., Hunter T. (2020). A framework for advancing our understanding of cancer-associated fibroblasts. Nat. Rev. Cancer.

[22] Wieder R. (2022). Stromal Co-Cultivation for Modeling Breast Cancer Dormancy in the Bone Marrow. Cancers.

[23] Melzer C., von der Ohe J., Hass R. (2020). Altered Tumor Plasticity after Different Cancer Cell Fusions with MSC. Int. J. Mol. Sci..

[24] Wandmacher A.M., Letsch A., Sebens S. (2021). Challenges and Future Perspectives of Immunotherapy in Pancreatic Cancer. Cancers.

[25] Wu F., Yang J., Liu J., Wang Y., Mu J., Zeng Q., Deng S., Zhou H. (2021). Signaling pathways in cancer-associated fibroblasts and targeted therapy for cancer. Signal. Transduct. Target Ther..

[26] Jenkins B.H., Buckingham J.F., Hanley C.J., Thomas G.J. (2022). Targeting cancer-associated fibroblasts: Challenges, opportunities and future directions. Pharmacol. Ther..

[27] Fridman W.H., Miller I., Sautès-Fridman C., Byrne A.T. (2020). Therapeutic Targeting of the Colorectal Tumor Stroma. Gastroenterology.

[28] Jiang Y., Zhang H., Wang J., Liu Y., Luo T., Hua H. (2022). Targeting extracellular matrix stiffness and mechanotransducers to improve cancer therapy. J. Hematol. Oncol..

[29] LeBleu V.S., Neilson E.G. (2020). Origin and functional heterogeneity of fibroblasts. FASEB J..

[30] Hinz B., Lagares D. (2020). Evasion of apoptosis by myofibroblasts: A hallmark of fibrotic diseases. Nat. Rev. Rheumatol..

[31] Buechler M.B., Turley S.J. (2018). A short field guide to fibroblast function in immunity. Semin. Immunol..

[32] Plikus M.V., Wang X., Sinha S., Forte E., Thompson S.M., Herzog E.L., Driskell R.R., Rosenthal N., Biernaskie J., Horsley V. (2021). Fibroblasts: Origins, definitions, and functions in health and disease. Cell.

[33] Rognoni E., Pisco A.O., Hiratsuka T., Sipilä K.H., Belmonte J.M., Mobasseri S.A., Philippeos C., Dilão R., Watt F.M. (2018). Fibroblast state switching orchestrates dermal maturation and wound healing. Mol. Syst. Biol..

[34] Liebau S., Mahaddalkar P.U., Kestler H.A., Illing A., Seufferlein T., Kleger A. (2013). A Hierarchy in Reprogramming Capacity in Different Tissue Microenvironments: What We Know and What We Need to Know. Stem Cells Dev..

[35] Raab S., Klingenstein M., Liebau S., Linta L. (2014). A Comparative View on Human Somatic Cell Sources for iPSC Generation. Stem Cells Int..

[36] Nefzger C.M., Rossello F.J., Chen J., Liu X., Knaupp A.S., Firas J., Paynter J.M., Pflueger J., Buckberry S., Lim S.M. (2017). Cell Type of Origin Dictates the Route to Pluripotency. Cell Rep..

[37] Sacco A.M., Belviso I., Romano V., Carfora A., Schonauer F., Nurzynska D., Montagnani S., Di Meglio F., Castaldo C. (2019). Diversity of dermal fibroblasts as major determinant of variability in cell reprogramming. J. Cell. Mol. Med..

[38] Hausmann C., Zoschke C., Wolff C., Darvin M.E., Sochorová M., Kováčik A., Wanjiku B., Schumacher F., Tigges J., Kleuser B. (2019). Fibroblast origin shapes tissue homeostasis, epidermal differentiation, and drug uptake. Sci. Rep..

[39] Pradhan R.N., Krishnamurty A.T., Fletcher A.L., Turley S.J., Müller S. (2021). A bird’s eye view of fibroblast heterogeneity: A pan-disease, pan-cancer perspective. Immunol. Rev..

[40] Slack J.M.W. (2008). Origin of Stem Cells in Organogenesis. Science.

[41] Czernik M., Fidanza A., Sardi M., Galli C., Brunetti D., Malatesta D., Della Salda L., Matsukawa K., Ptak G.E., Loi P. (2012). Differentiation potential and GFP labeling of sheep bone marrow-derived mesenchymal stem cells. J. Cell. Biochem..

[42] Dominici M., Le Blanc K., Mueller I., Slaper-Cortenbach I., Marini F.C., Krause D.S., Deans R.J., Keating A., Prockop D.J., Horwitz E.M. (2006). Minimal criteria for defining multipotent mesenchymal stromal cells. The International Society for Cellular Therapy position statement. Cytotherapy.

[43] Almalki S.G., Agrawal D.K. (2016). Key transcription factors in the differentiation of mesenchymal stem cells. Differentiation.

[44] Sheng G. (2015). The developmental basis of mesenchymal stem/stromal cells (MSCs). BMC Dev. Biol..

[45] Li P., Gong Z., Shultz L.D., Ren G. (2019). Mesenchymal stem cells: From regeneration to cancer. Pharmacol. Ther..

[46] Frisbie L., Buckanovich R.J., Coffman L. (2022). Carcinoma-Associated Mesenchymal Stem/Stromal Cells: Architects of the Pro-tumorigenic Tumor Microenvironment. Stem Cells.

[47] Cho K.-A., Park M., Kim Y.-H., Woo S.-Y., Ryu K.-H. (2017). RNA sequencing reveals a transcriptomic portrait of human mesenchymal stem cells from bone marrow, adipose tissue, and palatine tonsils. Sci. Rep..

[48] Hou W., Duan L., Huang C., Li X., Xu X., Qin P., Hong N., Wang D., Jin W. (2021). Cross-Tissue Characterization of Heterogeneities of Mesenchymal Stem Cells and Their Differentiation Potentials. Front. Cell Dev. Biol..

[49] Zhang C., Han X., Liu J., Chen L., Lei Y., Chen K., Si J., Wang T.-Y., Zhou H., Zhao X. (2022). Single-cell Transcriptomic Analysis Reveals the Cellular Heterogeneity of Mesenchymal Stem Cells. Genom. Proteom. Bioinform..

[50] Byun J.S., Park S., Caban A., Jones A., Gardner K. (2018). Linking Race, Cancer Outcomes, and Tissue Repair. Am. J. Pathol..

[51] Micallef L., Vedrenne N., Billet F., Coulomb B., Darby I.A., Desmoulière A. (2012). The myofibroblast, multiple origins for major roles in normal and pathological tissue repair. Fibrogenesis Tissue Repair.

[52] Tai Y., Woods E.L., Dally J., Kong D., Steadman R., Moseley R., Midgley A.C. (2021). Myofibroblasts: Function, Formation, and Scope of Molecular Therapies for Skin Fibrosis. Biomolecules.

[53] Soundararajan M., Kannan S. (2018). Fibroblasts and mesenchymal stem cells: Two sides of the same coin?. J. Cell. Physiol..

[54] Costa-Almeida R., Soares R., Granja P.L. (2017). Fibroblasts as maestros orchestrating tissue regeneration. J. Tissue Eng. Regen. Med..

[55] Denu R.A., Nemcek S., Bloom D.D., Goodrich A.D., Kim J., Mosher D.F., Hematti P. (2016). Fibroblasts and Mesenchymal Stromal/Stem Cells Are Phenotypically Indistinguishable. Acta Haematol..

[56] Rinn J.L., Bondre C., Gladstone H.B., Brown P.O., Chang H.Y. (2006). Anatomic demarcation by positional variation in fibroblast gene expression programs. PLoS Genet..

[57] Chang H.Y., Chi J.-T., Dudoit S., Bondre C., van de Rijn M., Botstein D., Brown P.O. (2002). Diversity, topographic differentiation, and positional memory in human fibroblasts. Proc. Natl. Acad. Sci. USA.

[58] Kabakov L., Nemcovsky C.E., Plasmanik-Chor M., Meir H., Bar D.Z., Weinberg E. (2021). Fibroblasts from the oral masticatory and lining mucosa have different gene expression profiles and proliferation rates. J. Clin. Periodontol..

[59] Foote A.G., Wang Z., Kendziorski C., Thibeault S.L. (2019). Tissue specific human fibroblast differential expression based on RNAsequencing analysis. BMC Genom..

[60] Slany A., Meshcheryakova A., Beer A., Ankersmit H.J., Paulitschke V., Gerner C. (2014). Plasticity of fibroblasts demonstrated by tissue-specific and function-related proteome profiling. Clin. Proteom..

[61] Higuchi Y., Kojima M., Ishii G., Aoyagi K., Sasaki H., Ochiai A. (2015). Gastrointestinal Fibroblasts Have Specialized, Diverse Transcriptional Phenotypes: A Comprehensive Gene Expression Analysis of Human Fibroblasts. PLoS ONE.

[62] Driskell R.R., Watt F.M. (2015). Understanding fibroblast heterogeneity in the skin. Trends Cell Biol..

[63] Saini N., Roberts S.A., Klimczak L.J., Chan K., Grimm S.A., Dai S., Fargo D.C., Boyer J.C., Kaufmann W.K., Taylor J.A. (2016). The Impact of Environmental and Endogenous Damage on Somatic Mutation Load in Human Skin Fibroblasts. PLoS Genet..

[64] Muhl L., Genové G., Leptidis S., Liu J., He L., Mocci G., Sun Y., Gustafsson S., Buyandelger B., Chivukula I.V. (2020). Single-cell analysis uncovers fibroblast heterogeneity and criteria for fibroblast and mural cell identification and discrimination. Nat. Commun..

[65] Rinkevich Y., Walmsley G.G., Hu M.S., Maan Z.N., Newman A.M., Drukker M., Januszyk M., Krampitz G.W., Gurtner G.C., Lorenz H.P. (2015). Skin fibrosis. Identification and isolation of a dermal lineage with intrinsic fibrogenic potential. Science.

[66] Ohlund D., Handly-Santana A., Biffi G., Elyada E., Almeida A.S., Ponz-Sarvise M., Corbo V., Oni T.E., Hearn S.A., Lee E.J. (2017). Distinct populations of inflammatory fibroblasts and myofibroblasts in pancreatic cancer. J. Exp. Med..

[67] Croft A.P., Campos J., Jansen K., Turner J.D., Marshall J., Attar M., Savary L., Wehmeyer C., Naylor A.J., Kemble S. (2019). Distinct fibroblast subsets drive inflammation and damage in arthritis. Nature.

[68] Dominguez C.X., Müller S., Keerthivasan S., Koeppen H., Hung J., Gierke S., Breart B., Foreman O., Bainbridge T.W., Castiglioni A. (2020). Single-Cell RNA Sequencing Reveals Stromal Evolution into LRRC15+ Myofibroblasts as a Determinant of Patient Response to Cancer Immunotherapy. Cancer Discov..

[69] Buechler M.B., Pradhan R.N., Krishnamurty A.T., Cox C., Calviello A.K., Wang A.W., Yang Y.A., Tam L., Caothien R., Roose-Girma M. (2021). Cross-tissue organization of the fibroblast lineage. Nature.

[70] Miyashita N., Saito A. (2021). Organ Specificity and Heterogeneity of Cancer-Associated Fibroblasts in Colorectal Cancer. Int. J. Mol. Sci..

[71] Horie M., Miyashita N., Mikami Y., Noguchi S., Yamauchi Y., Suzukawa M., Fukami T., Ohta K., Asano Y., Sato S. (2018). Tbx4 is involved in the super-enhancer-driven transcriptional programs underlying features specific to lung fibroblasts. Am. J. Physiol.–Lung Cell. Mol. Physiol..

[72] Dvorankova B., Lacina L., Smetana K. (2019). Isolation of Normal Fibroblasts and Their Cancer-Associated Counterparts (CAFs) for Biomedical Research. Methods Mol. Biol..

[73] Xiao L., Hu Q., Peng Y., Zheng K., Zhang T., Yang L., Wang Z., Tang W., Yu J., Xiao Q. (2021). TRAP1 suppresses oral squamous cell carcinoma progression by reducing oxidative phosphorylation metabolism of Cancer-associated fibroblasts. BMC Cancer.

[74] Magnusson K., Appelqvist H., Cieślar-Pobuda A., Wigenius J., Karlsson T., Łos M.J., Kågedal B., Jonasson J., Nilsson K.P.R. (2015). Differential vital staining of normal fibroblasts and melanoma cells by an anionic conjugated polyelectrolyte. Cytom. Part A.

[75] Shao H., Moller M., Cai L., Prokupets R., Yang C., Costa C., Yu K., Le N., Liu Z.-J. (2021). Converting melanoma-associated fibroblasts into a tumor-suppressive phenotype by increasing intracellular Notch1 pathway activity. PLoS ONE.

[76] Yu Z., Zhang J., Zhang Q., Wei S., Shi R., Zhao R., An L., Grose R., Feng D., Wang H. (2022). Single-cell sequencing reveals the heterogeneity and intratumoral crosstalk in human endometrial cancer. Cell Prolif..

[77] Xu W., Hu X., Chen Z., Zheng X., Zhang C., Wang G., Chen Y., Zhou X., Tang X., Luo L. (2014). Normal fibroblasts induce E-cadherin loss and increase lymph node metastasis in gastric cancer. PLoS ONE.

[78] Barron D.A., Rowley D.R. (2012). The reactive stroma microenvironment and prostate cancer progression. Endocr. Relat. Cancer.

[79] Puram S.V., Tirosh I., Parikh A.S., Patel A.P., Yizhak K., Gillespie S., Rodman C., Luo C.L., Mroz E.A., Emerick K.S. (2017). Single-Cell Transcriptomic Analysis of Primary and Metastatic Tumor Ecosystems in Head and Neck Cancer. Cell.

[80] Shintani Y., Abulaiti A., Kimura T., Funaki S., Nakagiri T., Inoue M., Sawabata N., Minami M., Morii E., Okumura M. (2013). Pulmonary Fibroblasts Induce Epithelial Mesenchymal Transition and Some Characteristics of Stem Cells in Non-Small Cell Lung Cancer. Ann. Thorac. Surg..

[81] van Beijnum J.R., Dings R.P., van der Linden E., Zwaans B.M.M., Ramaekers F.C.S., Mayo K.H., Griffioen A.W. (2006). Gene expression of tumor angiogenesis dissected: Specific targeting of colon cancer angiogenic vasculature. Blood.

[82] Griner J.D., Rogers C.J., Zhu M.-J., Du M. (2017). Lysyl oxidase propeptide promotes adipogenesis through inhibition of FGF-2 signaling. Adipocyte.

[83] Fuller B.T., Buczynksi E.M., Beshay P.E., Song J.W. (2022). Molecular sensors for detection of tumor-stroma crosstalk. Adv. Cancer Res..

[84] Bissell M.J., Hines W.C. (2011). Why don’t we get more cancer? A proposed role of the microenvironment in restraining cancer progression. Nat. Med..

[85] Klein G. (2014). Evolutionary aspects of cancer resistance. Semin. Cancer Biol..

[86] Alkasalias T., Flaberg E., Kashuba V., Alexeyenko A., Pavlova T., Savchenko A., Szekely L., Klein G., Guven H. (2014). Inhibition of tumor cell proliferation and motility by fibroblasts is both contact and soluble factor dependent. Proc. Natl. Acad. Sci. USA.

[87] Akhavan A., Griffith O.L., Soroceanu L., Leonoudakis D., Luciani-Torres M.G., Daemen A., Gray J.W., Muschler J.L. (2012). Loss of cell-surface laminin anchoring promotes tumor growth and is associated with poor clinical outcomes. Cancer Res..

[88] Korah R., Das K., Lindy M.E., Hameed M., Wieder R. (2007). Coordinate loss of fibroblast growth factor 2 and laminin 5 expression during neoplastic progression of mammary duct epithelium. Hum. Pathol..

[89] Lebret S., Newgreen D., Waltham M., Price J., Thompson E., Ackland M. (2006). Myoepithelial Molecular Markers in Human Breast Carcinoma PMC42-LA Cells are Induced by Extracellular Matrix and Stromal Cells. Vitr. Cell. Dev. Biol. Anim..

[90] Jones J.L., Shaw J.A., Pringle J.H., Walker R.A. (2003). Primary breast myoepithelial cells exert an invasion-suppressor effect on breast cancer cells via paracrine down-regulation of MMP expression in fibroblasts and tumour cells. J. Pathol..

[91] Duivenvoorden H.M., Rautela J., Edgington-Mitchell L.E., Spurling A., Greening D.W., Nowell C.J., Molloy T.J., Robbins E., Brockwell N.K., Lee C.S. (2017). Myoepithelial cell-specific expression of stefin A as a suppressor of early breast cancer invasion. J. Pathol..

[92] Sirka O.K., Shamir E.R., Ewald A.J. (2018). Myoepithelial cells are a dynamic barrier to epithelial dissemination. J. Cell Biol..

[93] Barsky S.H., Karlin N.J. (2006). Mechanisms of Disease: Breast tumor pathogenesis and the role of the myoepithelial cell. Nat. Clin. Pract. Oncol..

[94] Hildenbrand R., Schaaf A. (2009). The urokinase-system in tumor tissue stroma of the breast and breast cancer cell invasion. Int. J. Oncol..

[95] Stoker M.G.P., Shearer M., O’Neill C. (1966). Growth Inhibition of Polyoma-Transformed Cells by Contact with Static Normal Fibroblasts. J. Cell Sci..

[96] Borek C., Sachs L. (1966). The difference in contact inhibition of cell replication between normal cells and cells transformed by different carcinogens. Proc. Natl. Acad. Sci. USA.

[97] Flaberg E., Markasz L., Petranyi G., Stuber G., Dicső F., Alchihabi N., Oláh È., Csízy I., Józsa T., Andrén O. (2010). High-throughput live-cell imaging reveals differential inhibition of tumor cell proliferation by human fibroblasts. Int. J. Cancer.

[98] Kirk D., Szalay M.F., Kaighn M.E. (1981). Modulation of growth of a human prostatic cancer cell line (PC-3) in agar culture by normal human lung fibroblasts. Cancer Res..

[99] Degeorges A., Tatoud R., Fauvel-Lafeve F., Podgorniak M.-P., Millot G., de Cremoux P., Calvo F. (1996). Stromal cells from human benign prostate hyperplasia produce a growth-inhibitory factor for LNCaP prostate cancer cells, identified as interleukin-6. Int. J. Cancer.

[100] Cheng H.-H., Chu L.-Y., Chiang L.-Y., Chen H.-L., Kuo C.-C., Wu K.K. (2016). Inhibition of cancer cell epithelial mesenchymal transition by normal fibroblasts via production of 5-methoxytryptophan. Oncotarget.

[101] Kaukonen R., Mai A., Georgiadou M., Saari M., De Franceschi N., Betz T., Sihto H., Ventelä S., Elo L., Jokitalo E. (2016). Normal stroma suppresses cancer cell proliferation via mechanosensitive regulation of JMJD1a-mediated transcription. Nat. Commun..

[102] Suhovskih A.V., Kashuba V.I., Klein G., Grigorieva E.V. (2017). Prostate cancer cells specifically reorganize epithelial cell-fibroblast communication through proteoglycan and junction pathways. Cell Adh. Migr..

[103] Samoszuk M., Kanakubo E., Chan J.K. (2005). Degranulating mast cells in fibrotic regions of human tumors and evidence that mast cell heparin interferes with the growth of tumor cells through a mechanism involving fibroblasts. BMC Cancer.

[104] Szabova K., Bizikova I., Mistrik M., Bizik J. (2015). Inflammatory environment created by fibroblast aggregates induces growth arrest and phenotypic shift of human myeloma cells. Neoplasma.

[105] Jurgensmeier J.M., Hofler P., Bauer G. (1994). TGF-beta-induced apoptosis of transformed fibroblasts by normal fibroblasts—Independence of cell-to-cell contact and dependence on reactive oxygen species. Int. J. Oncol..

[106] Bruno R.D., Fleming J.M., George A.L., Boulanger C.A., Schedin P., Smith G.H. (2017). Mammary extracellular matrix directs differentiation of testicular and embryonic stem cells to form functional mammary glands in vivo. Sci. Rep..

[107] Sflomos G., Dormoy V., Metsalu T., Jeitziner R., Battista L., Scabia V., Raffoul W., Delaloye J.-F., Treboux A., Fiche M. (2016). A Preclinical Model for ERα-Positive Breast Cancer Points to the Epithelial Microenvironment as Determinant of Luminal Phenotype and Hormone Response. Cancer Cell.

[108] Madar S., Brosh R., Buganim Y., Ezra O., Goldstein I., Solomon H., Kogan I., Goldfinger N., Klocker H., Rotter V. (2009). Modulated expression of WFDC1 during carcinogenesis and cellular senescence. Carcinogenesis.

[109] Kaler P., Owusu B.Y., Augenlicht L., Klampfer L. (2014). The Role of STAT1 for Crosstalk between Fibroblasts and Colon Cancer Cells. Front. Oncol..

[110] Alkasalias T., Alexeyenko A., Hennig K., Danielsson F., Lebbink R.J., Fielden M., Turunen S.P., Lehti K., Kashuba V., Madapura H.S. (2017). RhoA knockout fibroblasts lose tumor-inhibitory capacity in vitro and promote tumor growth in vivo. Proc. Natl. Acad. Sci. USA.

[111] Alexeyenko A., Alkasalias T., Pavlova T., Szekely L., Kashuba V., Rundqvist H., Wiklund P., Egevad L., Csermely P., Korcsmaros T. (2015). Confrontation of fibroblasts with cancer cells in vitro: Gene network analysis of transcriptome changes and differential capacity to inhibit tumor growth. J. Exp. Clin. Cancer Res..

[112] Liu T., Zhou L., Yang K., Iwasawa K., Kadekaro A.L., Takebe T., Andl T., Zhang Y. (2019). The β-catenin/YAP signaling axis is a key regulator of melanoma-associated fibroblasts. Signal. Transduct. Target Ther..

[113] Zhou L., Yang K., Randall Wickett R., Zhang Y. (2016). Dermal fibroblasts induce cell cycle arrest and block epithelial-mesenchymal transition to inhibit the early stage melanoma development. Cancer Med..

[114] Stock C., Jungmann O., Seidler D.G. (2011). Decorin and chondroitin-6 sulfate inhibit B16V melanoma cell migration and invasion by cellular acidification. J. Cell. Physiol..

[115] Kruger S., Abd Elmageed Z.Y., Hawke D.H., Wörner P.M., Jansen D.A., Abdel-Mageed A.B., Alt E.U., Izadpanah R. (2014). Molecular characterization of exosome-like vesicles from breast cancer cells. BMC Cancer.

[116] Shelton M., Anene C.A., Nsengimana J., Roberts W., Newton-Bishop J., Boyne J.R. (2021). The role of CAF derived exosomal microRNAs in the tumour microenvironment of melanoma. Biochim. Biophys. Acta–Rev. Cancer.

[117] Wang X., Qin X., Yan M., Shi J., Xu Q., Li Z., Yang W., Zhang J., Chen W. (2019). Loss of exosomal miR-3188 in cancer-associated fibroblasts contributes to HNC progression. J. Exp. Clin. Cancer Res..

[118] Zhang Y., Cai H., Chen S., Sun D., Zhang D., He Y. (2019). Exosomal transfer of miR-124 inhibits normal fibroblasts to cancer-associated fibroblasts transition by targeting sphingosine kinase 1 in ovarian cancer. J. Cell. Biochem..

[119] Tao S., Li H., Ma X., Ma Y., He J., Gao Y., Li J. (2020). Elevating microRNA-1-3p shuttled by cancer-associated fibroblasts-derived extracellular vesicles suppresses breast cancer progression and metastasis by inhibiting GLIS1. Cancer Gene Ther..

[120] Li Y.-Y., Tao Y.-W., Gao S., Li P., Zheng J.-M., Zhang S.-E., Liang J., Zhang Y. (2018). Cancer-associated fibroblasts contribute to oral cancer cells proliferation and metastasis via exosome-mediated paracrine miR-34a-5p. EBioMedicine.

[121] Rajthala S., Min A., Parajuli H., Debnath K.C., Ljøkjel B., Hoven K.M., Kvalheim A., Lybak S., Neppelberg E., Vintermyr O.K. (2021). Profiling and Functional Analysis of microRNA Deregulation in Cancer-Associated Fibroblasts in Oral Squamous Cell Carcinoma Depicts an Anti-Invasive Role of microRNA-204 via Regulation of Their Motility. Int. J. Mol. Sci..

[122] Ishikawa Y., Onoyama I., Nakayama K.I., Nakayama K. (2008). Notch-dependent cell cycle arrest and apoptosis in mouse embryonic fibroblasts lacking Fbxw7. Oncogene.

[123] Liu Z.-J., Li Y., Tan Y., Xiao M., Zhang J., Radtke F., Velazquez O.C. (2012). Inhibition of fibroblast growth by Notch1 signaling is mediated by induction of Wnt11-dependent WISP-1. PLoS ONE.

[124] Shao H., Cai L., Grichnik J.M., Livingstone A.S., Velazquez O.C., Liu Z.J. (2011). Activation of Notch1 signaling in stromal fibroblasts inhibits melanoma growth by upregulating WISP-1. Oncogene.

[125] Sizemore G.M., Balakrishnan S., Hammer A.M., Thies K.A., Trimboli A.J., Wallace J.A., Sizemore S.T., Kladney R.D., Woelke S.A., Yu L. (2017). Stromal PTEN inhibits the expansion of mammary epithelial stem cells through Jagged-1. Oncogene.

[126] Cheng H.-H., Kuo C.-C., Yan J.-L., Chen H.-L., Lin W.-C., Wang K.-H., Tsai K.K.C., Guvén H., Flaberg E., Szekely L. (2012). Control of cyclooxygenase-2 expression and tumorigenesis by endogenous 5-methoxytryptophan. Proc. Natl. Acad. Sci. USA.

[127] Cheng H.-H., Wang K.-H., Chu L.-y., Chang T.-C., Kuo C.-C., Wu K.K. (2014). Quiescent and proliferative fibroblasts exhibit differential p300 HAT activation through control of 5-methoxytryptophan production. PLoS ONE.

[128] Kawada M., Ishizuka M., Takeuchi T. (1999). Enhancement of antiproliferative effects of interleukin-1beta and tumor necrosis factor-alpha on human prostate cancer LNCaP cells by coculture with normal fibroblasts through secreted interleukin-6. Jpn. J. Cancer Res..

[129] Sakaguchi M., Kataoka K., Abarzua F., Tanimoto R., Watanabe M., Murata H., Than S.S., Kurose K., Kashiwakura Y., Ochiai K. (2009). Overexpression of REIC/Dkk-3 in normal fibroblasts suppresses tumor growth via induction of interleukin-7. J. Biol. Chem..

[130] Román-Pérez E., Casbas-Hernández P., Pirone J.R., Rein J., Carey L.A., Lubet R.A., Mani S.A., Amos K.D., Troester M.A. (2012). Gene expression in extratumoral microenvironment predicts clinical outcome in breast cancer patients. Breast Cancer Res..

[131] Salo S., Bitu C., Merkku K., Nyberg P., Bello I.O., Vuoristo J., Sutinen M., Vähänikkilä H., Costea D.E., Kauppila J.H. (2013). Human bone marrow mesenchymal stem cells induce collagen production and tongue cancer invasion. PLoS ONE.

[132] Delinassios J.G., Hoffman R.M. (2022). The cancer-inhibitory effects of proliferating tumor-residing fibroblasts. Biochim. Biophys. Acta–Rev. Cancer.

[133] Ono M., Kosaka N., Tominaga N., Yoshioka Y., Takeshita F., Takahashi R.-u., Yoshida M., Tsuda H., Tamura K., Ochiya T. (2014). Exosomes from bone marrow mesenchymal stem cells contain a microRNA that promotes dormancy in metastatic breast cancer cells. Sci. Signal..

[134] Korah R., Boots M., Wieder R. (2004). Integrin α5β1 Promotes Survival of Growth-Arrested Breast Cancer Cells. Cancer Res..

[135] Fenig E., Kanfi Y., Wang Q., Beery E., Livnat T., Wasserman L., Lilling G., Yahalom J., Wieder R., Nordenberg J. (2001). Role of transforming growth factor beta in the growth inhibition of human breast cancer cells by basic fibroblast growth factor. Breast Cancer Res. Treat..

[136] Wang H., Rubin M., Fenig E., DeBlasio T., Mendelsohn J., Yahalom J., Wieder R. (1997). Basic FGF causes growth arrest in MCF-7 human breast cancer cells while inducing both mitogenic and inhibitory G_1_ events. Cancer Res..

[137] Barrios J., Wieder R. (2009). Dual FGF-2 and intergrin α5β1 signaling mediate GRAF-induced RhoA inactivation in a model of breast cancer dormancy. Cancer Microenviron..

[138] Tivari S., Lu H., Dasgpta T., De Lorenzo M.S., Wieder R. (2018). Reawakening of dormant estrogen-dependent human breast cancer cells by bone marrow stroma secretory senescence. Cell Commun. Signal..

[139] Najmi S., Korah R., Chandra R., Abdellatif M., Wieder R. (2005). Flavopiridol blocks integrin-mediated survival in dormant breast cancer cells. Clin. Cancer Res..

[140] Simon T., Salhia B. (2022). Cancer-Associated Fibroblast Subpopulations with Diverse and Dynamic Roles in the Tumor Microenvironment. Mol. Cancer Res..

[141] Hamabe-Horiike T., Harada S., Yoshida K., Kinoshita J., Yamaguchi T., Fushida S. (2022). Adipocytes contribute to tumor progression and invasion of peritoneal metastasis by interacting with gastric cancer cells as cancer associated fibroblasts. Cancer Rep..

[142] Shen H., Yu X., Yang F., Zhang Z., Shen J., Sun J., Choksi S., Jitkaew S., Shu Y. (2016). Reprogramming of Normal Fibroblasts into Cancer-Associated Fibroblasts by miRNAs-Mediated CCL2/VEGFA Signaling. PLoS Genet..

[143] Ren G., Zhao X., Wang Y., Zhang X., Chen X., Xu C., Yuan Z.R., Roberts A.I., Zhang L., Zheng B. (2012). CCR2-dependent recruitment of macrophages by tumor-educated mesenchymal stromal cells promotes tumor development and is mimicked by TNFα. Cell Stem Cell.

[144] Ren G., Liu Y., Zhao X., Zhang J., Zheng B., Yuan Z.R., Zhang L., Qu X., Tischfield J.A., Shao C. (2014). Tumor resident mesenchymal stromal cells endow naive stromal cells with tumor-promoting properties. Oncogene.

[145] Coffman L.G., Pearson A.T., Frisbie L.G., Freeman Z., Christie E., Bowtell D.D., Buckanovich R.J. (2019). Ovarian Carcinoma-Associated Mesenchymal Stem Cells Arise from Tissue-Specific Normal Stroma. Stem Cells.

[146] Yang L., Lin P.C. (2017). Mechanisms that drive inflammatory tumor microenvironment, tumor heterogeneity, and metastatic progression. Semin. Cancer Biol..

[147] Gao H., Priebe W., Glod J., Banerjee D. (2009). Activation of signal transducers and activators of transcription 3 and focal adhesion kinase by stromal cell-derived factor 1 is required for migration of human mesenchymal stem cells in response to tumor cell-conditioned medium. Stem Cells.

[148] Jung Y., Kim J.K., Shiozawa Y., Wang J., Mishra A., Joseph J., Berry J.E., McGee S., Lee E., Sun H. (2013). Recruitment of mesenchymal stem cells into prostate tumours promotes metastasis. Nat. Commun..

[149] Landskron G., De la Fuente M., Thuwajit P., Thuwajit C., Hermoso M.A. (2014). Chronic inflammation and cytokines in the tumor microenvironment. J. Immunol. Res..

[150] Raz Y., Cohen N., Shani O., Bell R.E., Novitskiy S.V., Abramovitz L., Levy C., Milyavsky M., Leider-Trejo L., Moses H.L. (2018). Bone marrow-derived fibroblasts are a functionally distinct stromal cell population in breast cancer. J. Exp. Med..

[151] Brennen W.N., Chen S., Denmeade S.R., Isaacs J.T. (2013). Quantification of Mesenchymal Stem Cells (MSCs) at sites of human prostate cancer. Oncotarget.

[152] Fan H., Atiya H.I., Wang Y., Pisanic T.R., Wang T.-H., Shih I.-M., Foy K.K., Frisbie L., Buckanovich R.J., Chomiak A.A. (2020). Epigenomic Reprogramming toward Mesenchymal-Epithelial Transition in Ovarian-Cancer-Associated Mesenchymal Stem Cells Drives Metastasis. Cell Rep..

[153] Viswanathan S., Shi Y., Galipeau J., Krampera M., Leblanc K., Martin I., Nolta J., Phinney D.G., Sensebe L. (2019). Mesenchymal stem versus stromal cells: International Society for Cell & Gene Therapy (ISCT^®^) Mesenchymal Stromal Cell committee position statement on nomenclature. Cytotherapy.

[154] Atiya H., Frisbie L., Pressimone C., Coffman L. (2020). Mesenchymal Stem Cells in the Tumor Microenvironment. Advances in Experimental Medicine and Biology.

[155] Bartosh T.J., Ullah M., Zeitouni S., Beaver J., Prockop D.J. (2016). Cancer cells enter dormancy after cannibalizing mesenchymal stem/stromal cells (MSCs). Proc. Natl. Acad. Sci. USA.

[156] Ren G., Zhang L., Zhao X., Xu G., Zhang Y., Roberts A.I., Zhao R.C., Shi Y. (2008). Mesenchymal Stem Cell-Mediated Immunosuppression Occurs via Concerted Action of Chemokines and Nitric Oxide. Cell Stem Cell.

[157] Biswas S., Mandal G., Roy Chowdhury S., Purohit S., Payne K.K., Anadon C., Gupta A., Swanson P., Yu X., Conejo-Garcia J.R. (2019). Exosomes Produced by Mesenchymal Stem Cells Drive Differentiation of Myeloid Cells into Immunosuppressive M2-Polarized Macrophages in Breast Cancer. J. Immunol..

[158] Cascio S., Chandler C., Zhang L., Sinno S., Gao B., Onkar S., Bruno T.C., Vignali D.A.A., Mahdi H., Osmanbeyoglu H.U. (2021). Cancer-associated MSC drive tumor immune exclusion and resistance to immunotherapy, which can be overcome by Hedgehog inhibition. Sci. Adv..

[159] Moen I., Jevne C., Wang J., Kalland K.-H., Chekenya M., Akslen L.A., Sleire L., Enger P.Ø., Reed R.K., Øyan A.M. (2012). Gene expression in tumor cells and stroma in dsRed 4T1 tumors in eGFP-expressing mice with and without enhanced oxygenation. BMC Cancer.

[160] McLean K., Gong Y., Choi Y., Deng N., Yang K., Bai S., Cabrera L., Keller E., McCauley L., Cho K.R. (2011). Human ovarian carcinoma–associated mesenchymal stem cells regulate cancer stem cells and tumorigenesis via altered BMP production. J. Clin. Investig..

[161] Le Naour A., Prat M., Thibault B., Mével R., Lemaitre L., Leray H., Joubert M.-V., Coulson K., Golzio M., Lefevre L. (2020). Tumor cells educate mesenchymal stromal cells to release chemoprotective and immunomodulatory factors. J. Mol. Cell Biol..

[162] Mishra P.J., Mishra P.J., Humeniuk R., Medina D.J., Alexe G., Mesirov J.P., Ganesan S., Glod J.W., Banerjee D. (2008). Carcinoma-associated fibroblast-like differentiation of human mesenchymal stem cells. Cancer Res..

[163] Blache U., Horton E.R., Xia T., Schoof E.M., Blicher L.H., Schönenberger A., Snedeker J.G., Martin I., Erler J.T., Ehrbar M. (2019). Mesenchymal stromal cell activation by breast cancer secretomes in bioengineered 3D microenvironments. Life Sci. Alliance.

[164] Daverey A., Drain A.P., Kidambi S. (2015). Physical Intimacy of Breast Cancer Cells with Mesenchymal Stem Cells Elicits Trastuzumab Resistance through Src Activation. Sci. Rep..

[165] Forte D., García-Fernández M., Sánchez-Aguilera A., Stavropoulou V., Fielding C., Martín-Pérez D., López J.A., Costa A.S.H., Tronci L., Nikitopoulou E. (2020). Bone Marrow Mesenchymal Stem Cells Support Acute Myeloid Leukemia Bioenergetics and Enhance Antioxidant Defense and Escape from Chemotherapy. Cell Metab..

[166] Wang J., Wang Y., Wang S., Cai J., Shi J., Sui X., Cao Y., Huang W., Chen X., Cai Z. (2015). Bone marrow-derived mesenchymal stem cell-secreted IL-8 promotes the angiogenesis and growth of colorectal cancer. Oncotarget.

[167] Verdelli C., Avagliano L., Creo P., Guarnieri V., Scillitani A., Vicentini L., Steffano G.B., Beretta E., Soldati L., Costa E. (2014). Tumour-associated fibroblasts contribute to neoangiogenesis in human parathyroid neoplasia. Endocr.-Relat. Cancer.

[168] Zhang Y., Bian Y., Wang Y., Wang Y., Duan X., Han Y., Zhang L., Wang F., Gu Z., Qin Z. (2021). HIF-1α is necessary for activation and tumour-promotion effect of cancer-associated fibroblasts in lung cancer. J. Cell. Mol. Med..

[169] Kugeratski F.G., Atkinson S.J., Neilson L.J., Lilla S., Knight J.R.P., Serneels J., Juin A., Ismail S., Bryant D.M., Markert E.K. (2019). Hypoxic cancer-associated fibroblasts increase NCBP2-AS2/HIAR to promote endothelial sprouting through enhanced VEGF signaling. Sci. Signal..

[170] Lee R.H., Yoon N., Reneau J.C., Prockop D.J. (2012). Preactivation of Human MSCs with TNF-α Enhances Tumor-Suppressive Activity. Cell Stem Cell.

[171] Yoon N., Park M.S., Peltier G.C., Lee R.H. (2015). Pre-activated human mesenchymal stromal cells in combination with doxorubicin synergistically enhance tumor-suppressive activity in mice. Cytotherapy.

[172] Galland S., Stamenkovic I. (2020). Mesenchymal stromal cells in cancer: A review of their immunomodulatory functions and dual effects on tumor progression. J. Pathol..

[173] Melzer C., von der Ohe J., Luo T., Hass R. (2021). Spontaneous Fusion of MSC with Breast Cancer Cells Can Generate Tumor Dormancy. Int. J. Mol. Sci..

[174] Dvorak H.F. (2015). Tumors: Wounds that do not heal-redux. Cancer Immunol. Res..

[175] Ridge S.M., Bhattacharyya D., Dervan E., Naicker S.D., Burke A.J., Murphy J.M., O’Leary K., Greene J., Ryan A.E., Sullivan F.J. (2018). Secreted factors from metastatic prostate cancer cells stimulate mesenchymal stem cell transition to a pro-tumourigenic ‘activated’ state that enhances prostate cancer cell migration. Int. J. Cancer.

[176] Zhang T., Lee Y.W., Rui Y.F., Cheng T.Y., Jiang X.H., Li G. (2013). Bone marrow-derived mesenchymal stem cells promote growth and angiogenesis of breast and prostate tumors. Stem Cell Res. Ther..

[177] Ridge S.M., Sullivan F.J., Glynn S.A. (2017). Mesenchymal stem cells: Key players in cancer progression. Mol. Cancer.

[178] Pang T.C.Y., Xu Z., Pothula S., Becker T., Goldstein D., Pirola R.C., Wilson J.S., Apte M.V. (2017). Circulating pancreatic stellate (stromal) cells in pancreatic cancer—A fertile area for novel research. Carcinogenesis.

[179] Erkan M., Reiser-Erkan C., Michalski C.W., Deucker S., Sauliunaite D., Streit S., Esposito I., Friess H., Kleeff J. (2009). Cancer-stellate cell interactions perpetuate the hypoxia-fibrosis cycle in pancreatic ductal adenocarcinoma. Neoplasia.

[180] Apte M.V., Wilson J.S. (2012). Dangerous liaisons: Pancreatic stellate cells and pancreatic cancer cells. J. Gastroenterol. Hepatol..

[181] Del Valle P.R., Milani C., Brentani M.M., Katayama M.L.H., de Lyra E.C., Carraro D.M., Brentani H., Puga R., Lima L.A., Rozenchan P.B. (2014). Transcriptional profile of fibroblasts obtained from the primary site, lymph node and bone marrow of breast cancer patients. Genet. Mol. Biol..

[182] Mueller M.M., Fusenig N.E. (2004). Friends or foes—Bipolar effects of the tumour stroma in cancer. Nat. Rev. Cancer.

[183] Kalluri R., Zeisberg M. (2006). Fibroblasts in cancer. Nat. Rev. Cancer.

[184] Tomasetti M., Lee W., Santarelli L., Neuzil J. (2017). Exosome-derived microRNAs in cancer metabolism: Possible implications in cancer diagnostics and therapy. Exp. Mol. Med..

[185] Rozenchan P.B., Carraro D.M., Brentani H., de Carvalho Mota L.D., Bastos E.P., e Ferreira E.N., Torres C.H., Katayama M.L., Roela R.A., Lyra E.C. (2009). Reciprocal changes in gene expression profiles of cocultured breast epithelial cells and primary fibroblasts. Int. J. Cancer.

[186] Santos R.P., Benvenuti T.T., Honda S.T., Del Valle P.R., Katayama M.L., Brentani H.P., Carraro D.M., Rozenchan P.B., Brentani M.M., de Lyra E.C. (2011). Influence of the interaction between nodal fibroblast and breast cancer cells on gene expression. Tumour Biol..

[187] Pavlides S., Whitaker-Menezes D., Castello-Cros R., Flomenberg N., Witkiewicz A.K., Frank P.G., Casimiro M.C., Wang C., Fortina P., Addya S. (2009). The reverse Warburg effect: Aerobic glycolysis in cancer associated fibroblasts and the tumor stroma. Cell Cycle.

[188] Sotgia F., Whitaker-Menezes D., Martinez-Outschoorn U.E., Flomenberg N., Birbe R.C., Witkiewicz A.K., Howell A., Philp N.J., Pestell R.G., Lisanti M.P. (2012). Mitochondrial metabolism in cancer metastasis: Visualizing tumor cell mitochondria and the “reverse Warburg effect” in positive lymph node tissue. Cell Cycle.

[189] Zimmerlin L., Park T.S., Zambidis E.T., Donnenberg V.S., Donnenberg A.D. (2013). Mesenchymal stem cell secretome and regenerative therapy after cancer. Biochimie.

[190] Jobe N.P., Rösel D., Dvořánková B., Kodet O., Lacina L., Mateu R., Smetana K., Brábek J. (2016). Simultaneous blocking of IL-6 and IL-8 is sufficient to fully inhibit CAF-induced human melanoma cell invasiveness. Histochem. Cell Biol..

[191] Xu J., Lu Y., Qiu S., Chen Z.N., Fan Z. (2013). A novel role of EMMPRIN/CD147 in transformation of quiescent fibroblasts to cancer-associated fibroblasts by breast cancer cells. Cancer Lett..

[192] Bochet L., Lehuédé C., Dauvillier S., Wang Y.Y., Dirat B., Laurent V., Dray C., Guiet R., Maridonneau-Parini I., Le Gonidec S. (2013). Adipocyte-derived fibroblasts promote tumor progression and contribute to the desmoplastic reaction in breast cancer. Cancer Res..

[193] Kwan M.D., Sellmyer M.A., Quarto N., Ho A.M., Wandless T.J., Longaker M.T. (2011). Chemical control of FGF-2 release for promoting calvarial healing with adipose stem cells. J. Biol. Chem..

[194] Atluri K., Seabold D., Hong L., Elangovan S., Salem A.K. (2015). Nanoplex-Mediated Codelivery of Fibroblast Growth Factor and Bone Morphogenetic Protein Genes Promotes Osteogenesis in Human Adipocyte-Derived Mesenchymal Stem Cells. Mol. Pharm..

[195] Suga H., Shigeura T., Matsumoto D., Inoue K., Kato H., Aoi N., Murase S., Sato K., Gonda K., Koshima I. (2007). Rapid expansion of human adipose-derived stromal cells preserving multipotency. Cytotherapy.

[196] Zhang Y., Nowicka A., Solley T.N., Wei C., Parikh A., Court L., Burks J.K., Andreeff M., Woodward W.A., Dadbin A. (2015). Stromal Cells Derived from Visceral and Obese Adipose Tissue Promote Growth of Ovarian Cancers. PLoS ONE.

[197] Trotter T.N., Gibson J.T., Sherpa T.L., Gowda P.S., Peker D., Yang Y. (2016). Adipocyte-Lineage Cells Support Growth and Dissemination of Multiple Myeloma in Bone. Am. J. Pathol..

[198] Pathak S., Nemeth M.A., Multani A.S., Thalmann G.N., von Eschenbach A.C., Chung L.W. (1997). Can cancer cells transform normal host cells into malignant cells?. Br. J. Cancer.

[199] Hill R., Song Y., Cardiff R.D., Van Dyke T. (2005). Selective evolution of stromal mesenchyme with p53 loss in response to epithelial tumorigenesis. Cell.

[200] Sung S.Y., Hsieh C.L., Law A., Zhau H.E., Pathak S., Multani A.S., Lim S., Coleman I.M., Wu L.C., Figg W.D. (2008). Coevolution of prostate cancer and bone stroma in three-dimensional coculture: Implications for cancer growth and metastasis. Cancer Res..

[201] Westhoff C.C., Jank P., Jacke C.O., Albert U.S., Ebrahimsade S., Barth P.J., Moll R. (2020). Prognostic relevance of the loss of stromal CD34 positive fibroblasts in invasive lobular carcinoma of the breast. Virchows Arch..

[202] Rozenchan P.B., Pasini F.S., Roela R.A., Katayama M.L., Mundim F.G., Brentani H., Lyra E.C., Brentani M.M. (2015). Specific upregulation of RHOA and RAC1 in cancer-associated fibroblasts found at primary tumor and lymph node metastatic sites in breast cancer. Tumour Biol..

[203] Plava J., Cihova M., Burikova M., Bohac M., Adamkov M., Drahosova S., Rusnakova D., Pindak D., Karaba M., Simo J. (2020). Permanent Pro-Tumorigenic Shift in Adipose Tissue-Derived Mesenchymal Stromal Cells Induced by Breast Malignancy. Cells.

[204] Zhang Y., Daquinag A.C., Amaya-Manzanares F., Sirin O., Tseng C., Kolonin M.G. (2012). Stromal progenitor cells from endogenous adipose tissue contribute to pericytes and adipocytes that populate the tumor microenvironment. Cancer Res..

[205] Oudin M.J., Weaver V.M. (2016). Physical and Chemical Gradients in the Tumor Microenvironment Regulate Tumor Cell Invasion, Migration, and Metastasis. Cold Spring Harb. Symp. Quant. Biol..

[206] Shu S.L., Yang Y., Allen C.L., Maguire O., Minderman H., Sen A., Ciesielski M.J., Collins K.A., Bush P.J., Singh P. (2018). Metabolic reprogramming of stromal fibroblasts by melanoma exosome microRNA favours a pre-metastatic microenvironment. Sci. Rep..

[207] Liu D., Hornsby P.J. (2007). Senescent Human Fibroblasts Increase the Early Growth of Xenograft Tumors via Matrix Metalloproteinase Secretion. Cancer Res..

[208] Guan X., LaPak K.M., Hennessey R.C., Yu C.Y., Shakya R., Zhang J., Burd C.E. (2017). Stromal Senescence by Prolonged CDK4/6 Inhibition Potentiates Tumor Growth. Mol. Cancer Res..

[209] Krisnawan V.E., Stanley J.A., Schwarz J.K., DeNardo D.G. (2020). Tumor Microenvironment as a Regulator of Radiation Therapy: New Insights into Stromal-Mediated Radioresistance. Cancers.

[210] Ragunathan K., Upfold N.L.E., Oksenych V. (2020). Interaction between Fibroblasts and Immune Cells Following DNA Damage Induced by Ionizing Radiation. Int. J. Mol. Sci..

[211] Pazolli E., Alspach E., Milczarek A., Prior J., Piwnica-Worms D., Stewart S.A. (2012). Chromatin remodeling underlies the senescence-associated secretory phenotype of tumor stromal fibroblasts that supports cancer progression. Cancer Res..

[212] Yoshimoto S., Loo T.M., Atarashi K., Kanda H., Sato S., Oyadomari S., Iwakura Y., Oshima K., Morita H., Hattori M. (2013). Obesity-induced gut microbial metabolite promotes liver cancer through senescence secretome. Nature.

[213] Li H., Liu P., Xu S., Li Y., Dekker J.D., Li B., Fan Y., Zhang Z., Hong Y., Yang G. (2017). Foxp1 controls mesenchymal stem cell commitment and senescence during skeletal aging. J. Clin. Investig..

[214] Takasugi M., Yoshida Y., Ohtani N. (2022). Cellular senescence and the tumour microenvironment. Mol. Oncol..

[215] Zhang J., Li S., Zhao Y., Ma P., Cao Y., Liu C., Zhang X., Wang W., Chen L., Li Y. (2020). Cancer-associated fibroblasts promote the migration and invasion of gastric cancer cells via activating IL-17a/JAK2/STAT3 signaling. Ann. Transl. Med..

[216] Kankuri E., Babusikova O., Hlubinova K., Salmenperä P., Boccaccio C., Lubitz W., Harjula A., Bizik J. (2007). Fibroblast nemosis arrests growth and induces differentiation of human leukemia cells. Int. J. Cancer.

[217] Vaheri A., Enzerink A., Räsänen K., Salmenperä P. (2009). Nemosis, a novel way of fibroblast activation, in inflammation and cancer. Exp. Cell Res..

[218] Rasanen K., Virtanen I., Salmenpera P., Grenman R., Vaheri A. (2009). Differences in the nemosis response of normal and cancer-associated fibroblasts from patients with oral squamous cell carcinoma. PLoS ONE.

[219] De Wever O., Demetter P., Mareel M., Bracke M. (2008). Stromal myofibroblasts are drivers of invasive cancer growth. Int. J Cancer.

[220] Peltier A., Seban R.-D., Buvat I., Bidard F.-C., Mechta-Grigoriou F. (2022). Fibroblast heterogeneity in solid tumors: From single cell analysis to whole-body imaging. Semin. Cancer Biol..

[221] Li J., Chen K., Liu H., Cheng K., Yang M., Zhang J., Cheng J.D., Zhang Y., Cheng Z. (2012). Activatable near-infrared fluorescent probe for in vivo imaging of fibroblast activation protein-alpha. Bioconjug. Chem..

[222] Mona C.E., Benz M.R., Hikmat F., Grogan T.R., Lueckerath K., Razmaria A., Riahi R., Slavik R., Girgis M.D., Carlucci G. (2022). Correlation of (68)Ga-FAPi-46 PET Biodistribution with FAP Expression by Immunohistochemistry in Patients with Solid Cancers: Interim Analysis of a Prospective Translational Exploratory Study. J. Nucl. Med..

[223] Feldmann K., Maurer C., Peschke K., Teller S., Schuck K., Steiger K., Engleitner T., Öllinger R., Nomura A., Wirges N. (2021). Mesenchymal Plasticity Regulated by Prrx1 Drives Aggressive Pancreatic Cancer Biology. Gastroenterology.

[224] Cheng X.-B., Sato N., Kohi S., Koga A., Hirata K. (2018). 4-Methylumbelliferone inhibits enhanced hyaluronan synthesis and cell migration in pancreatic cancer cells in response to tumor-stromal interactions. Oncol. Lett..

[225] Wu H., Ma S., Xiang M., Tong S. (2019). HTRA1 promotes transdifferentiation of normal fibroblasts to cancer-associated fibroblasts through activation of the NF-κB/bFGF signaling pathway in gastric cancer. Biochem. Biophys. Res. Commun..

[226] Zheng L., Xu C., Guan Z., Su X., Xu Z., Cao J., Teng L. (2016). Galectin-1 mediates tgf-beta-induced transformation from normal fibroblasts into carcinoma-associated fibroblasts and promotes tumor progression in gastric cancer. Am. J. Transl. Res..

[227] Wen S., Niu Y., Yeh S., Chang C. (2015). BM-MSCs promote prostate cancer progression via the conversion of normal fibroblasts to cancer-associated fibroblasts. Int. J. Oncol..

[228] Zhao W., Ajani J.A., Sushovan G., Ochi N., Hwang R., Hafley M., Johnson R.L., Bresalier R.S., Logsdon C.D., Zhang Z. (2018). Galectin-3 Mediates Tumor Cell–Stroma Interactions by Activating Pancreatic Stellate Cells to Produce Cytokines via Integrin Signaling. Gastroenterology.

[229] Farhood B., Khodamoradi E., Hoseini-Ghahfarokhi M., Motevaseli E., Mirtavoos-Mahyari H., Eleojo Musa A., Najafi M. (2020). TGF-β in radiotherapy: Mechanisms of tumor resistance and normal tissues injury. Pharmacol. Res..

[230] Yoshida G.J. (2020). Regulation of heterogeneous cancer-associated fibroblasts: The molecular pathology of activated signaling pathways. J. Exp. Clin. Cancer Res..

[231] Liu B., Liu T., Liu Y., Feng X., Jiang X., Long J., Ye S., Chen D., Wang J., Yang Z. (2022). TSG-6 promotes Cancer Cell aggressiveness in a CD44-Dependent Manner and Reprograms Normal Fibroblasts to create a Pro-metastatic Microenvironment in Colorectal Cancer. Int. J. Biol. Sci..

[232] Legrand A.J., Poletto M., Pankova D., Clementi E., Moore J., Castro-Giner F., Ryan A.J., O’Neill E., Markkanen E., Dianov G.L. (2018). Persistent DNA strand breaks induce a CAF-like phenotype in normal fibroblasts. Oncotarget.

[233] Bar J., Feniger-Barish R., Lukashchuk N., Shaham H., Moskovits N., Goldfinger N., Simansky D., Perlman M., Papa M., Yosepovich A. (2009). Cancer cells suppress p53 in adjacent fibroblasts. Oncogene.

[234] Biasoli D., Sobrinho M.F., da Fonseca A.C.C., de Matos D.G., Romão L., de Moraes Maciel R., Rehen S.K., Moura-Neto V., Borges H.L., Lima F.R.S. (2014). Glioblastoma cells inhibit astrocytic p53-expression favoring cancer malignancy. Oncogenesis.

[235] Arandkar S., Furth N., Elisha Y., Nataraj N.B., van der Kuip H., Yarden Y., Aulitzky W., Ulitsky I., Geiger B., Oren M. (2018). Altered p53 functionality in cancer-associated fibroblasts contributes to their cancer-supporting features. Proc. Natl. Acad. Sci. USA.

[236] Addadi Y., Moskovits N., Granot D., Lozano G., Carmi Y., Apte R.N., Neeman M., Oren M. (2010). p53 status in stromal fibroblasts modulates tumor growth in an SDF1-dependent manner. Cancer Res..

[237] Farmaki E., Chatzistamou I., Bourlis P., Santoukou E., Trimis G., Papavassiliou A.G., Kiaris H. (2012). Selection of p53-Deficient Stromal Cells in the Tumor Microenvironment. Genes Cancer.

[238] Bientinesi E., Lulli M., Becatti M., Ristori S., Margheri F., Monti D. (2022). Doxorubicin-induced senescence in normal fibroblasts promotes in vitro tumour cell growth and invasiveness: The role of Quercetin in modulating these processes. Mech. Ageing Dev..

[239] Kim E., Kim W., Lee S., Chun J., Kang J., Park G., Han I., Yang H.J., Youn H., Youn B. (2017). TRAF4 promotes lung cancer aggressiveness by modulating tumor microenvironment in normal fibroblasts. Sci. Rep..

[240] Shen T., Li Y., Zhu S., Yu J., Zhang B., Chen X., Zhang Z., Ma Y., Niu Y., Shang Z. (2020). YAP1 plays a key role of the conversion of normal fibroblasts into cancer-associated fibroblasts that contribute to prostate cancer progression. J. Exp. Clin. Cancer Res..

[241] Nabet B.Y., Qiu Y., Shabason J.E., Wu T.J., Yoon T., Kim B.C., Benci J.L., DeMichele A.M., Tchou J., Marcotrigiano J. (2017). Exosome RNA Unshielding Couples Stromal Activation to Pattern Recognition Receptor Signaling in Cancer. Cell.

[242] Scioli M.G., Terriaca S., Fiorelli E., Storti G., Fabbri G., Cervelli V., Orlandi A. (2021). Extracellular Vesicles and Cancer Stem Cells in Tumor Progression: New Therapeutic Perspectives. Int. J. Mol. Sci..

[243] Guo H., Ha C., Dong H., Yang Z., Ma Y., Ding Y. (2019). Cancer-associated fibroblast-derived exosomal microRNA-98-5p promotes cisplatin resistance in ovarian cancer by targeting CDKN1A. Cancer Cell Int..

[244] Bao Q., Huang Q., Chen Y., Wang Q., Sang R., Wang L., Xie Y., Chen W. (2022). Tumor-Derived Extracellular Vesicles Regulate Cancer Progression in the Tumor Microenvironment. Front. Mol. Biosci..

[245] Jena B.C., Mandal M. (2021). The emerging roles of exosomes in anti-cancer drug resistance and tumor progression: An insight towards tumor-microenvironment interaction. Biochim. Biophys. Acta–Rev. Cancer.

[246] Wang X., Zhou Y., Ding K. (2021). Roles of exosomes in cancer chemotherapy resistance, progression, metastasis and immunity, and their clinical applications. Int. J. Oncol..

[247] Bach D.-H., Hong J.-Y., Park H.J., Lee S.K. (2017). The role of exosomes and miRNAs in drug-resistance of cancer cells. Int. J. Cancer.

[248] Boussadia Z., Gambardella A.R., Mattei F., Parolini I. (2021). Acidic and Hypoxic Microenvironment in Melanoma: Impact of Tumour Exosomes on Disease Progression. Cells.

[249] Zhu S., Li S., Yi M., Li N., Wu K. (2021). Roles of Microvesicles in Tumor Progression and Clinical Applications. Int. J. Nanomed..

[250] Wei X., Shi Y., Dai Z., Wang P., Meng X., Yin B. (2021). Underlying metastasis mechanism and clinical application of exosomal circular RNA in tumors. Int. J. Oncol..

[251] Yang Y., Li J., Geng Y. (2020). Exosomes derived from chronic lymphocytic leukaemia cells transfer miR-146a to induce the transition of mesenchymal stromal cells into cancer-associated fibroblasts. J. Biochem..

[252] Mitra A.K., Zillhardt M., Hua Y., Tiwari P., Murmann A.E., Peter M.E., Lengyel E. (2012). MicroRNAs reprogram normal fibroblasts into cancer-associated fibroblasts in ovarian cancer. Cancer Discov..

[253] Pang W., Su J., Wang Y., Feng H., Dai X., Yuan Y., Chen X., Yao W. (2015). Pancreatic cancer-secreted miR-155 implicates in the conversion from normal fibroblasts to cancer-associated fibroblasts. Cancer Sci..

[254] Cosentino G., Romero-Cordoba S., Plantamura I., Cataldo A., Iorio M.V. (2020). miR-9-Mediated Inhibition of EFEMP1 Contributes to the Acquisition of Pro-Tumoral Properties in Normal Fibroblasts. Cells.

[255] Tomita T., Kato M., Mishima T., Matsunaga Y., Sanjo H., Ito K.-I., Minagawa K., Matsui T., Oikawa H., Takahashi S. (2021). Extracellular mRNA transported to the nucleus exerts translation-independent function. Nat. Commun..

[256] Deng L., Wang Y., Peng Y., Wu Y., Ding Y., Jiang Y., Shen Z., Fu Q. (2015). Osteoblast-derived microvesicles: A novel mechanism for communication between osteoblasts and osteoclasts. Bone.

[257] Wen S.W., Sceneay J., Lima L.G., Wong C.S., Becker M., Krumeich S., Lobb R.J., Castillo V., Wong K.N., Ellis S. (2016). The biodistribution and immune suppressive effects of breast cancer-derived exosomes. Cancer Res..

[258] Zhang G., Zhang W., Li B., Stringer-Reasor E., Chu C., Sun L., Bae S., Chen D., Wei S., Jiao K. (2017). MicroRNA-200c and microRNA-141 are regulated by a foxp3-kat2b axis and associated with tumor metastasis in breast cancer. Breast Cancer Res..

[259] Matei I., Kim H.S., Lyden D. (2017). Unshielding Exosomal RNA Unleashes Tumor Growth and Metastasis. Cell.

[260] Suetsugu A., Matsumoto T., Hasegawa K., Nakamura M., Kunisada T., Shimizu M., Saji S., Moriwaki H., Bouvet M., Hoffman R.M. (2016). Color-coded live imaging of heterokaryon formation and nuclear fusion of hybridizing cancer cells. Anticancer Res..

[261] Chen Y., Zeng C., Zhan Y., Wang H., Jiang X., Li W. (2017). Aberrant low expression of p85alpha in stromal fibroblasts promotes breast cancer cell metastasis through exosome-mediated paracrine Wnt10b. Oncogene.

[262] Feng Q., Zhang C., Lum D., Druso J.E., Blank B., Wilson K.F., Welm A., Antonyak M.A., Cerione R.A. (2017). A class of extracellular vesicles from breast cancer cells activates VEGF receptors and tumour angiogenesis. Nat. Commun..

[263] Yan W., Wu X., Zhou W., Fong M.Y., Cao M., Liu J., Liu X., Chen C.H., Fadare O., Pizzo D.P. (2018). Cancer-cell-secreted exosomal mir-105 promotes tumour growth through the myc-dependent metabolic reprogramming of stromal cells. Nat. Cell Biol..

[264] Morrissey S.M., Yan J. (2020). Exosomal PD-L1: Roles in Tumor Progression and Immunotherapy. Trends Cancer.

[265] Unterleuthner D., Neuhold P., Schwarz K., Janker L., Neuditschko B., Nivarthi H., Crncec I., Kramer N., Unger C., Hengstschlager M. (2020). Cancer-associated fibroblast-derived wnt2 increases tumor angiogenesis in colon cancer. Angiogenesis.

[266] Dogra S., Hannafon B.N. (2021). Breast Cancer Microenvironment Cross Talk through Extracellular Vesicle RNAs. Am. J. Pathol..

[267] Yan Z., Sheng Z., Zheng Y., Feng R., Xiao Q., Shi L., Li H., Yin C., Luo H., Hao C. (2021). Cancer-associated fibroblast-derived exosomal miR-18b promotes breast cancer invasion and metastasis by regulating TCEAL7. Cell Death Dis..

[268] Chatterjee A., Jana S., Chatterjee S., Wastall L.M., Mandal G., Nargis N., Roy H., Hughes T.A., Bhattacharyya A. (2019). MicroRNA-222 reprogrammed cancer-associated fibroblasts enhance growth and metastasis of breast cancer. Br. J. Cancer.

[269] Baroni S., Romero-Cordoba S., Plantamura I., Dugo M., D’Ippolito E., Cataldo A., Cosentino G., Angeloni V., Rossini A., Daidone M.G. (2016). Exosome-mediated delivery of miR-9 induces cancer-associated fibroblast-like properties in human breast fibroblasts. Cell Death Dis..

[270] Huang Q., Hsueh C.-Y., Shen Y.-J., Guo Y., Huang J.-M., Zhang Y.-F., Li J.-Y., Gong H.-L., Zhou L. (2021). Small extracellular vesicle-packaged TGFβ1 promotes the reprogramming of normal fibroblasts into cancer-associated fibroblasts by regulating fibronectin in head and neck squamous cell carcinoma. Cancer Lett..

[271] Han Y., Qian X., Xu T., Shi Y. (2022). Carcinoma-associated fibroblasts release microRNA-331-3p containing extracellular vesicles to exacerbate the development of pancreatic cancer via the SCARA5-FAK axis. Cancer Biol. Ther..

[272] Yang R., Wang D., Han S., Gu Y., Li Z., Deng L., Yin A., Gao Y., Li X., Yu Y. (2022). MiR-206 suppresses the deterioration of intrahepatic cholangiocarcinoma and promotes sensitivity to chemotherapy by inhibiting interactions with stromal CAFs. Int. J. Biol. Sci..

[273] Jiang Y., Wang K., Lu X., Wang Y., Chen J. (2021). Cancer-associated fibroblasts-derived exosomes promote lung cancer progression by OIP5-AS1/ miR-142-5p/ PD-L1 axis. Mol. Immunol..

[274] Qiao X., Zhao F. (2022). Long non-coding RNA Opa interacting protein 5-antisense RNA 1 binds to micorRNA-34a to upregulate oncogenic PD-L1 in non-small cell lung cancer. Bioengineered.

[275] Yang F., Yan Y., Yang Y., Hong X., Wang M., Yang Z., Liu B., Ye L. (2020). MiR-210 in exosomes derived from CAFs promotes non-small cell lung cancer migration and invasion through PTEN/PI3K/AKT pathway. Cell. Signal..

[276] Wang M., Qin Z., Wan J., Yan Y., Duan X., Yao X., Jiang Z., Li W., Qin Z. (2022). Tumor-derived exosomes drive pre-metastatic niche formation in lung via modulating CCL1+ fibroblast and CCR8+ Treg cell interactions. Cancer Immunol. Immunother..

[277] Tanaka K., Miyata H., Sugimura K., Fukuda S., Kanemura T., Yamashita K., Miyazaki Y., Takahashi T., Kurokawa Y., Yamasaki M. (2015). miR-27 is associated with chemoresistance in esophageal cancer through transformation of normal fibroblasts to cancer-associated fibroblasts. Carcinogenesis.

[278] Yang T.-S., Yang X.-H., Chen X., Wang X.-D., Hua J., Zhou D.-L., Zhou B., Song Z.-S. (2014). MicroRNA-106b in cancer-associated fibroblasts from gastric cancer promotes cell migration and invasion by targeting PTEN. FEBS Lett..

[279] Zhou M., Wang S., Liu D., Zhou J. (2021). LINC01915 Facilitates the Conversion of Normal Fibroblasts into Cancer-Associated Fibroblasts Induced by Colorectal Cancer-Derived Extracellular Vesicles through the miR-92a-3p/KLF4/CH25H Axis. ACS Biomater. Sci. Eng..

[280] Dai G., Yao X., Zhang Y., Gu J., Geng Y., Xue F., Zhang J. (2018). Colorectal cancer cell–derived exosomes containing miR-10b regulate fibroblast cells via the PI3K/Akt pathway. Bull. Cancer.

[281] Du Q., Ye X., Lu S.-R., Li H., Liu H.-Y., Zhai Q., Yu B. (2021). Exosomal miR-30a and miR-222 derived from colon cancer mesenchymal stem cells promote the tumorigenicity of colon cancer through targeting MIA3. J. Gastrointest. Oncol..

[282] Hurtado P., Martínez-Pena I., Piñeiro R. (2020). Dangerous Liaisons: Circulating Tumor Cells (CTCs) and Cancer-Associated Fibroblasts (CAFs). Cancers.

[283] Mukaida N., Zhang D., Sasaki S.-I. (2020). Emergence of Cancer-Associated Fibroblasts as an Indispensable Cellular Player in Bone Metastasis Process. Cancers.

[284] Peng Y., Li Z., Yang P., Newton I.P., Ren H., Zhang L., Wu H., Li Z. (2014). Direct contacts with colon cancer cells regulate the differentiation of bone marrow mesenchymal stem cells into tumor associated fibroblasts. Biochem. Biophys. Res. Commun..

[285] Zhang J., Liu J., Liu Y., Wu W., Li X., Wu Y., Chen H., Zhang K., Gu L. (2015). miR-101 represses lung cancer by inhibiting interaction of fibroblasts and cancer cells by down-regulating CXCL12. Biomed. Pharmacother..

[286] Li B.L., Lu W., Qu J.J., Ye L., Du G.Q., Wan X.P. (2019). Loss of exosomal miR-148b from cancer-associated fibroblasts promotes endometrial cancer cell invasion and cancer metastasis. J. Cell. Physiol..

[287] Zhou L., Yang K., Wickett R.R., Kadekaro A.L., Zhang Y. (2016). Targeted deactivation of cancer-associated fibroblasts by β-catenin ablation suppresses melanoma growth. Tumor Biol..

[288] Hass R., von der Ohe J., Dittmar T. (2021). Hybrid Formation and Fusion of Cancer Cells In Vitro and In Vivo. Cancers.

[289] Platt J.L., Cascalho M. (2019). Cell Fusion in Malignancy: A Cause or Consequence? A Provocateur or Cure?. Cells.

[290] Zhou X., Platt J.L. (2011). Molecular and Cellular Mechanisms of Mammalian Cell Fusion. Advances in Experimental Medicine and Biology.

[291] Melzer C., von der Ohe J., Hass R. (2019). Involvement of Actin Cytoskeletal Components in Breast Cancer Cell Fusion with Human Mesenchymal Stroma/Stem-Like Cells. Int. J. Mol. Sci..

[292] Hass R., von der Ohe J., Ungefroren H. (2020). Impact of the Tumor Microenvironment on Tumor Heterogeneity and Consequences for Cancer Cell Plasticity and Stemness. Cancers.

[293] Bhatia B., Multani A.S., Patrawala L., Chen X., Calhoun-Davis T., Zhou J., Schroeder L., Schneider-Broussard R., Shen J., Pathak S. (2007). Evidence that senescent human prostate epithelial cells enhance tumorigenicity: Cell fusion as a potential mechanism and inhibition by p16INK4a and hTERT. Int. J. Cancer.

[294] Mondal Roy S., Sarkar M. (2011). Membrane fusion induced by small molecules and ions. J. Lipids.

[295] Freeman B.T., Jung J.P., Ogle B.M. (2016). Single-cell RNA-seq reveals activation of unique gene groups as a consequence of stem cell-parenchymal cell fusion. Sci. Rep..

[296] Navab R., Strumpf D., To C., Pasko E., Kim K.S., Park C.J., Hai J., Liu J., Jonkman J., Barczyk M. (2016). Integrin α11β1 regulates cancer stromal stiffness and promotes tumorigenicity and metastasis in non-small cell lung cancer. Oncogene.

[297] Melzer C., von der Ohe J., Hass R. (2018). In Vitro Fusion of Normal and Neoplastic Breast Epithelial Cells with Human Mesenchymal Stroma/Stem Cells Partially Involves Tumor Necrosis Factor Receptor Signaling. Stem Cells.

[298] Ohlund D., Elyada E., Tuveson D. (2014). Fibroblast heterogeneity in the cancer wound. J. Exp. Med..

[299] Weiler J., Dittmar T. (2019). Cell Fusion in Human Cancer: The Dark Matter Hypothesis. Cells.

[300] Noubissi F.K., Harkness T., Alexander C.M., Ogle B.M. (2015). Apoptosis-induced cancer cell fusion: A mechanism of breast cancer metastasis. FASEB J..

[301] Melzer C., von der Ohe J., Hass R. (2019). In Vivo Cell Fusion between Mesenchymal Stroma/Stem-Like Cells and Breast Cancer Cells. Cancers.

[302] Wang R., Lewis M.S., Lyu J., Zhau H.E., Pandol S.J., Chung L.W.K. (2020). Cancer-stromal cell fusion as revealed by fluorescence protein tracking. Prostate.

[303] Delespaul L., Gélabert C., Lesluyes T., Le Guellec S., Pérot G., Leroy L., Baud J., Merle C., Lartigue L., Chibon F. (2020). Cell-cell fusion of mesenchymal cells with distinct differentiations triggers genomic and transcriptomic remodelling toward tumour aggressiveness. Sci. Rep..

[304] Zhang L.N., Zhang D.D., Yang L., Gu Y.X., Zuo Q.P., Wang H.Y., Xu J., Liu D.X. (2021). Roles of cell fusion between mesenchymal stromal/stem cells and malignant cells in tumor growth and metastasis. FEBS J..

[305] Ippolito L., Morandi A., Taddei M.L., Parri M., Comito G., Iscaro A., Raspollini M.R., Magherini F., Rapizzi E., Masquelier J. (2019). Cancer-associated fibroblasts promote prostate cancer malignancy via metabolic rewiring and mitochondrial transfer. Oncogene.

[306] Pasquier J., Guerrouahen B.S., Al Thawadi H., Ghiabi P., Maleki M., Abu-Kaoud N., Jacob A., Mirshahi M., Galas L., Rafii S. (2013). Preferential transfer of mitochondria from endothelial to cancer cells through tunneling nanotubes modulates chemoresistance. J. Transl. Med..

[307] Moschoi R., Imbert V., Nebout M., Chiche J., Mary D., Prebet T., Saland E., Castellano R., Pouyet L., Collette Y. (2016). Protective mitochondrial transfer from bone marrow stromal cells to acute myeloid leukemic cells during chemotherapy. Blood.

[308] Lin H.-Y., Liou C.-W., Chen S.-D., Hsu T.-Y., Chuang J.-H., Wang P.-W., Huang S.-T., Tiao M.-M., Chen J.-B., Lin T.-K. (2015). Mitochondrial transfer from Wharton’s jelly-derived mesenchymal stem cells to mitochondria-defective cells recaptures impaired mitochondrial function. Mitochondrion.

[309] Gomzikova M.O., James V., Rizvanov A.A. (2021). Mitochondria Donation by Mesenchymal Stem Cells: Current Understanding and Mitochondria Transplantation Strategies. Front. Cell Dev. Biol..

[310] Merle C., Lagarde P., Lartigue L., Chibon F. (2021). Acquisition of cancer stem cell capacities after spontaneous cell fusion. BMC Cancer.

[311] Yang Y., Otte A., Hass R. (2015). Human mesenchymal stroma/stem cells exchange membrane proteins and alter functionality during interaction with different tumor cell lines. Stem Cells Dev..

[312] Chen Y.-C., Gonzalez M.E., Burman B., Zhao X., Anwar T., Tran M., Medhora N., Hiziroglu A.B., Lee W., Cheng Y.-H. (2019). Mesenchymal Stem/Stromal Cell Engulfment Reveals Metastatic Advantage in Breast Cancer. Cell Rep..

[313] Melzer C., von der Ohe J., Hass R. (2018). Enhanced metastatic capacity of breast cancer cells after interaction and hybrid formation with mesenchymal stroma/stem cells (MSC). Cell Commun. Signal..

[314] Melzer C., von der Ohe J., Hass R. (2018). MSC stimulate ovarian tumor growth during intercellular communication but reduce tumorigenicity after fusion with ovarian cancer cells. Cell Commun. Signal..

[315] Li M., Li X., Zhao L., Zhou J., Cheng Y., Xu B., Wang J., Wei L. (2019). Spontaneous formation of tumorigenic hybrids between human omental adipose-derived stromal cells and endometrial cancer cells increased motility and heterogeneity of cancer cells. Cell Cycle.

[316] Wei H.-J., Nickoloff J.A., Chen W.-H., Liu H.-Y., Lo W.-C., Chang Y.-T., Yang P.-C., Wu C.-W., Williams D.F., Gelovani J.G. (2014). FOXF1 mediates mesenchymal stem cell fusion-induced reprogramming of lung cancer cells. Oncotarget.

[317] Xu M.-H., Gao X., Luo D., Zhou X.-D., Xiong W., Liu G.-X. (2014). EMT and acquisition of stem cell-like properties are involved in spontaneous formation of tumorigenic hybrids between lung cancer and bone marrow-derived mesenchymal stem cells. PLoS ONE.

[318] Zhang L.N., Kong C.F., Zhao D., Cong X.L., Wang S.S., Ma L., Huang Y.H. (2019). Fusion with mesenchymal stem cells differentially affects tumorigenic and metastatic abilities of lung cancer cells. J. Cell. Physiol..

[319] Li H., Feng Z., Tsang T.C., Tang T., Jia X., He X., Pennington M.E., Badowski M.S., Liu A.K.M., Chen D. (2014). Fusion of HepG2 cells with mesenchymal stem cells increases cancer-associated and malignant properties: An in vivo metastasis model. Oncol. Rep..

[320] Xue J., Zhu Y., Sun Z., Ji R., Zhang X., Xu W., Yuan X., Zhang B., Yan Y., Yin L. (2015). Tumorigenic hybrids between mesenchymal stem cells and gastric cancer cells enhanced cancer proliferation, migration and stemness. BMC Cancer.

[321] Wang Y., Fan H., Zhou B., Ju Z., Yu L., Guo L., Han J., Lu S. (2012). Fusion of human umbilical cord mesenchymal stem cells with esophageal carcinoma cells inhibits the tumorigenicity of esophageal carcinoma cells. Int. J. Oncol..

[322] Liu C., Billet S., Choudhury D., Cheng R., Haldar S., Fernandez A., Biondi S., Liu Z., Zhou H., Bhowmick N.A. (2021). Bone marrow mesenchymal stem cells interact with head and neck squamous cell carcinoma cells to promote cancer progression and drug resistance. Neoplasia.

[323] Wang H., Tan L., Dong X., Liu L., Jiang Q., Li H., Shi J., Yang X., Dai X., Qian Z. (2020). MiR-146b-5p suppresses the malignancy of GSC/MSC fusion cells by targeting SMARCA5. Aging.

[324] Sun C., Zhao D., Dai X., Chen J., Rong X., Wang H., Wang A., Li M., Dong J.U.N., Huang Q. (2015). Fusion of cancer stem cells and mesenchymal stem cells contributes to glioma neovascularization. Oncol. Rep..

[325] Sun C., Dai X., Zhao D., Wang H., Rong X., Huang Q., Lan Q. (2019). Mesenchymal stem cells promote glioma neovascularization in vivo by fusing with cancer stem cells. BMC Cancer.

[326] Lartigue L., Merle C., Lagarde P., Delespaul L., Lesluyes T., Le Guellec S., Pérot G., Leroy L., Coindre J.-M., Chibon F. (2020). Genome remodeling upon mesenchymal tumor cell fusion contributes to tumor progression and metastatic spread. Oncogene.

[327] Manjunath Y., Porciani D., Mitchem J.B., Suvilesh K.N., Avella D.M., Kimchi E.T., Staveley-O’Carroll K.F., Burke D.H., Li G., Kaifi J.T. (2020). Tumor-Cell-Macrophage Fusion Cells as Liquid Biomarkers and Tumor Enhancers in Cancer. Int. J. Mol. Sci..

[328] Reduzzi C., Vismara M., Gerratana L., Silvestri M., De Braud F., Raspagliesi F., Verzoni E., Di Cosimo S., Locati L.D., Cristofanilli M. (2020). The curious phenomenon of dual-positive circulating cells: Longtime overlooked tumor cells. Semin. Cancer Biol..

[329] Zhang L.-N., Huang Y.-H., Zhao L. (2019). Fusion of macrophages promotes breast cancer cell proliferation, migration and invasion through activating epithelial-mesenchymal transition and Wnt/β-catenin signaling pathway. Arch. Biochem. Biophys..

[330] Hass R., von der Ohe J., Ungefroren H. (2019). Potential Role of MSC/Cancer Cell Fusion and EMT for Breast Cancer Stem Cell Formation. Cancers.

[331] Friedman G., Levi-Galibov O., David E., Bornstein C., Giladi A., Dadiani M., Mayo A., Halperin C., Pevsner-Fischer M., Lavon H. (2020). Cancer-associated fibroblast compositions change with breast cancer progression linking the ratio of S100A4+ and PDPN+ CAFs to clinical outcome. Nat. Cancer.

[332] Huang Y., Zhou S., Huang Y., Zheng D., Mao Q., He J., Wang Y., Xue D., Lu X., Yang N. (2017). Isolation of Fibroblast-Activation Protein-Specific Cancer-Associated Fibroblasts. Biomed. Res. Int..

[333] Fuhr L., Abreu M., Carbone A., El-Athman R., Bianchi F., Laukkanen M.O., Mazzoccoli G., Relógio A. (2019). The Interplay between Colon Cancer Cells and Tumour-Associated Stromal Cells Impacts the Biological Clock and Enhances Malignant Phenotypes. Cancers.

[334] Agorku D.J., Langhammer A., Heider U., Wild S., Bosio A., Hardt O. (2019). CD49b, CD87, and CD95 Are Markers for Activated Cancer-Associated Fibroblasts Whereas CD39 Marks Quiescent Normal Fibroblasts in Murine Tumor Models. Front. Oncol..

[335] Casey T., Bond J., Tighe S., Hunter T., Lintault L., Patel O., Eneman J., Crocker A., White J., Tessitore J. (2008). Molecular signatures suggest a major role for stromal cells in development of invasive breast cancer. Breast Cancer Res. Treat..

[336] Sadlonova A., Bowe D.B., Novak Z., Mukherjee S., Duncan V.E., Page G.P., Frost A.R. (2009). Identification of molecular distinctions between normal breast-associated fibroblasts and breast cancer-associated fibroblasts. Cancer Microenviron..

[337] Zhao L., Sun Y., Hou Y., Peng Q., Wang L., Luo H., Tang X., Zeng Z., Liu M. (2012). MiRNA expression analysis of cancer-associated fibroblasts and normal fibroblasts in breast cancer. Int. J. Biochem. Cell Biol..

[338] Peng Q., Zhao L., Hou Y., Sun Y., Wang L., Luo H., Peng H., Liu M. (2013). Biological characteristics and genetic heterogeneity between carcinoma-associated fibroblasts and their paired normal fibroblasts in human breast cancer. PLoS ONE.

[339] Pal B., Chen Y., Vaillant F., Capaldo B.D., Joyce R., Song X., Bryant V.L., Penington J.S., Di Stefano L., Tubau Ribera N. (2021). A single-cell RNA expression atlas of normal, preneoplastic and tumorigenic states in the human breast. EMBO J..

[340] Lim B., Lin Y., Navin N. (2020). Advancing Cancer Research and Medicine with Single-Cell Genomics. Cancer Cell.

[341] Catteau X., Simon P., Buxant F., Noël J.-C. (2016). Expression of the glucocorticoid receptor in breast cancer-associated fibroblasts. Mol. Clin. Oncol..

[342] Orr B., Riddick A.C.P., Stewart G.D., Anderson R.A., Franco O.E., Hayward S.W., Thomson A.A. (2012). Identification of stromally expressed molecules in the prostate by tag-profiling of cancer-associated fibroblasts, normal fibroblasts and fetal prostate. Oncogene.

[343] Maris P., Blomme A., Palacios A.P., Costanza B., Bellahcène A., Bianchi E., Gofflot S., Drion P., Trombino G.E., Di Valentin E. (2015). Asporin Is a Fibroblast-Derived TGF-β1 Inhibitor and a Tumor Suppressor Associated with Good Prognosis in Breast Cancer. PLoS Med..

[344] Allinen M., Beroukhim R., Cai L., Brennan C., Lahti-Domenici J., Huang H., Porter D., Hu M., Chin L., Richardson A. (2004). Molecular characterization of the tumor microenvironment in breast cancer. Cancer Cell.

[345] Orimo A., Gupta P.B., Sgroi D.C., Arenzana-Seisdedos F., Delaunay T., Naeem R., Carey V.J., Richardson A.L., Weinberg R.A. (2005). Stromal Fibroblasts Present in Invasive Human Breast Carcinomas Promote Tumor Growth and Angiogenesis through Elevated SDF-1/CXCL12 Secretion. Cell.

[346] Franco O.E., Jiang M., Strand D.W., Peacock J., Fernandez S., Jackson R.S., Revelo M.P., Bhowmick N.A., Hayward S.W. (2011). Altered TGF-β signaling in a subpopulation of human stromal cells promotes prostatic carcinogenesis. Cancer Res..

[347] Kiskowski M.A., Jackson R.S., Banerjee J., Li X., Kang M., Iturregui J.M., Franco O.E., Hayward S.W., Bhowmick N.A. (2011). Role for stromal heterogeneity in prostate tumorigenesis. Cancer Res..

[348] Ge S., Mao Y., Yi Y., Xie D., Chen Z., Xiao Z. (2012). Comparative proteomic analysis of secreted proteins from nasopharyngeal carcinoma-associated stromal fibroblasts and normal fibroblasts. Exp. Ther. Med..

[349] Ferrer-Mayorga G., Niell N., Cantero R., González-Sancho J.M., Del Peso L., Muñoz A., Larriba M.J. (2019). Vitamin D and Wnt3A have additive and partially overlapping modulatory effects on gene expression and phenotype in human colon fibroblasts. Sci. Rep..

[350] Kaya S., Wiesmann N., Goldschmitt J., Krüger M., Al-Nawas B., Heider J. (2021). Differences in the expression of caveolin-1 isoforms in cancer-associated and normal fibroblasts of patients with oral squamous cell carcinoma. Clin. Oral Investig..

[351] Fujimoto T., Kogo H., Nomura R., Une T. (2000). Isoforms of caveolin-1 and caveolar structure. J. Cell Sci..

[352] Walter S.G., Scheidt S., Nißler R., Gaisendrees C., Zarghooni K., Schildberg F.A. (2021). In-Depth Characterization of Stromal Cells within the Tumor Microenvironment Yields Novel Therapeutic Targets. Cancers.

[353] Martinez-Outschoorn U.E., Balliet R.M., Rivadeneira D.B., Chiavarina B., Pavlides S., Wang C., Whitaker-Menezes D., Daumer K.M., Lin Z., Witkiewicz A.K. (2010). Oxidative stress in cancer associated fibroblasts drives tumor-stroma co-evolution: A new paradigm for understanding tumor metabolism, the field effect and genomic instability in cancer cells. Cell Cycle.

[354] Lisanti M.P., Martinez-Outschoorn U.E., Chiavarina B., Pavlides S., Whitaker-Menezes D., Tsirigos A., Witkiewicz A., Lin Z., Balliet R., Howell A. (2010). Understanding the “lethal” drivers of tumor-stroma co-evolution: Emerging role(s) for hypoxia, oxidative stress and autophagy/mitophagy in the tumor micro-environment. Cancer Biol. Ther..

[355] Yan F., Zhang X., Tan R., Li M., Xiao Z., Wang H., Zhang Z., Ma Z., Liu Z. (2021). Autophagic flux in cancer cells at the invasive front in the tumor-stroma border. Aging.

[356] Ou J., Miao H., Ma Y., Guo F., Deng J., Wei X., Zhou J., Xie G., Shi H., Xue B. (2014). Loss of abhd5 promotes colorectal tumor development and progression by inducing aerobic glycolysis and epithelial-mesenchymal transition. Cell Rep..

[357] Domogauer J.D., de Toledo S.M., Howell R.W., Azzam E.I. (2021). Acquired radioresistance in cancer associated fibroblasts is concomitant with enhanced antioxidant potential and DNA repair capacity. Cell Commun. Signal..

[358] Bottoni G., Katarkar A., Tassone B., Ghosh S., Clocchiatti A., Goruppi S., Bordignon P., Jafari P., Tordini F., Lunardi T. (2019). CSL controls telomere maintenance and genome stability in human dermal fibroblasts. Nat. Commun..

[359] Casbas-Hernandez P., Fleming J.M., Troester M.A. (2011). Gene expression analysis of in vitro cocultures to study interactions between breast epithelium and stroma. J. Biomed. Biotechnol..

[360] Tchou J., Kossenkov A.V., Chang L., Satija C., Herlyn M., Showe L.C., Puré E. (2012). Human breast cancer associated fibroblasts exhibit subtype specific gene expression profiles. BMC Med. Genom..

[361] Katayama M.L.H., Vieira R.A.D.C., Andrade V.P., Roela R.A., Lima L.G.C.A., Kerr L.M., Campos A.P.D., Pereira C.A.D.B., Serio P.A.D.M.P., Encinas G. (2019). Stromal Cell Signature Associated with Response to Neoadjuvant Chemotherapy in Locally Advanced Breast Cancer. Cells.

[362] Desmedt C., Majjaj S., Kheddoumi N., Singhal S.K., Haibe-Kains B., El Ouriaghli F., Chaboteaux C., Michiels S., Lallemand F., Journe F. (2012). Characterization and clinical evaluation of CD10+ stroma cells in the breast cancer microenvironment. Clin. Cancer Res..

[363] Bartoschek M., Oskolkov N., Bocci M., Lövrot J., Larsson C., Sommarin M., Madsen C.D., Lindgren D., Pekar G., Karlsson G. (2018). Spatially and functionally distinct subclasses of breast cancer-associated fibroblasts revealed by single cell RNA sequencing. Nat. Commun..

[364] Infante J.R., Matsubayashi H., Sato N., Tonascia J., Klein A.P., Riall T.A., Yeo C., Iacobuzio-Donahue C., Goggins M. (2007). Peritumoral Fibroblast SPARC Expression and Patient Outcome with Resectable Pancreatic Adenocarcinoma. J. Clin. Oncol..

[365] Chlenski A., Guerrero L.J., Yang Q., Tian Y., Peddinti R., Salwen H.R., Cohn S.L. (2007). SPARC enhances tumor stroma formation and prevents fibroblast activation. Oncogene.

[366] Vered M., Shnaiderman-Shapiro A., Zlotogorski-Hurvitz A., Salo T., Yahalom R. (2019). Cancer-associated fibroblasts in the tumor microenvironment of tongue carcinoma is a heterogeneous cell population. Acta Histochem..

[367] Zhang Q., Wang Y., Xia C., Ding L., Pu Y., Hu X., Cai H., Hu Q. (2021). Integrated analysis of single-cell RNA-seq and bulk RNA-seq reveals distinct cancer-associated fibroblasts in head and neck squamous cell carcinoma. Ann. Transl. Med..

[368] Hosein A.N., Huang H., Wang Z., Parmar K., Du W., Huang J., Maitra A., Olson E., Verma U., Brekken R.A. (2019). Cellular heterogeneity during mouse pancreatic ductal adenocarcinoma progression at single-cell resolution. JCI Insight.

[369] Biffi G., Oni T.E., Spielman B., Hao Y., Elyada E., Park Y., Preall J., Tuveson D.A. (2019). IL1-Induced JAK/STAT Signaling Is Antagonized by TGFβ to Shape CAF Heterogeneity in Pancreatic Ductal Adenocarcinoma. Cancer Discov..

[370] Elyada E., Bolisetty M., Laise P., Flynn W.F., Courtois E.T., Burkhart R.A., Teinor J.A., Belleau P., Biffi G., Lucito M.S. (2019). Cross-Species Single-Cell Analysis of Pancreatic Ductal Adenocarcinoma Reveals Antigen-Presenting Cancer-Associated Fibroblasts. Cancer Discov..

[371] Wang L., Zhang F., Cui J.-Y., Chen L., Chen Y.-T., Liu B.-W. (2018). CAFs enhance paclitaxel resistance by inducing EMT through the IL-6/JAK2/STAT3 pathway. Oncol. Rep..

[372] Grunwald B.T., Devisme A., Andrieux G., Vyas F., Aliar K., McCloskey C.W., Macklin A., Jang G.H., Denroche R., Romero J.M. (2021). Spatially confined sub-tumor microenvironments in pancreatic cancer. Cell.

[373] Hutton C., Heider F., Blanco-Gomez A., Banyard A., Kononov A., Zhang X., Karim S., Paulus-Hock V., Watt D., Steele N. (2021). Single-cell analysis defines a pancreatic fibroblast lineage that supports anti-tumor immunity. Cancer Cell.

[374] Hu B., Wu C., Mao H., Gu H., Dong H., Yan J., Qi Z., Yuan L., Dong Q., Long J. (2022). Subpopulations of cancer-associated fibroblasts link the prognosis and metabolic features of pancreatic ductal adenocarcinoma. Ann. Transl. Med..

[375] Wheeler S.B., Reeder-Hayes K.E., Carey L.A. (2013). Disparities in breast cancer treatment and outcomes: Biological, social, and health system determinants and opportunities for research. Oncologist.

[376] Warner E.T., Tamimi R.M., Hughes M.E., Ottesen R.A., Wong Y.-N., Edge S.B., Theriault R.L., Blayney D.W., Niland J.C., Winer E.P. (2015). Racial and Ethnic Differences in Breast Cancer Survival: Mediating Effect of Tumor Characteristics and Sociodemographic and Treatment Factors. J. Clin. Oncol..

[377] Wieder R., Shafiq B., Adam N. (2020). Greater Survival Improvement in African American vs. Caucasian Women with Hormone Negative Breast Cancer. J. Cancer.

[378] Martin D.N., Boersma B.J., Yi M., Reimers M., Howe T.M., Yfantis H.G., Tsai Y.C., Williams E.H., Lee D.H., Stephens R.M. (2009). Differences in the tumor microenvironment between African-American and European-American breast cancer patients. PLoS ONE.

[379] Field L.A., Love B., Deyarmin B., Hooke J.A., Shriver C.D., Ellsworth R.E. (2011). Identification of differentially expressed genes in breast tumors from African American compared with Caucasian women. Cancer.

[380] D’Arcy M., Fleming J., Robinson W.R., Kirk E.L., Perou C.M., Troester M.A. (2015). Race-associated biological differences among Luminal A breast tumors. Breast Cancer Res. Treat..

[381] Daly B., Olopade O.I. (2015). A perfect storm: How tumor biology, genomics, and health care delivery patterns collide to create a racial survival disparity in breast cancer and proposed interventions for change. CA A Cancer J. Clin..

[382] Wieder R., Shafiq B., Adam N. (2016). African American Race is an Independent Risk Factor in Survival from Initially Diagnosed Localized Breast Cancer. J. Cancer.

[383] Huo D., Hu H., Rhie S.K., Gamazon E.R., Cherniack A.D., Liu J., Yoshimatsu T.F., Pitt J.J., Hoadley K.A., Troester M. (2017). Comparison of Breast Cancer Molecular Features and Survival by African and European Ancestry in The Cancer Genome Atlas. JAMA Oncol..

[384] Parada H., Sun X., Fleming J.M., Williams-DeVane C.R., Kirk E.L., Olsson L.T., Perou C.M., Olshan A.F., Troester M.A. (2017). Race-associated biological differences among luminal A and basal-like breast cancers in the Carolina Breast Cancer Study. Breast Cancer Res..

[385] Holowatyj A.N., Cote M.L., Ruterbusch J.J., Ghanem K., Schwartz A.G., Vigneau F.D., Gorski D.H., Purrington K.S. (2018). Racial Differences in 21-Gene Recurrence Scores Among Patients with Hormone Receptor-Positive, Node-Negative Breast Cancer. J. Clin. Oncol..

[386] Barrow M.A., Martin M.E., Coffey A., Andrews P.L., Jones G.S., Reaves D.K., Parker J.S., Troester M.A., Fleming J.M. (2019). A functional role for the cancer disparity-linked genes, CRYβB2 and CRYβB2P1, in the promotion of breast cancer. Breast Cancer Res..

[387] Purrington K.S., Gorski D., Simon M.S., Hastert T.A., Kim S., Rosati R., Schwartz A.G., Ratnam M. (2020). Racial differences in estrogen receptor staining levels and implications for treatment and survival among estrogen receptor positive, HER2-negative invasive breast cancers. Breast Cancer Res. Treat..

[388] Kim G., Pastoriza J.M., Condeelis J.S., Sparano J.A., Filippou P.S., Karagiannis G.S., Oktay M.H. (2020). The Contribution of Race to Breast Tumor Microenvironment Composition and Disease Progression. Front. Oncol..

[389] Fleming J.M., Miller T.C., Quinones M., Xiao Z., Xu X., Meyer M.J., Ginsburg E., Veenstra T.D., Vonderhaar B.K. (2010). The normal breast microenvironment of premenopausal women differentially influences the behavior of breast cancer cells in vitro and in vivo. BMC Med..

[390] Fantasia J., Lin C.B., Wiwi C., Kaur S., Hu Y.P., Zhang J., Southall M.D. (2013). Differential levels of elastin fibers and tgf-beta signaling in the skin of caucasians and african americans. J. Dermatol. Sci..

[391] Baker Frost D., da Silveira W., Hazard E.S., Atanelishvili I., Wilson R.C., Flume J., Day K.L., Oates J.C., Bogatkevich G.S., Feghali-Bostwick C. (2021). Differential DNA Methylation Landscape in Skin Fibroblasts from African Americans with Systemic Sclerosis. Genes.

[392] Mackey L.C., Annab L.A., Yang J., Rao B., Kissling G.E., Schurman S.H., Dixon D., Archer T.K. (2018). Epigenetic Enzymes, Age, and Ancestry Regulate the Efficiency of Human iPSC Reprogramming. Stem Cells.

[393] Catherino W.H., Eltoukhi H.M., Al-Hendy A. (2013). Racial and ethnic differences in the pathogenesis and clinical manifestations of uterine leiomyoma. Semin. Reprod. Med..

[394] Velez Edwards D.R., Tsosie K.S., Williams S.M., Edwards T.L., Russell S.B. (2014). Admixture mapping identifies a locus at 15q21.2-22.3 associated with keloid formation in African Americans. Hum. Genet..

[395] Jumper N., Paus R., Bayat A. (2015). Functional histopathology of keloid disease. Histol. Histopathol..

[396] Mirsaeidi M., Machado R.F., Schraufnagel D., Sweiss N.J., Baughman R.P. (2015). Racial difference in sarcoidosis mortality in the United States. Chest.

[397] Bogatkevich G.S., Ludwicka-Bradley A., Highland K.B., Hant F., Nietert P.J., Singleton C.B., Feghali-Bostwick C.A., Silver R.M. (2007). Impairment of the antifibrotic effect of hepatocyte growth factor in lung fibroblasts from African Americans: Possible role in systemic sclerosis. Arthritis Rheum..

[398] Reese C., Perry B., Heywood J., Bonner M., Visconti R.P., Lee R., Hatfield C.M., Silver R.M., Hoffman S., Tourkina E. (2014). Caveolin-1 deficiency may predispose African Americans to systemic sclerosis-related interstitial lung disease. Arthritis Rheumatol..

[399] Russell S.B., Russell J.D., Trupin K.M., Gayden A.E., Opalenik S.R., Nanney L.B., Broquist A.H., Raju L., Williams S.M. (2010). Epigenetically altered wound healing in keloid fibroblasts. J. Investig. Dermatol..

[400] Wang H., Parry S., Macones G., Sammel M.D., Kuivaniemi H., Tromp G., Argyropoulos G., Halder I., Shriver M.D., Romero R. (2006). A functional SNP in the promoter of the SERPINH1 gene increases risk of preterm premature rupture of membranes in African Americans. Proc. Natl. Acad. Sci. USA.

[401] Abraham M., Collins C.A., Flewelling S., Camazine M., Cahill A., Cade W.T., Duncan J.G. (2018). Mitochondrial inefficiency in infants born to overweight African-American mothers. Int. J. Obes..

[402] Yan Y., Narayan A., Cho S., Cheng Z., Liu J.O., Zhu H., Wang G., Wharram B., Lisok A., Brummet M. (2021). CRYβB2 enhances tumorigenesis through upregulation of nucleolin in triple negative breast cancer. Oncogene.

[403] Gillard M., Javier R., Ji Y., Zheng S.L., Xu J., Brendler C.B., Crawford S.E., Pierce B.L., Griend D.J.V., Franco O.E. (2018). Elevation of Stromal-Derived Mediators of Inflammation Promote Prostate Cancer Progression in African-American Men. Cancer Res..

[404] Mezheyeuski A., Bradic Lindh M., Guren T.K., Dragomir A., Pfeiffer P., Kure E.H., Ikdahl T., Skovlund E., Corvigno S., Strell C. (2016). Survival-associated heterogeneity of marker-defined perivascular cells in colorectal cancer. Oncotarget.

[405] Buchsbaum R.J., Oh S.Y. (2016). Breast Cancer-Associated Fibroblasts: Where We Are and Where We Need to Go. Cancers.

[406] Orimo A., Weinberg R.A. (2006). Stromal Fibroblasts in Cancer: A Novel Tumor-Promoting Cell Type. Cell Cycle.

[407] Luga V., Zhang L., Viloria-Petit A.M., Ogunjimi A.A., Inanlou M.R., Chiu E., Buchanan M., Hosein A.N., Basik M., Wrana J.L. (2012). Exosomes Mediate Stromal Mobilization of Autocrine Wnt-PCP Signaling in Breast Cancer Cell Migration. Cell.

[408] Dzobo K., Senthebane D.A., Rowe A., Thomford N.E., Mwapagha L.M., Al-Awwad N., Dandara C., Parker M.I. (2016). Cancer Stem Cell Hypothesis for Therapeutic Innovation in Clinical Oncology? Taking the Root Out, Not Chopping the Leaf. OMICS J. Integr. Biol..

[409] Bulle A., Lim K.-H. (2020). Beyond just a tight fortress: Contribution of stroma to epithelial-mesenchymal transition in pancreatic cancer. Signal. Transduct. Target Ther..

[410] Saunders N.A., Simpson F., Thompson E.W., Hill M.M., Endo-Munoz L., Leggatt G., Minchin R.F., Guminski A. (2012). Role of intratumoural heterogeneity in cancer drug resistance: Molecular and clinical perspectives. EMBO Mol. Med..

[411] Senthebane D.A., Rowe A., Thomford N.E., Shipanga H., Munro D., Mazeedi M.A.M.A., Almazyadi H.A.M., Kallmeyer K., Dandara C., Pepper M.S. (2017). The Role of Tumor Microenvironment in Chemoresistance: To Survive, Keep Your Enemies Closer. Int. J. Mol. Sci..

[412] Kai F., Drain A.P., Weaver V.M. (2019). The Extracellular Matrix Modulates the Metastatic Journey. Dev. Cell.

[413] Alexander J.I., Vendramini-Costa D.B., Francescone R., Luong T., Franco-Barraza J., Shah N., Gardiner J.C., Nicolas E., Raghavan K.S., Cukierman E. (2021). Palladin isoforms 3 and 4 regulate cancer-associated fibroblast pro-tumor functions in pancreatic ductal adenocarcinoma. Sci. Rep..

[414] Ansardamavandi A., Tafazzoli-Shadpour M. (2021). The functional cross talk between cancer cells and cancer associated fibroblasts from a cancer mechanics perspective. Biochim. Biophys. Acta–Mol. Cell Res..

[415] DuFort C.C., Paszek M.J., Weaver V.M. (2011). Balancing forces: Architectural control of mechanotransduction. Nat. Rev. Mol. Cell Biol..

[416] Kaur A., Ecker B.L., Douglass S.M., Kugel C.H., Webster M.R., Almeida F.V., Somasundaram R., Hayden J., Ban E., Ahmadzadeh H. (2019). Remodeling of the collagen matrix in aging skin promotes melanoma metastasis and affects immune cell motility. Cancer Discov..

[417] Kaur A., Webster M.R., Marchbank K., Behera R., Ndoye A., Kugel C.H., Dang V.M., Appleton J., O’Connell M.P., Cheng P. (2016). sFRP2 in the aged microenvironment drives melanoma metastasis and therapy resistance. Nature.

[418] Weniger M., Honselmann K.C., Liss A.S. (2018). The Extracellular Matrix and Pancreatic Cancer: A Complex Relationship. Cancers.

[419] Attieh Y., Clark A.G., Grass C., Richon S., Pocard M., Mariani P., Elkhatib N., Betz T., Gurchenkov B., Vignjevic D.M. (2017). Cancer-associated fibroblasts lead tumor invasion through integrin-β3-dependent fibronectin assembly. J. Cell Biol..

[420] Sperb N., Tsesmelis M., Wirth T. (2020). Crosstalk between Tumor and Stromal Cells in Pancreatic Ductal Adenocarcinoma. Int. J. Mol. Sci..

[421] Paszek M.J., Zahir N., Johnson K.R., Lakins J.N., Rozenberg G.I., Gefen A., Reinhart-King C.A., Margulies S.S., Dembo M., Boettiger D. (2005). Tensional homeostasis and the malignant phenotype. Cancer Cell.

[422] Plodinec M., Loparic M., Monnier C.A., Obermann E.C., Zanetti-Dallenbach R., Oertle P., Hyotyla J.T., Aebi U., Bentires-Alj M., Lim R.Y.H. (2012). The nanomechanical signature of breast cancer. Nat. Nanotechnol..

[423] Levental I., Georges P.C., Janmey P.A. (2007). Soft biological materials and their impact on cell function. Soft Matter.

[424] Ishihara S., Haga H. (2022). Matrix Stiffness Contributes to Cancer Progression by Regulating Transcription Factors. Cancers.

[425] Dzobo K., Senthebane D.A., Thomford N.E., Rowe A., Dandara C., Parker M.I. (2018). Not Everyone Fits the Mold: Intratumor and Intertumor Heterogeneity and Innovative Cancer Drug Design and Development. OMICS J. Integr. Biol..

[426] Korah R., Choi L., Barrios J., Wieder R. (2005). Expression of FGF-2 alters focal adhesion dynamics in migration-restricted MDA-MB-231 breast cancer cells. Breast Cancer Res. Treat..

[427] Cohen E., Tendler T., Lu H., Hansen C.K., Kertsman J., Barrios J., Wieder R. (2013). Collagen I provides a survival advantage to MD-1483 head and neck squamous cell carcinoma cells through phosphoinositol 3-kinase signaling. Anticancer Res..

[428] Sansing H.A., Sarkeshik A., Yates J.R., Patel V., Gutkind J.S., Yamada K.M., Berrier A.L. (2011). Integrin αβ1, αvβ, α6β effectors p130Cas, Src and talin regulate carcinoma invasion and chemoresistance. Biochem. Biophys. Res. Commun..

[429] Weigelin B., Bakker G.-J., Friedl P. (2012). Intravital third harmonic generation microscopy of collective melanoma cell invasion: Principles of interface guidance and microvesicle dynamics. Intravital.

[430] Shah P., Hobson C.M., Cheng S., Colville M.J., Paszek M.J., Superfine R., Lammerding J. (2021). Nuclear Deformation Causes DNA Damage by Increasing Replication Stress. Curr. Biol..

[431] Dai L., Li M., Zhang W.-L., Tang Y.-J., Tang Y.-L., Liang X.-H. (2021). Fibroblasts in cancer dormancy: Foe or friend?. Cancer Cell Int..

[432] Kuzet S.-E., Gaggioli C. (2016). Fibroblast activation in cancer: When seed fertilizes soil. Cell Tissue Res..

[433] Dawoud M.M., Jones D.T., Chelala C., Abdou A.G., Dreger S.A., Asaad N., Abd El-Wahed M., Jones L. (2022). Expression Profile of Myoepithelial Cells in DCIS: Do They Change from Protective Angels to Wicked Witches?. Appl. Immunohistochem. Mol. Morphol..

[434] Cirri P., Chiarugi P. (2012). Cancer-associated-fibroblasts and tumour cells: A diabolic liaison driving cancer progression. Cancer Metastasis Rev..

[435] Cirri P., Chiarugi P. (2011). Cancer associated fibroblasts: The dark side of the coin. Am. J. Cancer Res..

[436] Sharma V., Letson J., Furuta S. (2022). Fibrous stroma: Driver and passenger in cancer development. Sci. Signal..

[437] Chandler C., Liu T., Buckanovich R., Coffman L.G. (2019). The double edge sword of fibrosis in cancer. Transl. Res..

[438] Meng Q., Luo X., Chen J., Wang D., Chen E., Zhang W., Zhang G., Zhou W., Xu J., Song Z. (2020). Unmasking carcinoma-associated fibroblasts: Key transformation player within the tumor microenvironment. Biochim. Biophys. Acta–Rev. Cancer.

[439] Tanaka R., Kimura K., Eguchi S., Ohira G., Tanaka S., Amano R., Tanaka H., Yashiro M., Ohira M., Kubo S. (2022). Interleukin-8 produced from cancer-associated fibroblasts suppresses proliferation of the OCUCh-LM1 cancer cell line. BMC Cancer.

[440] Liao D., Luo Y., Markowitz D., Xiang R., Reisfeld R.A. (2009). Cancer associated fibroblasts promote tumor growth and metastasis by modulating the tumor immune microenvironment in a 4T1 murine breast cancer model. PLoS ONE.

[441] Dumont N., Liu B., Defilippis R.A., Chang H., Rabban J.T., Karnezis A.N., Tjoe J.A., Marx J., Parvin B., Tlsty T.D. (2013). Breast fibroblasts modulate early dissemination, tumorigenesis, and metastasis through alteration of extracellular matrix characteristics. Neoplasia.

[442] Yu Y., Xiao C.H., Tan L.D., Wang Q.S., Li X.Q., Feng Y.M. (2014). Cancer-associated fibroblasts induce epithelial-mesenchymal transition of breast cancer cells through paracrine TGF-β signalling. Br. J. Cancer.

[443] Choi Y.P., Lee J.H., Gao M.-Q., Kim B.G., Kang S., Kim S.H., Cho N.H. (2014). Cancer-associated fibroblast promote transmigration through endothelial brain cells in three-dimensional *in vitro* models. Int. J. Cancer.

[444] Fiori M.E., Di Franco S., Villanova L., Bianca P., Stassi G., De Maria R. (2019). Cancer-associated fibroblasts as abettors of tumor progression at the crossroads of EMT and therapy resistance. Mol. Cancer.

[445] Li H., Liu W., Zhang X., Wang Y. (2021). Cancer-associated fibroblast-secreted collagen triple helix repeat containing-1 promotes breast cancer cell migration, invasiveness and epithelial-mesenchymal transition by activating the Wnt/beta-catenin pathway. Oncol. Lett..

[446] Kan S., Konishi E., Arita T., Ikemoto C., Takenaka H., Yanagisawa A., Katoh N., Asai J. (2014). Podoplanin expression in cancer-associated fibroblasts predicts aggressive behavior in melanoma. J. Cutan. Pathol..

[447] Mhaidly R., Mechta-Grigoriou F. (2021). Role of cancer-associated fibroblast subpopulations in immune infiltration, as a new means of treatment in cancer. Immunol. Rev..

[448] Zhou Q., Wu X., Wang X., Yu Z., Pan T., Li Z., Chang X., Jin Z., Li J., Zhu Z. (2020). The reciprocal interaction between tumor cells and activated fibroblasts mediated by tnf-alpha/il-33/st2l signaling promotes gastric cancer metastasis. Oncogene.

[449] Yang M., Li D., Jiang Z., Li C., Ji S., Sun J., Chang Y., Ruan S., Wang Z., Liang R. (2022). TGF-β-Induced FLRT3 Attenuation Is Essential for Cancer-Associated Fibroblast–Mediated Epithelial–Mesenchymal Transition in Colorectal Cancer. Mol. Cancer Res..

[450] Timperi E., Gueguen P., Molgora M., Magagna I., Kieffer Y., Lopez-Lastra S., Sirven P., Baudrin L.G., Baulande S., Nicolas A. (2022). Lipid-Associated Macrophages Are Induced by Cancer-Associated Fibroblasts and Mediate Immune Suppression in Breast Cancer. Cancer Res..

[451] Teng F., Tian W.-Y., Wang Y.-M., Zhang Y.-F., Guo F., Zhao J., Gao C., Xue F.-X. (2016). Cancer-associated fibroblasts promote the progression of endometrial cancer via the SDF-1/CXCR4 axis. J. Hematol. Oncol..

[452] Grimm S., Jennek S., Singh R., Enkelmann A., Junker K., Rippaus N., Berndt A., Friedrich K. (2015). Malignancy of bladder cancer cells is enhanced by tumor-associated fibroblasts through a multifaceted cytokine-chemokine loop. Exp. Cell Res..

[453] Sugimoto T., Takiguchi Y., Kurosu K., Kasahara Y., Tanabe N., Tatsumi K., Hiroshima K., Minamihisamatsu M., Miyamoto T., Kuriyama T. (2005). Growth factor-mediated interaction between tumor cells and stromal fibroblasts in an experimental model of human small-cell lung cancer. Oncol. Rep..

[454] Palethorpe H.M., Leach D.A., Need E.F., Drew P.A., Smith E. (2018). Myofibroblast androgen receptor expression determines cell survival in co-cultures of myofibroblasts and prostate cancer cells in vitro. Oncotarget.

[455] Ishii K., Matsuoka I., Sasaki T., Nishikawa K., Kanda H., Imai H., Hirokawa Y., Iguchi K., Arima K., Sugimura Y. (2019). Loss of Fibroblast-Dependent Androgen Receptor Activation in Prostate Cancer Cells is Involved in the Mechanism of Acquired Resistance to Castration. J. Clin. Med..

[456] Camps J.L., Chang S.M., Hsu T.C., Freeman M.R., Hong S.J., Zhau H.E., von Eschenbach A.C., Chung L.W. (1990). Fibroblast-mediated acceleration of human epithelial tumor growth in vivo. Proc. Natl. Acad. Sci. USA.

[457] Eiro N., González L., Martínez-Ordoñez A., Fernandez-Garcia B., González L.O., Cid S., Dominguez F., Perez-Fernandez R., Vizoso F.J. (2018). Cancer-associated fibroblasts affect breast cancer cell gene expression, invasion and angiogenesis. Cell. Oncol..

[458] Eiro N., Fernandez-Garcia B., Vazquez J., Del Casar J.M., Gonzalez L.O., Vizoso F.J. (2015). A phenotype from tumor stroma based on the expression of metalloproteases and their inhibitors, associated with prognosis in breast cancer. Oncoimmunology.

[459] Mwafy S.E., El-Guindy D.M. (2020). Pathologic assessment of tumor-associated macrophages and their histologic localization in invasive breast carcinoma. J. Egypt. Natl. Cancer Inst..

[460] Mesker W.E., Liefers G.-J., Junggeburt J.M.C., van Pelt G.W., Alberici P., Kuppen P.J.K., Miranda N.F., van Leeuwen K.A.M., Morreau H., Szuhai K. (2009). Presence of a High Amount of Stroma and Downregulation of SMAD4 Predict for Worse Survival for Stage I–II Colon Cancer Patients. Anal. Cell. Pathol..

[461] Ligorio M., Sil S., Malagon-Lopez J., Nieman L.T., Misale S., Di Pilato M., Ebright R.Y., Karabacak M.N., Kulkarni A.S., Liu A. (2019). Stromal Microenvironment Shapes the Intratumoral Architecture of Pancreatic Cancer. Cell.

[462] Lou E., Vogel R.I., Hoostal S., Klein M., Linden M.A., Teoh D., Geller M.A. (2019). Tumor-Stroma Proportion as a Predictive Biomarker of Resistance to Platinum-Based Chemotherapy in Patients with Ovarian Cancer. JAMA Oncol..

[463] Zhang X., Zhang Y., Sun Z., Ma H. (2019). Research on correlation between tumor-stroma ratio and prognosis in non-small cell lung cancer. Minerva Med..

[464] Vangangelt K.M.H., Green A.R., Heemskerk I.M.F., Cohen D., van Pelt G.W., Sobral-Leite M., Schmidt M.K., Putter H., Rakha E.A., Tollenaar R.A.E.M. (2020). The prognostic value of the tumor-stroma ratio is most discriminative in patients with grade III or triple-negative breast cancer. Int. J. Cancer.

[465] Knudsen E.S., Vail P., Balaji U., Ngo H., Botros I.W., Makarov V., Riaz N., Balachandran V., Leach S., Thompson D.M. (2017). Stratification of Pancreatic Ductal Adenocarcinoma: Combinatorial Genetic, Stromal, and Immunologic Markers. Clin. Cancer Res..

[466] Torphy R.J., Wang Z., True-Yasaki A., Volmar K.E., Rashid N., Yeh B., Anderson J.M., Johansen J.S., Hollingsworth M.A., Yeh J.J. (2018). Stromal Content Is Correlated With Tissue Site, Contrast Retention, and Survival in Pancreatic Adenocarcinoma. JCO Precis. Oncol..

[467] Rhim A.D., Oberstein P.E., Thomas D.H., Mirek E.T., Palermo C.F., Sastra S.A., Dekleva E.N., Saunders T., Becerra C.P., Tattersall I.W. (2014). Stromal elements act to restrain, rather than support, pancreatic ductal adenocarcinoma. Cancer Cell.

[468] McAndrews K.M., Vazquez-Arreguin K., Kwak C., Sugimoto H., Zheng X., Li B., Kirtley M.L., LeBleu V.S., Kalluri R. (2021). alphaSMA^+^ fibroblasts suppress Lgr5^+^ cancer stem cells and restrain colorectal cancer progression. Oncogene..

[469] Jena B.C., Das C.K., Bharadwaj D., Mandal M. (2020). Cancer associated fibroblast mediated chemoresistance: A paradigm shift in understanding the mechanism of tumor progression. Biochim. Biophys. Acta–Rev. Cancer.

[470] Damhofer H., Medema J.P., Veenstra V.L., Badea L., Popescu I., Roelink H., Bijlsma M.F. (2013). Assessment of the stromal contribution to Sonic Hedgehog-dependent pancreatic adenocarcinoma. Mol. Oncol..

[471] Katoh M. (2019). Genomic testing, tumor microenvironment and targeted therapy of Hedgehog-related human cancers. Clin. Sci..

[472] Cazet A.S., Hui M.N., Elsworth B.L., Wu S.Z., Roden D., Chan C.-L., Skhinas J.N., Collot R., Yang J., Harvey K. (2018). Targeting stromal remodeling and cancer stem cell plasticity overcomes chemoresistance in triple negative breast cancer. Nat. Commun..

[473] Bellei B., Migliano E., Picardo M. (2020). A Framework of Major Tumor-Promoting Signal Transduction Pathways Implicated in Melanoma-Fibroblast Dialogue. Cancers.

[474] Higashino N., Koma Y.-I., Hosono M., Takase N., Okamoto M., Kodaira H., Nishio M., Shigeoka M., Kakeji Y., Yokozaki H. (2019). Fibroblast activation protein-positive fibroblasts promote tumor progression through secretion of CCL2 and interleukin-6 in esophageal squamous cell carcinoma. Lab. Investig..

[475] Shimizu M., Koma Y.-I., Sakamoto H., Tsukamoto S., Kitamura Y., Urakami S., Tanigawa K., Kodama T., Higashino N., Nishio M. (2021). Metallothionein 2A Expression in Cancer-Associated Fibroblasts and Cancer Cells Promotes Esophageal Squamous Cell Carcinoma Progression. Cancers.

[476] Lee Y.T., Tan Y.J., Falasca M., Oon C.E. (2020). Cancer-Associated Fibroblasts: Epigenetic Regulation and Therapeutic Intervention in Breast Cancer. Cancers.

[477] Xu L., Deng Q., Pan Y., Peng M., Wang X., Song L., Xiao M.A.N., Wang Z. (2014). Cancer-associated fibroblasts enhance the migration ability of ovarian cancer cells by increasing EZH2 expression. Int. J. Mol. Med..

[478] Albrengues J., Bertero T., Grasset E., Bonan S., Maiel M., Bourget I., Philippe C., Herraiz Serrano C., Benamar S., Croce O. (2015). Epigenetic switch drives the conversion of fibroblasts into proinvasive cancer-associated fibroblasts. Nat. Commun..

[479] Froeling F.E.M., Feig C., Chelala C., Dobson R., Mein C.E., Tuveson D.A., Clevers H., Hart I.R., Kocher H.M. (2011). Retinoic Acid–Induced Pancreatic Stellate Cell Quiescence Reduces Paracrine Wnt–β-Catenin Signaling to Slow Tumor Progression. Gastroenterology.

[480] Sherman M.H., Yu R.T., Engle D.D., Ding N., Atkins A.R., Tiriac H., Collisson E.A., Connor F., Van Dyke T., Kozlov S. (2014). Vitamin D receptor-mediated stromal reprogramming suppresses pancreatitis and enhances pancreatic cancer therapy. Cell.

[481] Banerjee S., Modi S., McGinn O., Zhao X., Dudeja V., Ramakrishnan S., Saluja A.K. (2016). Impaired Synthesis of Stromal Components in Response to Minnelide Improves Vascular Function, Drug Delivery, and Survival in Pancreatic Cancer. Clin. Cancer Res..

[482] Wheeler S.E., Shi H., Lin F., Dasari S., Bednash J., Thorne S., Watkins S., Joshi R., Thomas S.M. (2014). Enhancement of head and neck squamous cell carcinoma proliferation, invasion, and metastasis by tumor-associated fibroblasts in preclinical models. Head Neck.

[483] Kang S.H., Oh S.Y., Lee H.-J., Kwon T.-G., Kim J.-W., Lee S.-T., Choi S.-Y., Hong S.-H. (2021). Cancer-Associated Fibroblast Subgroups Showing Differential Promoting Effect on HNSCC Progression. Cancers.

[484] Lakins M.A., Ghorani E., Munir H., Martins C.P., Shields J.D. (2018). Cancer-associated fibroblasts induce antigen-specific deletion of CD8 (+) T Cells to protect tumour cells. Nat. Commun..

[485] Harryvan T.J., Visser M., de Bruin L., Plug L., Griffioen L., Mulder A., van Veelen P.A., van der Heden van Noort G.J., Jongsma M.L., Meeuwsen M.H. (2022). Enhanced antigen cross-presentation in human colorectal cancer-associated fibroblasts through upregulation of the lysosomal protease cathepsin S. J. Immunother. Cancer.

[486] Gui Y., Aguilar-Mahecha A., Krzemien U., Hosein A., Buchanan M., Lafleur J., Pollak M., Ferrario C., Basik M. (2019). Metastatic breast carcinoma-associated fibroblasts have enhanced protumorigenic properties related to increased igf2 expression. Clin. Cancer Res..

[487] Matsumura Y., Ito Y., Mezawa Y., Sulidan K., Daigo Y., Hiraga T., Mogushi K., Wali N., Suzuki H., Itoh T. (2019). Stromal fibroblasts induce metastatic tumor cell clusters via epithelial-mesenchymal plasticity. Life Sci. Alliance.

[488] Mercatali L., La Manna F., Miserocchi G., Liverani C., De Vita A., Spadazzi C., Bongiovanni A., Recine F., Amadori D., Ghetti M. (2017). Tumor-Stroma Crosstalk in Bone Tissue: The Osteoclastogenic Potential of a Breast Cancer Cell Line in a Co-Culture System and the Role of EGFR Inhibition. Int. J. Mol. Sci..

[489] Itoh G., Takagane K., Fukushi Y., Kuriyama S., Umakoshi M., Goto A., Yanagihara K., Yashiro M., Tanaka M. (2022). Cancer-associated fibroblasts educate normal fibroblasts to facilitate cancer cell spreading and T-cell suppression. Mol. Oncol..

[490] Cho H., Seo Y., Loke K.M., Kim S.-W., Oh S.-M., Kim J.-H., Soh J., Kim H.S., Lee H., Kim J. (2018). Cancer-Stimulated CAFs Enhance Monocyte Differentiation and Protumoral TAM Activation via IL6 and GM-CSF Secretion. Clin. Cancer Res..

[491] Ene-Obong A., Clear A.J., Watt J., Wang J., Fatah R., Riches J.C., Marshall J.F., Chin-Aleong J., Chelala C., Gribben J.G. (2013). Activated pancreatic stellate cells sequester CD8+ T cells to reduce their infiltration of the juxtatumoral compartment of pancreatic ductal adenocarcinoma. Gastroenterology.

[492] Wang E., Shibutani M., Nagahara H., Fukuoka T., Iseki Y., Okazaki Y., Kashiwagi S., Tanaka H., Maeda K., Hirakawa K. (2021). Abundant intratumoral fibrosis prevents lymphocyte infiltration into peritoneal metastases of colorectal cancer. PLoS ONE.

[493] Zhu Q., Zhang X., Zhang L., Li W., Wu H., Yuan X., Mao F., Wang M., Zhu W., Qian H. (2014). The IL-6-STAT3 axis mediates a reciprocal crosstalk between cancer-derived mesenchymal stem cells and neutrophils to synergistically prompt gastric cancer progression. Cell Death Dis..

[494] Du Y., Shao H., Moller M., Prokupets R., Tse Y.T., Liu Z.-J. (2019). Intracellular Notch1 Signaling in Cancer-Associated Fibroblasts Dictates the Plasticity and Stemness of Melanoma Stem/Initiating Cells. Stem Cells.

[495] Steele N.G., Biffi G., Kemp S., Zhang Y., Drouillard D., Syu L., Hao Y., Oni T., Brosnan E., Elyada E. (2021). Inhibition of Hedgehog signaling alters fibroblast composition in pancreatic cancer. Clin. Cancer Res..

[496] Miyai Y., Esaki N., Takahashi M., Enomoto A. (2020). Cancer-associated fibroblasts that restrain cancer progression: Hypotheses and perspectives. Cancer Sci..

[497] Wang S., Ma N., Kawanishi S., Hiraku Y., Oikawa S., Xie Y., Zhang Z., Huang G., Murata M. (2014). Relationships of alpha-SMA-positive fibroblasts and SDF-1-positive tumor cells with neoangiogenesis in nasopharyngeal carcinoma. Biomed. Res. Int..

[498] Ozdemir B.C., Pentcheva-Hoang T., Carstens J.L., Zheng X., Wu C.-C., Simpson T.R., Laklai H., Sugimoto H., Kahlert C., Novitskiy S.V. (2014). Depletion of carcinoma-associated fibroblasts and fibrosis induces immunosuppression and accelerates pancreas cancer with reduced survival. Cancer Cell.

[499] Lee J.J., Perera R.M., Wang H., Wu D.-C., Liu X.S., Han S., Fitamant J., Jones P.D., Ghanta K.S., Kawano S. (2014). Stromal response to Hedgehog signaling restrains pancreatic cancer progression. Proc. Natl. Acad. Sci. USA.

[500] Shin K., Lim A., Zhao C., Sahoo D., Pan Y., Spiekerkoetter E., Liao J.C., Beachy P.A. (2014). Hedgehog signaling restrains bladder cancer progression by eliciting stromal production of urothelial differentiation factors. Cancer Cell.

[501] Gerling M., Büller N.V.J.A., Kirn L.M., Joost S., Frings O., Englert B., Bergström Å., Kuiper R.V., Blaas L., Wielenga M.C.B. (2016). Stromal Hedgehog signalling is downregulated in colon cancer and its restoration restrains tumour growth. Nat. Commun..

[502] Pallangyo C.K., Ziegler P.K., Greten F.R. (2015). IKKβ acts as a tumor suppressor in cancer-associated fibroblasts during intestinal tumorigenesis. J. Exp. Med..

[503] Bhattacharjee S., Hamberger F., Ravichandra A., Miller M., Nair A., Affo S., Filliol A., Chin L., Savage T.M., Yin D. (2021). Tumor restriction by type I collagen opposes tumor-promoting effects of cancer-associated fibroblasts. J. Clin. Investig..

[504] Iida T., Mizutani Y., Esaki N., Ponik S.M., Burkel B.M., Weng L., Kuwata K., Masamune A., Ishihara S., Haga H. (2022). Pharmacologic conversion of cancer-associated fibroblasts from a protumor phenotype to an antitumor phenotype improves the sensitivity of pancreatic cancer to chemotherapeutics. Oncogene.

[505] Zheng X., Li J.-W., Liu Y.-K., Ma Y.-F., Gan J.-H., Han S.-G., Wang J., Wan Z.-Y., Zhang J., Liu Y. (2021). microRNA-10a-5p overexpression suppresses malignancy of colon cancer by regulating human liver cancer fibroblasts. Neoplasma.

[506] Glabman R.A., Choyke P.L., Sato N. (2022). Cancer-Associated Fibroblasts: Tumorigenicity and Targeting for Cancer Therapy. Cancers.

[507] Borzone F.R., Giorello M.B., Sanmartin M.C., Yannarelli G., Martinez L.M., Chasseing N.A. (2022). Mesenchymal stem cells and cancer-associated fibroblasts as a therapeutic strategy for breast cancer. Br. J. Pharmacol..

[508] Xu M., Zhang T., Xia R., Wei Y., Wei X. (2022). Targeting the tumor stroma for cancer therapy. Mol. Cancer.

[509] Kawada M., Inoue H., Usami I., Ikeda D. (2009). Phthoxazolin A inhibits prostate cancer growth by modulating tumor-stromal cell interactions. Cancer Sci..

[510] Straussman R., Morikawa T., Shee K., Barzily-Rokni M., Qian Z.R., Du J., Davis A., Mongare M.M., Gould J., Frederick D.T. (2012). Tumour micro-environment elicits innate resistance to RAF inhibitors through HGF secretion. Nature.

[511] Singh S., Lamichhane A., Rafsanjani Nejad P., Heiss J., Baumann H., Gudneppanavar R., Leipzig N.D., Konopka M., Luker G.D., Tavana H. (2022). Therapeutic Targeting of Stromal-Tumor HGF-MET Signaling in an Organotypic Triple-Negative Breast Tumor Model. Mol. Cancer Res..

[512] Qi L., Song F., Han Y., Zhang Y., Ding Y. (2020). Atractyloside targets cancer-associated fibroblasts and inhibits the metastasis of colon cancer. Ann. Transl. Med..

[513] Campos L.T., Brentani H., Roela R.A., Katayama M.L.H., Lima L., Rolim C.F., Milani C., Folgueira M.A.A.K., Brentani M.M. (2013). Differences in transcriptional effects of 1α,25 dihydroxyvitamin D3 on fibroblasts associated to breast carcinomas and from paired normal breast tissues. J. Steroid Biochem. Mol. Biol..

[514] Guan J., Zhang H., Wen Z., Gu Y., Cheng Y., Sun Y., Zhang T., Jia C., Lu Z., Chen J. (2014). Retinoic acid inhibits pancreatic cancer cell migration and EMT through the downregulation of IL-6 in cancer associated fibroblast cells. Cancer Lett..

[515] Han X., Li Y., Xu Y., Zhao X., Zhang Y., Yang X., Wang Y., Zhao R., Anderson G.J., Zhao Y. (2018). Reversal of pancreatic desmoplasia by re-educating stellate cells with a tumour microenvironment-activated nanosystem. Nat. Commun..

[516] Haubeiss S., Schmid J.O., Mürdter T.E., Sonnenberg M., Friedel G., van der Kuip H., Aulitzky W.E. (2010). Dasatinib reverses cancer-associated fibroblasts (CAFs) from primary lung carcinomas to a phenotype comparable to that of normal fibroblasts. Mol. Cancer.

[517] Jin Y., Kang Y., Peng X., Yang L., Li Q., Mei Q., Chen X., Hu G., Tang Y., Yuan X. (2021). Irradiation-Induced Activated Microglia Affect Brain Metastatic Colonization of NSCLC Cells via miR-9/CDH1 Axis. OncoTargets Ther..

[518] Wieder R. (2005). Insurgent micrometastases: Sleeper cells and harboring the enemy. J. Surg. Oncol..

[519] Tokes A.-M., Szasz A.M., Farkas A., Toth A.I., Dank M., Harsanyi L., Molnar B.A., Molnar I.A., Laszlo Z., Rusz Z. (2009). Stromal Matrix Protein Expression Following Preoperative Systemic Therapy in Breast Cancer. Clin. Cancer Res..

[520] Song Y., Kim S.-H., Kim K.M., Choi E.K., Kim J., Seo H.R. (2016). Activated hepatic stellate cells play pivotal roles in hepatocellular carcinoma cell chemoresistance and migration in multicellular tumor spheroids. Sci. Rep..

[521] Bellei B., Caputo S., Migliano E., Lopez G., Marcaccini V., Cota C., Picardo M. (2021). Simultaneous Targeting Tumor Cells and Cancer-Associated Fibroblasts with a Paclitaxel-Hyaluronan Bioconjugate: In Vitro Evaluation in Non-Melanoma Skin Cancer. Biomedicines.

[522] Yuan Y., Zhou C., Chen X., Tao C., Cheng H., Lu X. (2018). Suppression of tumor cell proliferation and migration by human umbilical cord mesenchymal stem cells: A possible role for apoptosis and Wnt signaling. Oncol. Lett..

[523] Melzer C., von der Ohe J., Hass R. (2018). Concise Review: Crosstalk of Mesenchymal Stroma/Stem-Like Cells with Cancer Cells Provides Therapeutic Potential. Stem Cells.

[524] Kraman M., Bambrough P.J., Arnold J.N., Roberts E.W., Magiera L., Jones J.O., Gopinathan A., Tuveson D.A., Fearon D.T. (2010). Suppression of Antitumor Immunity by Stromal Cells Expressing Fibroblast Activation Protein–α. Science.

[525] Loeffler M., Krüger J.A., Niethammer A.G., Reisfeld R.A. (2006). Targeting tumor-associated fibroblasts improves cancer chemotherapy by increasing intratumoral drug uptake. J. Clin. Investig..

[526] Su F., Wang X., Pearson T., Lee J., Krishnamurthy S., Ueno N.T., Kolonin M.G. (2020). Ablation of Stromal Cells with a Targeted Proapoptotic Peptide Suppresses Cancer Chemotherapy Resistance and Metastasis. Mol. Ther. Oncolytics.

[527] Yan M., Shen M., Xu L., Huang J., He G., An M., Li X., Gao Z., Meng X. (2020). Inactivation of Pancreatic Stellate Cells by Exendin-4 Inhibits the Migration and Invasion of Pancreatic Cancer Cells. OncoTargets Ther..

[528] Mediavilla-Varela M., Boateng K., Noyes D., Antonia S.J. (2016). The anti-fibrotic agent pirfenidone synergizes with cisplatin in killing tumor cells and cancer-associated fibroblasts. BMC Cancer.

[529] Aboulkheyr Es H., Zhand S., Thiery J.P., Warkiani M.E. (2020). Pirfenidone reduces immune-suppressive capacity of cancer-associated fibroblasts through targeting CCL17 and TNF-beta. Integr. Biol..

[530] Pure E., Lo A. (2016). Can Targeting Stroma Pave the Way to Enhanced Antitumor Immunity and Immunotherapy of Solid Tumors?. Cancer Immunol. Res..

[531] Wang Q., Yang W., Uytingco M.S., Christakos S., Wieder R. (2000). 1,25(OH)_2_ vitamin D_3_ and all-*trans* retinoic acid sensitize breast cancer cells to chemotherapy-induced cell death. Cancer Res..

[532] Wang Q., Lee D., Sysounthone V., Chandraratna R.A.S., Christakos S., Korah R., Wieder R. (2001). 1,25-dihydroxyvitamin D3 and retonic acid analogues induce differentiation in breast cancer cells with function- and cell-specific additive effects. Breast Cancer Res. Treat..

[533] Ding N., Yu R.T., Subramaniam N., Sherman M.H., Wilson C., Rao R., Leblanc M., Coulter S., He M., Scott C. (2013). A vitamin D receptor/SMAD genomic circuit gates hepatic fibrotic response. Cell.

[534] Ferrer-Mayorga G., Gómez-López G., Barbáchano A., Fernández-Barral A., Peña C., Pisano D.G., Cantero R., Rojo F., Muñoz A., Larriba M.J. (2017). Vitamin D receptor expression and associated gene signature in tumour stromal fibroblasts predict clinical outcome in colorectal cancer. Gut.

[535] Hwang R.F., Moore T.T., Hattersley M.M., Scarpitti M., Yang B., Devereaux E., Ramachandran V., Arumugam T., Ji B., Logsdon C.D. (2012). Inhibition of the hedgehog pathway targets the tumor-associated stroma in pancreatic cancer. Mol. Cancer Res..

[536] Yauch R.L., Gould S.E., Scales S.J., Tang T., Tian H., Ahn C.P., Marshall D., Fu L., Januario T., Kallop D. (2008). A paracrine requirement for hedgehog signalling in cancer. Nature.

[537] Olive K.P., Jacobetz M.A., Davidson C.J., Gopinathan A., McIntyre D., Honess D., Madhu B., Goldgraben M.A., Caldwell M.E., Allard D. (2009). Inhibition of Hedgehog signaling enhances delivery of chemotherapy in a mouse model of pancreatic cancer. Science.

[538] Chen Y., Hu M., Wang S., Wang Q., Lu H., Wang F., Wang L., Peng D., Chen W. (2022). Nano-delivery of salvianolic acid B induces the quiescence of tumor-associated fibroblasts via interfering with TGF-β1/Smad signaling to facilitate chemo- and immunotherapy in desmoplastic tumor. Int. J. Pharm..

[539] Grauel A.L., Nguyen B., Ruddy D., Laszewski T., Schwartz S., Chang J., Chen J., Piquet M., Pelletier M., Yan Z. (2020). TGFβ-blockade uncovers stromal plasticity in tumors by revealing the existence of a subset of interferon-licensed fibroblasts. Nat. Commun..

[540] Tanabe Y., Sasaki S., Mukaida N., Baba T. (2016). Blockade of the chemokine receptor, CCR5, reduces the growth of orthotopically injected colon cancer cells via limiting cancer-associated fibroblast accumulation. Oncotarget.

[541] Feig C., Jones J.O., Kraman M., Wells R.J.B., Deonarine A., Chan D.S., Connell C.M., Roberts E.W., Zhao Q., Caballero O.L. (2013). Targeting CXCL12 from FAP-expressing carcinoma-associated fibroblasts synergizes with anti-PD-L1 immunotherapy in pancreatic cancer. Proc. Natl. Acad. Sci. USA.

[542] Giordano C., Barone I., Vircillo V., Panza S., Malivindi R., Gelsomino L., Pellegrino M., Rago V., Mauro L., Lanzino M. (2016). Activated FXR Inhibits Leptin Signaling and Counteracts Tumor-promoting Activities of Cancer-Associated Fibroblasts in Breast Malignancy. Sci. Rep..

[543] Zheng J., Gao P. (2019). Toward Normalization of the Tumor Microenvironment for Cancer Therapy. Integr. Cancer Ther..

[544] Moon E.Y., Ryu Y.K., Lee G.H. (2014). Dexamethasone inhibits in vivo tumor growth by the alteration of bone marrow cd11b_+_ myeloid cells. Int. Immunopharmacol..

[545] Salmon H., Franciszkiewicz K., Damotte D., Dieu-Nosjean M.-C., Validire P., Trautmann A., Mami-Chouaib F., Donnadieu E. (2012). Matrix architecture defines the preferential localization and migration of T cells into the stroma of human lung tumors. J. Clin. Investig..

[546] Xu L.-Q., Lin M.-J., Li Y.-P., Li S., Chen S.-J., Wei C.-J. (2017). Preparation of Plasma Membrane Vesicles from Bone Marrow Mesenchymal Stem Cells for Potential Cytoplasm Replacement Therapy. J. Vis. Exp..

[547] Ogawa T., Kikuchi S., Tabuchi M., Mitsui E., Une Y., Tazawa H., Kuroda S., Noma K., Ohara T., Kagawa S. (2022). Modulation of p53 expression in cancer-associated fibroblasts prevents peritoneal metastasis of gastric cancer. Mol. Ther. Oncolytics.

[548] Sharma P., Singh S.S., Gayana S. (2021). Fibroblast activation protein inhibitor PET/CT: A promising molecular imaging tool. Clin. Nucl. Med..

[549] Dendl K., Schlittenhardt J., Staudinger F., Kratochwil C., Altmann A., Haberkorn U., Giesel F.L. (2021). The role of fibroblast activation protein ligands in oncologic pet imaging. PET Clin..

[550] Roustaei H., Kiamanesh Z., Askari E., Sadeghi R., Aryana K., Treglia G. (2022). Could fibroblast activation protein (FAP)-specific radioligands be considered as pan-tumor agents?. Contrast Media Mol. Imaging.

[551] Loktev A., Lindner T., Mier W., Debus J., Altmann A., Jäger D., Giesel F., Kratochwil C., Barthe P., Roumestand C. (2018). A Tumor-Imaging Method Targeting Cancer-Associated Fibroblasts. J. Nucl. Med..

[552] Kratochwil C., Flechsig P., Lindner T., Abderrahim L., Altmann A., Mier W., Adeberg S., Rathke H., Rohrich M., Winter H. (2019). 68ga-FAPI PET/CT: Tracer uptake in 28 different kinds of cancer. J. Nucl. Med..

[553] Loktev A., Lindner T., Burger E.M., Altmann A., Giesel F., Kratochwil C., Debus J., Marme F., Jager D., Mier W. (2019). Development of fibroblast activation protein-targeted radiotracers with improved tumor retention. J. Nucl. Med..

[554] Zhao L., Chen J., Pang Y., Fang J., Fu K., Meng L., Zhang X., Guo Z., Wu H., Sun L. (2022). Development of fibroblast activation protein inhibitor-based dimeric radiotracers with improved tumor retention and antitumor efficacy. Mol. Pharm..

[555] Roy J., Hettiarachchi S.U., Kaake M., Mukkamala R., Low P.S. (2020). Design and validation of fibroblast activation protein alpha targeted imaging and therapeutic agents. Theranostics.

[556] Zboralski D., Hoehne A., Bredenbeck A., Schumann A., Nguyen M., Schneider E., Ungewiss J., Paschke M., Haase C., von Hacht J.L. (2022). Preclinical evaluation of fap-2286 for fibroblast activation protein targeted radionuclide imaging and therapy. Eur. J. Nucl. Med. Mol. Imaging.

[557] Meng L., Fang J., Zhao L., Wang T., Yuan P., Zhao Z., Zhuang R., Lin Q., Chen H., Chen X. (2022). Rational design and pharmacomodulation of protein-binding theranostic radioligands for targeting the fibroblast activation protein. J. Med. Chem..

[558] Ding J., Xu M., Chen J., Zhang P., Huo L., Kong Z., Liu Z. (2022). 86Y-labeled albumin-binding fibroblast activation protein inhibitor for late-time-point cancer diagnosis. Mol. Pharm..

[559] Liu Y., Watabe T., Kaneda-Nakashima K., Shirakami Y., Naka S., Ooe K., Toyoshima A., Nagata K., Haberkorn U., Kratochwil C. (2022). Fibroblast activation protein targeted therapy using [177Lu]FAPI-46 compared with [225Ac]FAPI-46 in a pancreatic cancer model. Eur. J. Nucl. Med. Mol. Imaging.

[560] Li H., Ye S., Li L., Zhong J., Yan Q., Zhong Y., Feng P., Hu K. (2022). ^18^F- or ^177^Lu-labeled bivalent ligand of fibroblast activation protein with high tumor uptake and retention. Eur. J. Nucl. Med. Mol. Imaging.

[561] Boinapally S., Lisok A., Lofland G., Minn I., Yan Y., Jiang Z., Shin M.J., Merino V.F., Zheng L., Brayton C. (2022). Hetero-bivalent agents targeting fap and psma. Eur. J. Nucl. Med. Mol. Imaging.

[562] Hirata K., Yamaguchi S., Shiga T., Kuge Y., Tamaki N. (2019). The roles of hypoxia imaging using 18f-fluoromisonidazole positron emission tomography in glioma treatment. J. Clin. Med..

[563] Guo L., Shi D., Meng D., Shang M., Sun X., Zhou X., Liu X., Zhao Y., Li J. (2019). New FH peptide-modified ultrasonic nanobubbles for delivery of doxorubicin to cancer-associated fibroblasts. Nanomedicine.

[564] Dhawan U., Tseng C.L., Wang H.Y., Hsu S.Y., Tsai M.T., Chung R.J. (2021). Assessing suitability of co@au core/shell nanoparticle geometry for improved theranostics in colon carcinoma. Nanomaterials.

[565] Chen I.X., Chauhan V.P., Posada J., Ng M.R., Wu M.W., Adstamongkonkul P., Huang P., Lindeman N., Langer R., Jain R.K. (2019). Blocking CXCR4 alleviates desmoplasia, increases T-lymphocyte infiltration, and improves immunotherapy in metastatic breast cancer. Proc. Natl. Acad. Sci. USA.

[566] Ben-Ami E., Perret R., Huang Y., Courgeon F., Gokhale P.C., Laroche-Clary A., Eschle B.K., Velasco V., Le Loarer F., Algeo M.-P. (2020). LRRC15 Targeting in Soft-Tissue Sarcomas: Biological and Clinical Implications. Cancers.

[567] Chen Z., Wang Z., Jin T., Shen G., Wang Y., Tan X., Gan Y., Yang F., Liu Y., Huang C. (2019). Fibrogenic fibroblast-selective near-infrared phototherapy to control scarring. Theranostics.

[568] Zheng Z., Duan A., Dai R., Li Y., Chen X., Qin Y., Ren S., Li R., Cheng Z., Zhang R. (2022). A "dual-source, dual-activation" strategy for an NIR-II window theranostic nanosystem enabling optimal photothermal-ion combination therapy. Small.

[569] Hussain S., Peng B., Cherian M., Song J.W., Ahirwar D.K., Ganju R.K. (2020). The Roles of Stroma-Derived Chemokine in Different Stages of Cancer Metastases. Front. Immunol..

[570] Bijlsma M.F., van Laarhoven H.W.M. (2015). The conflicting roles of tumor stroma in pancreatic cancer and their contribution to the failure of clinical trials: A systematic review and critical appraisal. Cancer Metastasis Rev..

[571] Gieniec K.A., Butler L.M., Worthley D.L., Woods S.L. (2019). Cancer-associated fibroblasts-heroes or villains?. Br. J. Cancer.

[572] Xu M., Lin B., Zheng D., Wen J., Hu W., Li C., Zhang X., Zhang X., Qu J. (2021). LEM domain containing 1 promotes thyroid cancer cell proliferation and migration by activating the Wnt/β-catenin signaling pathway and epithelial-mesenchymal transition. Oncol. Lett..

[573] Ferrara B., Pignatelli C., Cossutta M., Citro A., Courty J., Piemonti L. (2021). The Extracellular Matrix in Pancreatic Cancer: Description of a Complex Network and Promising Therapeutic Options. Cancers.

[574] Moore G., Annett S., McClements L., Robson T. (2020). Top Notch Targeting Strategies in Cancer: A Detailed Overview of Recent Insights and Current Perspectives. Cells.

[575] Mizutani Y., Iida T., Ohno E., Ishikawa T., Kinoshita F., Kuwatsuka Y., Imai M., Shimizu S., Tsuruta T., Enomoto A. (2022). Safety and efficacy of MIKE-1 in patients with advanced pancreatic cancer: A study protocol for an open-label phase I/II investigator-initiated clinical trial based on a drug repositioning approach that reprograms the tumour stroma. BMC Cancer.

[576] Kawano S., Ito K., Yahata K., Kira K., Abe T., Akagi T., Asano M., Iso K., Sato Y., Matsuura F. (2019). A landmark in drug discovery based on complex natural product synthesis. Sci. Rep..

[577] Ueno H., Kajiwara Y., Ajioka Y., Sugai T., Sekine S., Ishiguro M., Takashima A., Kanemitsu Y. (2021). Histopathological atlas of desmoplastic reaction characterization in colorectal cancer. Jpn. J. Clin. Oncol..

[578] Jiang D., Chen X., You Z., Wang H., Zhang X., Li X., Ren S., Huang Q., Hua F., Guan Y. (2021). Comparison of [68 Ga]Ga-FAPI-04 and [18F]-FDG for the detection of primary and metastatic lesions in patients with gastric cancer: A bicentric retrospective study. Eur. J. Nucl. Med. Mol. Imaging.

[579] Von der Grun J., Winkelmann R., Burck I., Martin D., Rodel F., Wild P.J., Bankov K., Weigert A., Kur I.M., Brandts C. (2022). Neoadjuvant chemoradiotherapy for oral cavity cancer: Predictive factors for response and interim analysis of the prospective invert-trial. Front. Oncol..

[580] Andersen S., Richardsen E., Moi L., Donnem T., Nordby Y., Ness N., Holman M.E., Bremnes R.M., Busund L.-T. (2016). Fibroblast miR-210 overexpression is independently associated with clinical failure in Prostate Cancer—A multicenter (in situ hybridization) study. Sci. Rep..

[581] Sun J.D., Liu Q., Ahluwalia D., Li W., Meng F., Wang Y., Bhupathi D., Ruprell A.S., Hart C.P. (2015). Efficacy and safety of the hypoxia-activated prodrug TH-302 in combination with gemcitabine and nab-paclitaxel in human tumor xenograft models of pancreatic cancer. Cancer Biol. Ther..

[582] McLarty J., Bigelow R.L.H., Smith M., Elmajian D., Ankem M., Cardelli J.A. (2009). Tea Polyphenols Decrease Serum Levels of Prostate-Specific Antigen, Hepatocyte Growth Factor, and Vascular Endothelial Growth Factor in Prostate Cancer Patients and Inhibit Production of Hepatocyte Growth Factor and Vascular Endothelial Growth Factor In vitro. Cancer Prev. Res..

[583] Faivre S.J., Santoro A., Kelley R.K., Merle P., Gane E., Douillard J.-Y., Waldschmidt D., Mulcahy M.F., Costentin C., Minguez B. (2014). A phase 2 study of a novel transforming growth factor-beta (TGF-β1) receptor I kinase inhibitor, LY2157299 monohydrate (LY), in patients with advanced hepatocellular carcinoma (HCC). J. Clin. Oncol..

[584] Giannelli G., Villa E., Lahn M. (2014). Transforming Growth Factor-β as a Therapeutic Target in Hepatocellular Carcinoma. Cancer Res..

[585] Narra K., Mullins S.R., Lee H.-O., Strzemkowski-Brun B., Magalong K., Christiansen V.J., McKee P.A., Egleston B., Cohen S.J., Weiner L.M. (2007). Phase II trial of single agent Val-boroPro (talabostat) inhibiting fibroblast activation protein in patients with metastatic colorectal cancer. Cancer Biol. Ther..

[586] Hofheinz R.D., al-Batran S.E., Hartmann F., Hartung G., Jäger D., Renner C., Tanswell P., Kunz U., Amelsberg A., Kuthan H. (2003). Stromal Antigen Targeting by a Humanised Monoclonal Antibody: An Early Phase II Trial of Sibrotuzumab in Patients with Metastatic Colorectal Cancer. Oncol. Res. Treat..

[587] Stupp R., Hegi M.E., Gorlia T., Erridge S.C., Perry J., Hong Y.-K., Aldape K.D., Lhermitte B., Pietsch T., Grujicic D. (2014). Cilengitide combined with standard treatment for patients with newly diagnosed glioblastoma with methylated MGMT promoter (CENTRIC EORTC 26071-22072 study): A multicentre, randomised, open-label, phase 3 trial. Lancet Oncol..

[588] Benson A.B., Wainberg Z.A., Hecht J.R., Vyushkov D., Dong H., Bendell J., Kudrik F. (2017). A Phase II Randomized, Double-Blind, Placebo-Controlled Study of Simtuzumab or Placebo in Combination with Gemcitabine for the First-Line Treatment of Pancreatic Adenocarcinoma. Oncologist.

[589] Hecht J.R., Benson A.B., Vyushkov D., Yang Y., Bendell J., Verma U. (2017). A Phase II, Randomized, Double-Blind, Placebo-Controlled Study of Simtuzumab in Combination with FOLFIRI for the Second-Line Treatment of Metastatic KRAS Mutant Colorectal Adenocarcinoma. Oncologist.

[590] Sledge G.W. (2019). Targeting CXCR4-induced desmoplasia to improve checkpoint inhibition in breast cancer. Proc. Natl. Acad. Sci. USA.

[591] Pernas S., Martin M., Kaufman P.A., Gil-Martin M., Gomez Pardo P., Lopez-Tarruella S., Manso L., Ciruelos E., Perez-Fidalgo J.A., Hernando C. (2018). Balixafortide plus eribulin in HER2-negative metastatic breast cancer: A phase 1, single-arm, dose-escalation trial. Lancet Oncol..

[592] Lu Z., Weniger M., Jiang K., Boeck S., Zhang K., Bazhin A., Miao Y., Werner J., D’Haese J.G. (2018). Therapies Targeting the Tumor Stroma and the VEGF/VEGFR Axis in Pancreatic Ductal Adenocarcinoma: A Systematic Review and Meta-Analysis. Target. Oncol..

[593] Luo W. (2023). Nasopharyngeal Carcinoma Ecology Theory: Cancer as Multidimensional Spatiotemporal “Unity of Ecology and Evolution” Pathological Ecosystem. Theranostics.

[594] Kenny P.A., Nelson C.M., Bissell M.J. (2006). The ecology of tumors: By perturbing the microenvironment, wounds and infection may be key to tumor development. Scientist.

